# Spatiotemporal high-resolution prediction and mapping: methodology and application to dengue disease

**DOI:** 10.1007/s10109-021-00368-0

**Published:** 2022-02-19

**Authors:** I. Gede Nyoman Mindra Jaya, Henk Folmer

**Affiliations:** 1 Faculty of Spatial Sciences, University of Groningen, Groningen, The Netherlands; 2 Statistics Department, Padjadjaran University, Bandung, Indonesia

**Keywords:** Dengue disease, Relative risk, Fusion area-cell generalized geoadditive-Gaussian Markov random field model, Bayesian statistics, “Big *n*, Problem, Bottom-up approach, C18, I18

## Abstract

Dengue disease has become a major public health problem. Accurate and precise identification, prediction and mapping of high-risk areas are crucial elements of an effective and efficient early warning system in countering the spread of dengue disease. In this paper, we present the fusion area-cell spatiotemporal generalized geoadditive-Gaussian Markov random field (FGG-GMRF) framework for joint estimation of an area-cell model, involving temporally varying coefficients, spatially and temporally structured and unstructured random effects, and spatiotemporal interaction of the random effects. The spatiotemporal Gaussian field is applied to determine the unobserved relative risk at cell level. It is transformed to a Gaussian Markov random field using the finite element method and the linear stochastic partial differential equation approach to solve the “big *n*” problem. Sub-area relative risk estimates are obtained as block averages of the cell outcomes within each sub-area boundary. The FGG-GMRF model is estimated by applying Bayesian Integrated Nested Laplace Approximation. In the application to Bandung city, Indonesia, we combine low-resolution area level (district) spatiotemporal data on population at risk and incidence and high-resolution cell level data on weather variables to obtain predictions of relative risk at subdistrict level. The predicted dengue relative risk at subdistrict level suggests significant fine-scale heterogeneities which are not apparent when examining the area level. The relative risk varies considerably across subdistricts and time, with the latter showing an increase in the period January–July and a decrease in the period August–December.

**Supplementary Information:**

The online version contains supplementary material available at 10.1007/s10109-021-00368-0.

## Introduction

Dengue disease is a major challenge to healthcare worldwide, potentially leading to death, especially among the poor in low- and middle-income countries (Ak et al. 2018). In addition, there are latent costs related to stress, productivity loss, school absence, and care taking (Wilastonegoro et al. 2020). To reduce health impacts and treatment costs, there have been substantial efforts aimed at the prevention of dengue disease outbreaks (Kampen et al. 2014). For these purposes, statistical models aimed at identifying the causes, transmission mechanisms, and prediction of outbreaks at a fine spatiotemporal scale are of crucial importance (Messina et al. 2019).

High-resolution information is needed for research on the etiology of the disease and the development of control and prevention strategies (Ak et al. 2018; Jaya et al. 2017; Jaya and Folmer 2020, 2021a, b; Pokharel and Deardon 2016). Dengue disease typically involves a spatiotemporal pattern (Jaya and Folmer 2020, 2021a, b; Phanitchat et al. 2019; Puggioni et al. 2020); therefore, high-resolution spatiotemporal models and maps of the distribution of relative risk are basic elements of an early warning system aimed at identifying where and when an outbreak will occur (Hanigan et al. 2019; Shi et al. 2013; Xu et al. 2019).

In many areas of spatial research including epidemiology, data are often only available at different levels of aggregation (Moraga et al. 2017; Shi et al. 2013; Utazi et al. 2019). If low-resolution information is available whereas high-resolution (cell level) information is needed but lacking, area-to-cell disaggregation through joint area-cell estimation can be applied to obtain the missing information (Moraga et al. 2017; Utazi et al. 2019; Wang et al. 2018a).^1^ Data fusion or data assimilation (Banerjee et al. 2015) and Bayesian melding (Fuentes and Raftery 2005; Liu et al. 2011) are terms used to denote integrating multiple data sources of different spatial resolutions.^2^ The basic concept involves combining area and cell observations in a single statistical model. Combining data measured at different levels of aggregation can improve parameter estimation and increase prediction accuracy (Wang et al. 2018b). However, it may lead to spatial misalignment (Moraga et al. 2017; Sahu et al. 2010; Truong et al. 2014; Utazi et al. 2019), which induces biased or inconsistent estimators (Liu and Bertazzon 2016; Peng and Bell 2010; Saez and López-Casasnovas 2019).

Several kinds of correction approaches based on regression models have been developed to deal with area-to-cell misalignment problems (Banerjee and Gelfand 2002; Banerjee et al. 2015). Moraga et al. (2017) and Utazi et al. (2019) applied Bayesian geostatistical analysis to deal with misalignment in spatial non-Gaussian data with linear covariates. However, methods that address area-to-cell misalignment in spatiotemporal non-Gaussian data with nonlinear covariates are less well known. This applies especially to Poisson or Negative Binomial (NB) spatiotemporal data, which are typically applied in dengue and other disease incidence modeling, but also in other kinds of spatial and regional research.

In this paper, we introduce the Fusion Area-Cell Spatiotemporal Generalized Geoadditive (GG)-Gaussian Field (GF) model, abbreviated as FGG-GF model, to generate high-resolution (cell) predictions based on observations at lower (area) resolution of the variable of interest (i.e., the number of dengue incidences) and the population at risk, and high-resolution cell data (i.e., weather variables), while controlling for misalignment. Moraga et al. (2017) and Utazi et al. (2019) have shown that the prediction performance of integrated area-cell models can outperform models that use single level data sources.

Kammann and Wand (2003) introduced the Generalized Geoadditive Model (GGM), which has become popular in disease mapping (among other fields) because of its suitability for making high-resolution maps (Muleia et al. 2020; Wand et al. 2011). The model assumes that there is a spatially continuous variable underlying all observations which can be modeled using a Gaussian process, usually denoted Gaussian Field (GF). A GF is characterized by a first-order autoregressive model with spatially correlated innovations. The GGM combines the Generalized Additive Model (GAM) and the Geostatistical Model (GM). The former was introduced by Hastie and Tibshirani (1986) to provide a flexible means of handling nonlinear and interacting covariates. GAMs are also suitable for handling complex spatial and temporal autocorrelation (French and Wand 2004; Ma et al. 2014). GAMs are nonparametric because they do not require a priori specification of the regression function (Wang et al. 2018a). The GM was introduced by Matheron (1963) to construct high-resolution maps over a particular geographical region based on cell data on (risk) factors associated with a (dependent) variable of interest.

The integrated area-cell observations and the combination of non-Gaussian data, a nonlinear predictor and latent model components, in particular the spatiotemporal GF, make estimation of FGG-GF model, prediction, and mapping computationally complex and time-consuming (Barber et al. 2016) because of the “big *n*” problem. This issue can be handled by transforming a GF with a dense covariance matrix to a Gaussian Markov Random Field (GMRF) with a sparse precision matrix^3^ of Matérn covariances (Lindgren et al. 2011).

The objective of this paper is to develop a high-resolution prediction and mapping procedure for spatiotemporal Poisson or Negative Binomial data applying a Fusion Area-Cell Spatiotemporal Generalized Geoadditive-Gaussian Markov Random Field (abbreviated as FGG-GMRF) model. Inference and prediction are handled in a Bayesian framework. The approach will subsequently be applied to dengue disease risk in Bandung city, Indonesia. The purpose is to predict and map the relative dengue risk at subdistrict level, given observations on dengue incidence and population at risk at district level and weather risk factors at cell level.^4^ Special attention is paid to high-risk districts and subdistricts requiring public intervention (Aguayo et al. 2020).

The structure of the remainder of this paper is as follows. Section 2 introduces the spatiotemporal GG-GF model. Section 3 presents the FGG-GF and FGG-GMRF models and the Bayesian inference framework. The link between the GF and GMRF models is summarized in Appendix 1. Section 4 applies the methodology to dengue incidence in Bandung city, Indonesia, and Sect. 5 summarizes and concludes the conducted research.

## The spatiotemporal generalized geoadditive-Gaussian field model

Consider region $\mathcal{A}\in {\mathbb{R}}^{2}$*,* partitioned into ${n}_{\mathcal{A}}$ areas (e.g., districts in a city), each measured for $T$ periods. The areas are labeled $\left\{\left({\mathcal{A}}_{1},1\right),\ldots ,\left({\mathcal{A}}_{1},T\right),\left({\mathcal{A}}_{2},1\right),\ldots ,\left({\mathcal{A}}_{i},t\right),\ldots ,\left({\mathcal{A}}_{{n}_{\mathcal{A}}},T\right)\right\}$, where $\left({\mathcal{A}}_{i},t\right)$ denotes area $i$ at time $t,$ for $i=1,\ldots ,{n}_{\mathcal{A}}$ and $t=1,\ldots ,T$. Region $\mathcal{A}$ is further divided into a finite set of ${n}_{p}$ cells for $T$ periods. The set of ${n}_{p}$ cells over $T$ periods is denoted $\left\{\left({{\varvec{s}}}_{1},1\right),\ldots ,\left({{\varvec{s}}}_{{n}_{{\mathcal{A}}_{1}}},T\right),\left({{\varvec{s}}}_{{n}_{{\mathcal{A}}_{1}}+1},1\right),\ldots ,\left({{\varvec{s}}}_{g},t\right),\ldots ,\left({{\varvec{s}}}_{{n}_{p}},T\right)\right\},$ with ${n}_{{\mathcal{A}}_{i}}$ denoting the number of cells in area ${\mathcal{A}}_{i}$ for $g=1,\ldots ,{n}_{p}$ and $t=1,\ldots ,T \,{{\rm and}}\,n_{p}=\sum_{i=1}^{{n}_{\mathcal{A}}}{n}_{{\mathcal{A}}_{i}}$. Note that the notation $\left\{\left({{\varvec{s}}}_{1\left({\mathcal{A}}_{1}\right)},1\right),\ldots ,\left({{\varvec{s}}}_{{n}_{{\mathcal{A}}_{1}}\left({\mathcal{A}}_{1}\right)},T\right),\left({{\varvec{s}}}_{{n}_{{\mathcal{A}}_{1}}+1\left({\mathcal{A}}_{2}\right)},1\right),\ldots ,\left({{\varvec{s}}}_{g\left({\mathcal{A}}_{i}\right)},t\right),\ldots ,\left({{\varvec{s}}}_{{n}_{p}\left({\mathcal{A}}_{{n}_{\mathcal{A}}}\right)},T\right)\right\}$ will be used to explicitly denote that cell ${{\varvec{s}}}_{g\left({\mathcal{A}}_{i}\right)}$ belongs to area ${\mathcal{A}}_{i}$. If the area is not relevant, ${{\varvec{s}}}_{g}$ will be used. Moreover, the notation $g$ for ${\mathbf{s}}_{g}$ will be incidentally used if there is no risk of misunderstanding. Finally, ${s}_{g,1}$ and ${s}_{g,2}$ denote the latitude and longitude coordinates of its centroid, respectively.

Let ${y}_{it}$ and ${N}_{it}$ denote the number of (dengue) incidences and population at risk in area ${\mathcal{A}}_{i}$ at time $t$, respectively, and ${y}_{gt}$ and ${N}_{gt}$ the number of (dengue) incidences and population at risk in cell ${\mathbf{s}}_{g}$ at time $t$, respectively. Note that both ${y}_{gt}$ and ${N}_{gt}$ are unobserved at the cell level. ${y}_{it}$ and ${y}_{gt}$ are assumed to follow Poisson distributions^5^ with means ${\mu }_{it}={E}_{it}{\theta }_{it}$ and ${\mu }_{gt}={E}_{gt}{\theta }_{gt}$, respectively, with ${E}_{it}$ and ${E}_{gt}$ denoting the expected number of (dengue) incidences and ${\theta }_{it}$ and ${\theta }_{gt}$ the relative (dengue) risk for area $i$ and cell $g$ at time $t$, respectively (Jaya and Folmer 2020. The expected rate ${E}_{it}$ is calculated using external standardization. It is defined based on the overall average across all areas and periods (Abente et al. 2018; Jaya and Folmer 2020, 2021a, b):

1 $${E}_{it}={N}_{it}\left(\frac{1}{{n}_{\mathcal{A}}T}\sum_{i=1}^{{n}_{\mathcal{A}}}\sum_{t=1}^{T}{y}_{it}/\frac{1}{{n}_{\mathcal{A}}T}\sum_{i=1}^{{n}_{\mathcal{A}}}\sum_{t=1}^{T}{N}_{it}\right) \quad {{\rm for}}\,i=1,\ldots ,{n}_{\mathcal{A}}\,{{\rm and}}\,t=1,\ldots ,T.$$

The relative risk is defined as the ratio of the local risk in a spatiotemporal unit relative to the average risk across the whole study region over the entire time period (Yin et al. 2014). It is centered around one, meaning that the total number of incidences is equal to the expected rate. The maximum likelihood (ML) estimator of the relative risk ${\theta }_{it}$ is (Jaya et al. 2017; Jaya and Folmer 2020):

2 $${\widehat{\theta }}_{it}=\frac{{y}_{it}}{{E}_{it}} \quad {{\rm for}}\,i=1,\ldots ,{n}_{\mathcal{A}}\,{{\rm and}}\,t=1,\ldots ,T.$$

This is known as the crude risk or the standardized incidence ratio (SIR).

Following Moraga et al. (2017), Jaya and Folmer (2020, 2021a, b), and Utazi et al. (2019), we model the relative risk as a non-separable Poisson log-linear model as follows^6^:

3a $${\eta }_{it}={\beta }_{0}+\sum_{k=1}^{K}{f}_{k}\left({\overline{{\rm x}} }_{k,it}\right)+{\omega }_{i}+{\upsilon }_{i}+{\phi }_{t}+{\varsigma }_{t}+{\delta }_{it}+{\overline{\Phi } }_{it}\,\,{\rm for}\,\,i=1,\ldots ,{n}_{\mathcal{A}}\,{{\rm and}}\, t=1,\ldots ,T,$$

3b $${\eta }_{gt}={\beta }_{0}+\sum_{k=1}^{K}{f}_{k}\left({{\rm x}}_{k,gt}\right)+{\omega }_{g({\mathcal{A}}_{i})}+{\upsilon }_{g({\mathcal{A}}_{i})}+{\phi }_{t}+{\varsigma }_{t}+{\delta }_{g({\mathcal{A}}_{i})t}+{\Phi }_{gt}\,\,{\rm for}\,\,g=1,\ldots ,{n}_{p}\,{{\rm and}}\,t =1,\ldots ,T$$

with $${\eta }_{it}={\rm log}\left({\theta }_{it}\right)$$ and $${\eta }_{gt}={\rm log}\left({\theta }_{gt}\right)$$.

In Eqs. (3a) and (3b), ${\beta }_{0}$ is the overall intercept denoting the average risk across space and time, i.e., across all $i=1,\ldots ,{n}_{\mathcal{A}}$, $g=1,\ldots ,{n}_{p}$, and $t=1,\ldots ,T$. The latent^7^ functions ${f}_{k}\left({\overline{{\rm x}} }_{k,it}\right)$ and ${f}_{k}\left({{\rm x}}_{k,gt}\right)$ for $k=1,\ldots ,K$, represent the (non)linear effects of the metrical area and cell risk factors, respectively. The risk factors at cell level for a given area ${\mathcal{A}}_{i}$ and time $t$ are fixed. However, they vary across areas and times. The latent (non)linear risk factor functions are based on observations at cell level but are predicted at area level. The risk at cell and area levels are assumed to be driven by the same factors; therefore, we adopt joint risk factor functions. For this purpose, we stack the observations on the risk factors such that risk factor $k$, at both area and cell level, becomes ${\mathbf{z}}_{k}={\left({\overline{{\rm x}} }_{k,11},\ldots {\overline{{\rm x}} }_{k,{n}_{\mathcal{A}}T},{{\rm x}}_{k,11},\ldots ,{{\rm x}}_{k,{n}_{p}T}\right)}^{{\prime}}$ for $k=1,\ldots ,K$ and latent function ${f}_{k}\left({\mathbf{z}}_{k}\right)$. The functions ${f}_{k}\left({{\varvec{z}}}_{k}\right)$ are commonly centered at the mean, i.e., ${\mathbb{E}}\left[{f}_{k}\left({{\varvec{z}}}_{k}\right)\right]=0,$ for identifiability reasons (Fahrmeir and Lang 2001).

Let $f({\varvec{z}})$ be the sum of the functions ${f}_{k}({{\varvec{z}}}_{k})$ for $k=1,\ldots ,K$:

4 $$f\left({\varvec{z}}\right)=\sum_{k=1}^{K}{f}_{k}\left({{\varvec{z}}}_{k}\right)={f}_{1}\left({{\varvec{z}}}_{1}\right)+\cdots +{f}_{K}\left({{\varvec{z}}}_{K}\right).$$

To account for spatiotemporal variation, Eq. (4) can be extended to a varying coefficients model^8^:

5 $$f\left({\varvec{z}}\right)={f}_{1}\left({{\varvec{z}}}_{1}\right){\mathbf{v}}_{1}+\cdots +{f}_{K}({{\varvec{z}}}_{K}){\mathbf{v}}_{K}$$

where the design vector $\mathbf{v}=\left({\mathbf{v}}_{1},\ldots ,{\mathbf{v}}_{K}\right)\boldsymbol{^{\prime}}$ contains components of ${\varvec{z}}$ or additional covariates. The vector ${\mathbf{v}}_{k} \quad {{\rm for}}\,k=1, \ldots ,K$, modifies the relationship between the covariate ${{\varvec{z}}}_{k}$ and the log-linear conditional expectation ${\mathbb{E}}\left[\mathbf{y}|{\varvec{z}}\right]$. If it is identical to the vector **1**, i.e., ${\mathbf{v}}_{k}={\left(1,\ldots ,1\right)}^{{\prime}}$ with dimension (${n}_{\mathcal{A}}+{n}_{p})T\times 1$, then ${f}_{k}({{\varvec{z}}}_{k})$ presents the overall (main) effect of ${{\varvec{z}}}_{k}.$ If it is different from $1,{f}_{k}\left({{\varvec{z}}}_{k}\right){\mathbf{v}}_{k}$ presents the effect of ${\mathbf{z}}_{k}$ that varies along with ${\mathbf{v}}_{k}$. In other words, ${f}_{k}\left({{\varvec{z}}}_{k}\right){\mathbf{v}}_{k}$ models the interaction between ${\mathbf{z}}_{k}$ and ${\mathbf{v}}_{k}$ (Fahrmeir and Lang 2001). According to Martınez-Bello et al. (2017a; b), the varying coefficients model helps refine the association between the regressors (e.g., the weather variables) and the response, thus improving predictions at a fine spatiotemporal scale. For example, if ${\mathbf{v}}_{k}$ denotes the calendar day and ${\mathbf{z}}_{k}$ is the spatiotemporal covariate temperature, then ${f}_{k}\left({{\varvec{z}}}_{k}\right){\mathbf{v}}_{k}$ represents the temperature effect varying by day. In this study, we apply the temporally varying coefficients model to accommodate the temporally varying nonlinear effects of risk factors on the response. The time-varying effect of, for example, the $k$-th covariate can be written as (Franco-Villoria et al. 2019):

6 $${f}_{k}\left({{\varvec{z}}}_{k,t}\right){\mathbf{v}}_{k,t}={\beta }_{k}\left({\mathbf{v}}_{k,t}\right){{\varvec{z}}}_{k,t}\, {\rm for\, every }\,i\,{\rm and }\,g,\,{\rm and\,for }\,k=1,\ldots ,K\,{{\rm and}}\,t=1,\ldots ,T.$$

where ${\beta }_{k}({\mathbf{v}}_{k,t})$ for $t=1,\ldots ,T$ is the time-varying regression coefficient, which can be regarded as a stochastic process over ${\mathbf{v}}_{k}$ (Fahrmeir and Lang 2001). For ease of notation, we ignore the term ${\mathbf{v}}_{k}$ and write ${\beta }_{k,t}$.

A time-varying coefficient can be conveniently specified as the sum of a fixed (global mean) effect and a temporal random effect of the risk factor:${\beta }_{k,t}={\beta }_{k}+{\zeta }_{k,t}$ for $k=1,\ldots ,K$ and $t=1,\ldots ,T$. The fixed effect (${\beta }_{k}$) presents the effect of the risk factor that remains constant across space or time, while the temporal random effect (${\zeta }_{k,t})$ accounts for the time-varying effect of the risk factor (Song et al. 2020). The temporal random effect ${\zeta }_{k,t}$ can be conveniently specified as a random walk model of order one (RW1) or two (RW2)^9^ (Bernardinelli et al. 1995; Martinez-Bello et al. 2017b; Schrödle and Knorr-Held 2011):

7 $${\zeta }_{k,t}={\zeta }_{k,t-1}+{u}_{k,t} ({\rm RW}1)\,{\rm for }\,t=2,\ldots ,T\,{\rm or }\,{\zeta }_{k,t}=2{\zeta }_{k,t-1}-{\zeta }_{k,t-2}+{u}_{k,t} ({\rm RW}2)\,{\rm for}\, t=3,\ldots ,T$$

with ${u}_{k,t}\sim \mathcal{N}\left(0,{\sigma }_{{\zeta }_{k}}^{2}\right)$ white noise, and ${\sigma }_{{\zeta }_{k}}^{2}$ denoting the variance of the RW process controlling the smoothness of ${\beta }_{k,t}$. A random walk process of order one needs an initial value of ${\zeta }_{k,1}$ and a random walk of order two needs initial values of ${\zeta }_{k,1}$ and ${\zeta }_{k,2}$.

The components ${\omega }_{i}$ and ${\upsilon }_{i}$ are the spatially structured and unstructured main effects at area level, respectively, whereas ${\omega }_{{g(\mathcal{A}}_{i})}$ and ${\upsilon }_{{g(\mathcal{A}}_{i})}$ denote the spatially structured and unstructured main effects for cell ${s}_{g}$ in the area ${\mathcal{A}}_{i}$ to which it belongs. The components ${\phi }_{t}$ and ${\varsigma }_{t}$ are the temporally structured and unstructured main effects. For a given time $t,$ they are equal for all areas and cells. ${\delta }_{it}$ represents the spatiotemporal interaction effect of the unobserved risk factors at area level and ${\delta }_{{g(\mathcal{A}}_{i})t}$ the impact of the interaction effect ${\delta }_{it}$ in cell ${s}_{g}$ in area ${\mathcal{A}}_{i}$ to which it belongs (see Sect. 3.1 for details). We consider four type of space–time interactions (see Table 5 in Appendix 2).

The final component, ${\Phi }_{gt},$ in Eq. (3b) is the spatiotemporal GF in cell ${\mathbf{s}}_{g }\,{\rm for}\, g=1,\ldots ,{n}_{p}$ at time $t$, indicating the true but unobserved relative risk (Cameletti et al. 2013; Godana et al. 2019). Hence, ${\Phi }_{gt}$ is the “own” spatiotemporal interaction effect of cell ${\mathbf{s}}_{g}$. Because of the large number of cells, it is continuously indexed (Blangiardo and Cameletti 2015). The component ${\overline{\Phi } }_{it}$ in Eq. (3a) denotes the area average of ${\Phi }_{gt}$ across the cells within ${\mathcal{A}}_{i}$. Following Cameletti et al. (2013) and Godana et al. (2019), we assume that $\Phi \left({{\varvec{s}}}_{g},t\right)$ changes over time following a first-order autoregressive (AR1) process with coefficient ${\lambda }_{1}, \left| {\lambda }_{1}\right|<1$:

8 $$\Phi \left({{\varvec{s}}}_{g},t\right)={\lambda }_{1}\Phi \left({{\varvec{s}}}_{g},t-1\right)+\gamma \left({{\varvec{s}}}_{g},t\right) \quad {{\rm for}}\,t=2,\ldots ,T\,{{\rm and}}\,g=1,\ldots ,{n}_{p}$$

with $\Phi \left({{\varvec{s}}}_{g},1\right)\sim \mathcal{N}\left(0,{\sigma }_{\Phi }^{2}/\left(1-{\lambda }_{1}^{2}\right)\right)\,{{\rm and}}\,\gamma \left({{\varvec{s}}}_{g},t\right)$ defined as a mean square differentiable process^10^ (Stein 1999) with the temporally independent but spatially correlated innovations following a zero-mean Gaussian distribution with spatiotemporal covariance function:

9 $${\rm Cov}\left(\gamma \left({{\varvec{s}}}_{g},t\right),\gamma \left({{\varvec{s}}}_{h},t{^{\prime}}\right)\right)=\left\{\begin{array}{ll}0 & \quad {\rm if}\,\, t\ne t{^{\prime}}\\ {\sigma }_{\Phi }^{2}R\left(d\right) & \quad {\rm if}\,\, t=t{^{\prime}}\end{array}\right.$$

for $g\ne h.$ ${\sigma }_{\Phi }^{2}$ is the homogeneous variance of $\gamma \left({{\varvec{s}}}_{g},t\right)$, i.e., ${\rm Var}\left(\gamma \left({{\varvec{s}}}_{g},t\right)\right)={\sigma }_{\Phi }^{2}$ for every ${{\varvec{s}}}_{g}$ and $t$, and $\mathcal{R}\left(d\right)$ the spatial autocorrelation matrix as a function of the distance $d$ between ${{\varvec{s}}}_{g}$ and ${{\varvec{s}}}_{h}$ at time $t$ (e.g., the Euclidean distance). Under the assumption that the covariance function only depends on $d$, it is a Matérn covariance function satisfying the second-order stationarity and isotropy assumptions. Consequently, the mean of the process is constant and only depends on the locations of ${{\varvec{s}}}_{g}$ and ${{\varvec{s}}}_{h}$ through the Euclidean distance $d=\Vert {{\varvec{s}}}_{g}-{{\varvec{s}}}_{h}\Vert \in {\mathbb{R}}$ (Song et al. 2008). The spatial autocorrelation function $\mathcal{R}\left(d\right)$ is defined as:

10 $$\mathcal{R}\left(d\right)=\frac{1}{\Gamma \left(v\right){2}^{v-1}}{\left(\kappa d\right)}^{v}{K}_{v}\left(\kappa d\right) \quad {\rm for\, every}\, t$$

where $\Gamma \left(.\right)$ is the gamma function, ${K}_{v}\left(.\right)$ the modified Bessel function of the second order (Abramovitz and Stegun 1965) and $v>0$ the parameter controlling the smoothness of the GF (smoothness parameter). In applications, $v$ is commonly fixed because it is usually poorly identified (Miller et al. 2019; Utazi et al. 2019). In several software packages, including R-INLA (Integrated Nested Laplace Approximation), the default value is ${\rm one} (v$ = 1), corresponding to moderate smoothness (Lindgren et al. 2011; Utazi et al. 2019). The scale parameter $\kappa , \kappa >0$ controls the rate of decay of the correlation and is inversely related to the range parameter $r$ of the Euclidean distance between $\gamma ({{\varvec{s}}}_{g},t)$ and $\gamma \left({{\varvec{s}}}_{h},t\right).$ For large $r$, $\kappa$ goes to zero. Because of a lack of a simple relationship between $\kappa$ and $r$, Lindgren et al. (2011) proposed the empirically derived relationship $r=\sqrt{8v}/\kappa$ for spatial autocorrelation near 0.1. Substituting Eq. (10) in Eq. (9), the spatiotemporal Matérn covariance function $\Sigma \left(d\right)$ for each time $t$ is:

11 $$\Sigma \left(d\right)=\frac{{\sigma }_{\Phi }^{2}}{\Gamma \left(v\right){2}^{v-1}}{\left(\kappa d\right)}^{v}{K}_{v}\left(\kappa d\right) \quad{\rm for\,\, every }\,t.$$

For the joint latent spatiotemporal GF ${{\varvec{\Phi}}}_{t}={\left({\Phi }_{1t},\ldots ,{\Phi }_{{n}_{p}t}\right)}^{{{\prime}}}$ at cell level, we have:

12 $${{\varvec{\Phi}}}_{t}={\lambda }_{1}{{\varvec{\Phi}}}_{t-1}+{{\varvec{\upgamma}}}_{t}\,{{\rm and}}\,{{\varvec{\upgamma}}}_{t}\sim \mathcal{N}\left(0,{\varvec{\Sigma}}\right) \quad {\rm for }\,t=2,\ldots ,T.$$

with ${{\varvec{\upgamma}}}_{t}=({\gamma }_{1,t},\ldots ,{\gamma }_{{n}_{p},t}){^{\prime}}$ ${\rm and}\,\,{\varvec{\Sigma}}={\sigma }_{\Phi }^{2}\mathcal{R}$ a Matérn covariance matrix. That is, the joint latent spatiotemporal GF is a second-order stationary, isotropic GF with Matérn covariance function Eq. (11) and initial value distributed as ${{\varvec{\Phi}}}_{1}\sim \mathcal{N}\left(0,\frac{{\sigma }_{\Phi }^{2}}{\left(1-{\lambda }_{1}^{2}\right)}\mathcal{R}\right)$.

## Bayesian inference

This section consists of two subsections. In the first, we present the Fusion Area-Cell Spatiotemporal Generalized Geoadditive-Gaussian Field (FGG-GF) model which integrates the sub-models (3a) and (3b) into a single statistical model. The section also presents the Bayesian statistical tools. In the second section, we discuss solving the “big *n*” problem resulting in the Fusion Area-Cell Spatiotemporal Generalized Geoadditive-Gaussian Markov Random Field (FGG-GMRF) model and point out that it can be estimated using the R-INLA package. Details on the link between the GF and the GMRF through the Linear Stochastic Partial Differential Equation (LSPDE) approach are discussed in Appendix 1.

### The fusion area-cell spatiotemporal generalized geoadditive-Gaussian field model

As observed above, combining low-resolution and high-resolution data to generate high-resolution predictions entails the risk of misalignment (Moraga et al. 2017; Utazi et al. 2019). To handle misalignment, we first stack the corresponding objects of the area and cell models in Eqs. (3a) and (3b) to give the FGG-GF model^11^ which is then estimated as a single model (Blangiardo and Cameletti 2015; Kifle et al. 2017; Utazi et al. 2019). The FGG-GF model reads:

13 $${{\varvec{\upeta}}}_{t}={\upbeta }_{0}{1}_{({n}_{\mathcal{A}}+{n}_{p})}+{\sum }_{k=1}^{K}\left({\beta }_{k}+{\zeta }_{k,t}\right){\mathbf{z}}_{k,t}+\ddot{{\varvec{\upomega}}}+\ddot{{\varvec{\upsilon}}}+{\phi }_{t}{1}_{({n}_{\mathcal{A}}+{n}_{p})}+{\varsigma }_{t}{1}_{({n}_{\mathcal{A}}+{n}_{p})}+{\ddot{{\varvec{\updelta}}}}_{t}+{\ddot{{\varvec{\Phi}}}}_{t} \quad {\rm for }\,t=1, \ldots ,T$$

where ${{\varvec{\upeta}}}_{t}={\left({\eta }_{1,t},\ldots ,{\eta }_{{n}_{\mathcal{A}},t},{\eta }_{\left({n}_{\mathcal{A}}+1\right),t},\ldots ,{\eta }_{\left({n}_{\mathcal{A}}+{n}_{p}\right),t}\right)}^{{\prime}}$, ${\beta }_{0}$ the global mean defined in Eq. (3), ${1}_{\left({n}_{\mathcal{A}}+{n}_{p}\right)}$ a vector of ones of dimension $\left({n}_{\mathcal{A}}+{n}_{p}\right)$, ${\mathbf{z}}_{k,t}={\left({\mathbf{z}}_{k,1t},\ldots ,{\mathbf{z}}_{k,{n}_{\mathcal{A}}t},{\mathbf{z}}_{k,\left({n}_{\mathcal{A}}+1\right)t },\ldots ,{\mathbf{z}}_{k,\left({n}_{\mathcal{A}}+{n}_{p}\right)t}\right)}^{{{\prime}}}$ the joint $k$ th risk factor with fixed coefficient ${\beta }_{k}$ and temporal random coefficient ${{\varvec{\zeta}}}_{k}=\left({\zeta }_{k,1},\ldots ,{\zeta }_{k,T}\right){^{\prime}}{\rm for} k=1,\ldots ,K$. Furthermore, $\ddot{{\varvec{\upomega}}}={\left({\varvec{\upomega}},{{\varvec{\upomega}}}_{\mathcal{A}}\right)}^{{\prime}}$, with ${\varvec{\upomega}}={\left({\omega }_{1},\ldots .,{\omega }_{i},\ldots ,{\omega }_{{n}_{\mathcal{A}}}\right)}^{{{\prime}}}$ and ${{\varvec{\upomega}}}_{\mathcal{A}}={\left({\omega }_{1\left({\mathcal{A}}_{1}\right)},\ldots ,{\omega }_{g\left({\mathcal{A}}_{i}\right)}\ldots ,{\omega }_{{n}_{p}\left({\mathcal{A}}_{{n}_{\mathcal{A}}}\right)}\right)}^{{{\prime}}},\,\,\ddot{{\varvec{\upsilon}}}={\left({\varvec{\upsilon}},{{\varvec{\upsilon}}}_{\mathcal{A}}\right)}^{{\prime}}$, with ${\varvec{\upsilon}}={\left({\upsilon }_{1},\ldots ,{\upsilon }_{i},\ldots ,{\upsilon }_{{n}_{\mathcal{A}}}\right)}^{{\prime}}$and ${{\varvec{\upsilon}}}_{\mathcal{A}}={\left({\upsilon }_{1\left({\mathcal{A}}_{1}\right)},\ldots ,{\upsilon }_{g\left({\mathcal{A}}_{i}\right)},\ldots ,{\upsilon }_{{n}_{p}\left({\mathcal{A}}_{{n}_{\mathcal{A}}}\right)}\right)}^{{{\prime}}}$, ${\ddot{{\varvec{\updelta}}}}_{{\varvec{t}}}={\left({{\varvec{\updelta}}}_{t},{{\varvec{\updelta}}}_{\mathcal{A}t}\right)}^{{\prime}}$, with ${{\varvec{\updelta}}}_{t}={\left({\updelta }_{1t},\ldots ,{\updelta }_{it},\ldots,{\updelta }_{{n}_{\mathcal{A}}t}\right)}^{{{\prime}}},$ ${\phi }_{t}$ and ${\varsigma }_{t}$ defined as in Eq. (3), and ${{\varvec{\updelta}}}_{\mathcal{A}t}={\left({\updelta }_{1\left({\mathcal{A}}_{1}\right)t},\ldots ,{\updelta }_{g\left({\mathcal{A}}_{i}\right)t},\ldots ,{\updelta }_{{n}_{p}\left({\mathcal{A}}_{{n}_{\mathcal{A}}}\right)t}\right)}^{{{\prime}}},\,\,{\ddot{{\varvec{\Phi}}}}_{t}={\left({\overline{{\varvec{\Phi}}} }_{t},{{\varvec{\Phi}}}_{t}\right)}^{{\prime}}$, with ${\overline{{\varvec{\Phi}}} }_{t}={\left({\overline{\Phi } }_{1t},\ldots ,{\overline{\Phi } }_{it},\ldots ,{\overline{\Phi } }_{{n}_{\mathcal{A}}t}\right)}^{{{\prime}}}$and ${{\varvec{\Phi}}}_{t}=({\Phi }_{1t},\ldots ,{\Phi }_{gt},\ldots ,{\Phi }_{{n}_{p}t})\boldsymbol{^{\prime}}$. Note that the above vectors are ((${n}_{\mathcal{A}}+{n}_{p})\times 1)$ for $t=1, \ldots ,T.$

The following observations apply. First, the basic components of a high-resolution spatiotemporal relative risk model are the covariates and/or the GF at cell level $\left(\Phi \left({{\varvec{s}}}_{g},t\right)\right)$. Either one or both are required for the estimation of the relative risk at cell level. Second, the interaction terms ${\delta }_{it}$ and ${\Phi }_{gt}$ in the non-separable models in Eqs. (3a) and (3b), respectively, have as covariance matrices the Kronecker products of the spatial and temporal covariance matrices (Blangiardo and Cameletti 2015; Fuentes et al. 2008). See Table 5 in Appendix 2 and Sect. 3.2 for further details. For alternative approaches to handling non-separable models, see among others Bakka et al. (2020), Gneiting (2002), and Sherman (2011). Third, the parameters $\left\{{\omega }_{i}, {\upsilon }_{i},{\delta }_{it}\right\}$ are estimated at area level. For the cell level, they are the corresponding area level parameters, implying that they do not vary among cells within a given area ${\mathcal{A}}_{i}$. Fourth, to control for misalignment, for each area ${\mathcal{A}}_{i}$, the model component ${\overline{\Phi } }_{it}$ and the area values of the risk factors ${\overline{{\rm x}} }_{k,it}$ are taken as the block averages of the cells within ${\mathcal{A}}_{i}$ for a given time point $t$, respectively (Banerjee et al. 2015). That is, for $i=1,\ldots ,{n}_{\mathcal{A}}$ and $t=1,\ldots ,T$, ${\overline{{\rm x}} }_{k,it}={\left|{\mathcal{A}}_{i}\right|}^{-1}\underset{{\mathcal{A}}_{i}}{\overset{}{\int }}{{\rm x}}_{k}\left(\mathbf{s},t\right){\rm d}{\varvec{s}}\,\,{\rm for}\,\,k=1,\ldots ,K$ and ${\overline{\Phi } }_{it}={\left|{\mathcal{A}}_{i}\right|}^{-1}\underset{{\mathcal{A}}_{i}}{\overset{}{\int }}\Phi \left({\varvec{s}},t\right){\rm d}{\varvec{s}}$ where $\left|{\mathcal{A}}_{i}\right|=\underset{{\mathcal{A}}_{i}}{\overset{}{\int }}1{\rm d}{\varvec{s}}$ denotes the size of ${\mathcal{A}}_{i}$. The simplest procedure to estimate ${\overline{{\rm x}} }_{k,it}$ is to approximate ${\left|{\mathcal{A}}_{i}\right|}^{-1}\underset{{\mathcal{A}}_{i}}{\overset{}{\int }}{\mathbf{x}}_{k}\left({\varvec{s}},t\right){\rm d}{\varvec{s}}$ for each time $t$ by taking the average of the values of the cell risk factor ${{\rm x}}_{k,g\left({\mathcal{A}}_{i}\right)t}$ in ${\mathcal{A}}_{i}$: ${\overline{{\rm x}} }_{k,it}\approx \frac{1}{{n}_{{\mathcal{A}}_{i}}}\sum_{{{\varvec{s}}}_{g}\in {\mathcal{A}}_{i}}{\mathbf{x}}_{k}\left({{\varvec{s}}}_{g},t\right)$ for $k=1, \ldots ,K$, $i=1,\ldots ,{n}_{\mathcal{A}},$ and $t=1,\ldots,T$, with ${n}_{{\mathcal{A}}_{i}}$ denoting the number of cells in ${\mathcal{A}}_{i}$ (Lawson et al. 2012; Utazi et al. 2019). Estimation of ${\overline{\Phi } }_{it}={\left|{\mathcal{A}}_{i}\right|}^{-1}\underset{{\mathcal{A}}_{i}}{\overset{}{\int }}{\varvec{\Phi}}\left({\varvec{s}},t\right){\rm d}{\varvec{s}}$ is discussed in Sect. 3.2.

Bayesian estimation of the FGG-GF model is initiated by defining the estimated parameter and hyperparameter vectors. Let $\boldsymbol{\ell}=\left({\beta }_{0},{\beta }_{1},\ldots ,{\beta }_{K},{{\varvec{\zeta}}}_{1},\ldots ,{{\varvec{\zeta}}}_{K},{\varvec{\upomega}},{\varvec{\upsilon}},{\varvec{\phi}},\boldsymbol{\varsigma },{\varvec{\updelta}},{\varvec{\Phi}}\right)$ and ${\varvec{\Psi}}=\left({\sigma }_{{\beta }_{0}}^{2},{\sigma }_{{\beta }_{1}}^{2},\ldots ,{\sigma }_{{\beta }_{K}}^{2},{\sigma }_{{\zeta }_{1}}^{2},\ldots ,{\sigma }_{{\zeta }_{K}}^{2},{\sigma }_{\upomega }^{2},{\sigma }_{\upsilon }^{2},{\sigma }_{\phi }^{2},{\sigma }_{\boldsymbol{\varsigma }}^{2},{\sigma }_{\updelta }^{2},{\sigma }_{\Phi }^{2},\rho ,{\lambda }_{1},{\lambda }_{2},r\right){^{\prime}}$ denote the parameter and hyperparameter vectors, respectively, of the FGG-GF model in Eq. (13). The joint posterior distribution of the FGG-GF model is:

14 $$p\left(\boldsymbol{\ell},{\varvec{\Psi}}|\mathbf{y}\right)\propto p\left(\mathbf{y}|\boldsymbol{\ell},{\varvec{\Psi}}\right)p\left(\boldsymbol{\ell}|{\varvec{\Psi}}\right)p\left({\varvec{\Psi}}\right)$$

where $p\left(.\right)$ denotes the probability density function. Below, we first discuss the likelihood function $p\left(\mathbf{y}|\boldsymbol{\ell},{\varvec{\Psi}}\right)$ and next the joint prior of the GF at cell level. Based on the assumption that $\mathbf{y}$ follows a Poisson distribution at area and cell levels (see Eq. (3a) and (3b)), the likelihood function $p\left(\mathbf{y}|\boldsymbol{\ell},{\varvec{\Psi}}\right)$ is given by:

15 $$\begin{aligned} p\left( {{\mathbf{y}}|{{\boldsymbol{\ell} }},{{\varvec{\Psi}}}} \right) & = \mathop \prod \limits_{t = 1}^{T} \frac{{\exp \left( {- {\mathbf{E}}_{t} \exp \left( {{{\varvec{\upeta}}}_{t} } \right)} \right)\left( {{\mathbf{E}}_{t} \exp \left( {{{\varvec{\upeta}}}_{t} } \right)} \right)^{{{\mathbf{y}}_{{\varvec{t}}} }} }}{{{\mathbf{y}}_{t} !}} \\ & = \exp \left( {\log \left( {\mathop \prod \limits_{t = 1}^{T} \frac{{\exp \left( {- {\mathbf{E}}_{t} \exp \left( {{{\varvec{\upeta}}}_{t} } \right)} \right)\left( {{\mathbf{E}}_{t} \exp \left( {{{\varvec{\upeta}}}_{t} } \right)} \right)^{{{\mathbf{y}}_{{\varvec{t}}} }} }}{{{\mathbf{y}}_{t} !}}} \right)} \right) \\ & = \exp \left( {\mathop \sum \limits_{t = 1}^{T} \log \left( {\frac{{\exp \left( {- {\mathbf{E}}_{t} \exp \left( {{{\varvec{\upeta}}}_{t} } \right)} \right)\left( {{\mathbf{E}}_{t} \exp \left( {{{\varvec{\upeta}}}_{t} } \right)} \right)^{{{\mathbf{y}}_{{\varvec{t}}} }} }}{{{\mathbf{y}}_{t} !}}} \right)} \right) \\ & = \exp \left( {\mathop \sum \limits_{t = 1}^{T} \left( {{\mathbf{y}}_{{\varvec{t}}} \left( {\log \left( {{\mathbf{E}}_{t} } \right) + {{\varvec{\upeta}}}_{t} } \right) - {\mathbf{E}}_{t} \exp \left( {{{\varvec{\upeta}}}_{t} } \right) - \log \left( {{\mathbf{y}}_{t} !} \right)} \right)} \right). \\ \end{aligned}$$

The joint prior of the GF at cell level is obtained as follows. Since the GF in Eq. (8) at cell level is assumed to follow an AR1 model, the joint prior distribution of ${\varvec{\Phi}}=({{\varvec{\Phi}}}_{1},\ldots,{{\varvec{\Phi}}}_{T})\mathbf{^{\prime}}$**,** i.e., $p\left({\varvec{\Phi}}|{\lambda }_{1},{\varvec{\Sigma}}\right)$, is (Godana et al. 2019):

16 $$p\left({{\varvec{\Phi}}}_{1},\ldots ,{{\varvec{\Phi}}}_{T}|{\lambda }_{1},{\varvec{\Sigma}}\right)=p\left({{\varvec{\Phi}}}_{T}|{{\varvec{\Phi}}}_{T-1},{{\varvec{\Phi}}}_{T-2},\ldots ,{{\varvec{\Phi}}}_{1},{\lambda }_{1},{\varvec{\Sigma}}\right)\times \ldots \times p\left({{\varvec{\Phi}}}_{2}|{{\varvec{\Phi}}}_{1},{\varvec{\Psi}}\right)\times p\left({{\varvec{\Phi}}}_{1}|{\lambda }_{1},{\varvec{\Sigma}}\right).$$

Because of the AR1 process, we have:

17 $$p\left({{\varvec{\Phi}}}_{T}|{{\varvec{\Phi}}}_{T-1},{{\varvec{\Phi}}}_{T-2},\ldots ,{{\varvec{\Phi}}}_{1},{\lambda }_{1},{\varvec{\Sigma}}\right)=p\left({{\varvec{\Phi}}}_{T}|{{\varvec{\Phi}}}_{T-1},{\lambda }_{1},{\varvec{\Sigma}}\right).$$

Thus, the joint distribution of the latent spatiotemporal Gaussian process ${\varvec{\Phi}}$ is:

18 $$p\left({{\varvec{\Phi}}}_{1},\ldots ,{{\varvec{\Phi}}}_{T}|{\lambda }_{1},{\varvec{\Sigma}}\right)=p\left({{\varvec{\Phi}}}_{1}|{\lambda }_{1},{\varvec{\Sigma}}\right)\prod_{t=2}^{T}p\left({{\varvec{\Phi}}}_{t}|{{\varvec{\Phi}}}_{t-1},{\lambda }_{1},{\varvec{\Sigma}}\right).$$

The joint distribution of the GF in Eq. (18) consists of two probability distributions: $p\left({{\varvec{\Phi}}}_{1}|{\lambda }_{1},{\varvec{\Sigma}}\right)$ and $p\left({{\varvec{\Phi}}}_{t}|{{\varvec{\Phi}}}_{t-1},{\lambda }_{1},{\varvec{\Sigma}}\right)$ for $t=2,\ldots ,T$. To obtain the joint distribution of ${\varvec{\Phi}}$, we need the joint distributions of $p\left({{\varvec{\Phi}}}_{1}|{\lambda }_{1},{\varvec{\Sigma}}\right)$ for $g=1,\ldots ,{n}_{p}$ and $t=1$ and $p\left({{\varvec{\Phi}}}_{t}|{{\varvec{\Phi}}}_{t-1},{\lambda }_{1},{\varvec{\Sigma}}\right)$ for $g=1,\ldots ,{n}_{p}$ and $t=2,\ldots .,T$. ${{\varvec{\Phi}}}_{1}$ is an AR1 stationary process, i.e., ${{\varvec{\Phi}}}_{1}|{\lambda }_{1},{\varvec{\Sigma}}\sim \mathcal{N}\left(0,\frac{{\varvec{\Sigma}}}{1-{\lambda }_{1}^{2}}\right).$ It is called the initial distribution for $g=1,\ldots ,{n}_{p}$ and reads as:

19 $$p\left({{\varvec{\Phi}}}_{1}|{\lambda }_{1},{\varvec{\Sigma}}\right)={\left(\frac{1}{\sqrt{2\pi }}\right)}^{{n}_{p}}\frac{1}{{\left|\frac{{\varvec{\Sigma}}}{1-{\lambda }_{1}^{2}}\right|}^{1/2}}{\rm exp}\left(-\frac{1}{2}{{\varvec{\Phi}}}_{1}^{{{\prime}}}{\left(\frac{{\varvec{\Sigma}}}{1-{\lambda }_{1}^{2}}\right)}^{-1}{{\varvec{\Phi}}}_{1}\right).$$

Because ${\varvec{\Sigma}}={\sigma }_{\Phi }^{2}\mathcal{R}$, with $\mathcal{R}$ defined in Eq. (10), we have:

20 $$\begin{aligned} p\left( {{{\varvec{\Phi}}}_{1} |\lambda_{1} ,{{\varvec{\Sigma}}}} \right) & = \left( {\frac{1}{{\sqrt {2\pi } }}} \right)^{{n_{p} }} \frac{1}{{\left| {\frac{{\sigma_{{\Phi }}^{2} }}{{1 - \lambda_{1}^{2} }}{\boldsymbol{\mathcal{R}}}} \right|^{1/2} }}\exp \left( {- \frac{1}{{2\sigma_{{\Phi }}^{2} }}{{\varvec{\Phi}}}_{1}^{{^{\prime}}} \left( {\frac{{\boldsymbol{\mathcal{R}}}}{{1 - \lambda_{1}^{2} }}} \right)^{- 1} {{\varvec{\Phi}}}_{1} } \right) \\ & = \left( {\frac{1}{{\sqrt {2\pi } }}} \right)^{{n_{p} }} \left( {\frac{{\sigma_{{\Phi }}^{2} }}{{1 - \lambda_{1}^{2} }}} \right)^{{- \frac{{n_{p} }}{2}}} \left| {\boldsymbol{\mathcal{R}}} \right|^{{- \frac{1}{2}}} \exp \left( {- \frac{{\left( {1 - \lambda_{1}^{2} } \right)}}{{2\sigma_{{\Phi }}^{2} }}{{\varvec{\Phi}}}_{1}^{{^{\prime}}} {\boldsymbol{\mathcal{R}}}^{- 1} {{\varvec{\Phi}}}_{1} } \right) \\ & \propto \exp \left( {- \frac{{\left( {1 - \lambda_{1}^{2} } \right)}}{{2\sigma_{{\Phi }}^{2} }}{{\varvec{\Phi}}}_{1}^{{^{\prime}}} {\boldsymbol{\mathcal{R}}}^{- 1} {{\varvec{\Phi}}}_{1} } \right). \\ \end{aligned}$$

The joint distribution $p\left({{\varvec{\Phi}}}_{t}|{{\varvec{\Phi}}}_{t-1},{\lambda }_{1},{\varvec{\Sigma}}\right)$ for $g=1,\ldots ,{n}_{p}$ and $t=2,\ldots ,T$ is given by:

21 $$\begin{aligned} \mathop \prod \limits_{t = 2}^{T} p\left( {{{\varvec{\Phi}}}_{t} |{{\varvec{\Phi}}}_{t - 1} ,\lambda_{1} ,{{\varvec{\Sigma}}}} \right) & = \mathop \prod \limits_{t = 2}^{T} \left( {\frac{1}{{\sqrt {2\pi } }}} \right)^{{n_{p} }} \frac{1}{{\left| {\sigma_{{\Phi }}^{2} {\boldsymbol{\mathcal{R}}}} \right|^{1/2} }}\exp \left( {- \frac{1}{2}\left( {\left( {{{\varvec{\Phi}}}_{{\varvec{t}}} - \lambda_{1} {{\varvec{\Phi}}}_{{{\varvec{t}} - 1}} } \right){^{\prime}}\left( {\sigma_{{\Phi }}^{2} {\boldsymbol{\mathcal{R}}}} \right)^{- 1} \left( {{{\varvec{\Phi}}}_{{\varvec{t}}} - \lambda_{1} {{\varvec{\Phi}}}_{{{\varvec{t}} - 1}} } \right)} \right)} \right) \\ & = \mathop \prod \limits_{t = 2}^{T} \left( {\sqrt {2\pi } } \right)^{{\frac{{- n_{p} }}{2}}} \left( {\sigma_{{\Phi }}^{2} } \right)^{{- \frac{{n_{p} }}{2}}} \left| {\boldsymbol{\mathcal{R}}} \right|^{- 1/2} \exp \left( {- \frac{1}{2}\left( {\left( {{{\varvec{\Phi}}}_{{\varvec{t}}} - \lambda_{1} {{\varvec{\Phi}}}_{{{\varvec{t}} - 1}} } \right){^{\prime}}\left( {\sigma_{{\Phi }}^{2} {\boldsymbol{\mathcal{R}}}} \right)^{- 1} \left( {{{\varvec{\Phi}}}_{{\varvec{t}}} - \lambda_{1} {{\varvec{\Phi}}}_{{{\varvec{t}} - 1}} } \right)} \right)} \right) \\ & = \left( {\sqrt {2\pi } } \right)^{{\frac{{- n_{p} \left( {T - 1} \right)}}{2}}} \left( {\sigma_{{\Phi }}^{2} } \right)^{{- \frac{{n_{p} \left( {T - 1} \right)}}{2}}} \left| {\boldsymbol{\mathcal{R}}} \right|^{{- \frac{{\left( {T - 1} \right)}}{2}}} \exp \left( {- \frac{1}{{2\sigma_{{\Phi }}^{2} }}\mathop \sum \limits_{t = 2}^{T} \left( {\left( {{{\varvec{\Phi}}}_{{\varvec{t}}} - \lambda_{1} {{\varvec{\Phi}}}_{{{\varvec{t}} - 1}} } \right){^{\prime}}{\boldsymbol{\mathcal{R}}}^{- 1} \left( {{{\varvec{\Phi}}}_{{\varvec{t}}} - \lambda_{1} {{\varvec{\Phi}}}_{{{\varvec{t}} - 1}} } \right)} \right)} \right) \\ & \propto \exp \left( {- \frac{1}{{2\sigma_{{\Phi }}^{2} }}\mathop \sum \limits_{t = 2}^{T} \left( {\left( {{{\varvec{\Phi}}}_{{\varvec{t}}} - \lambda_{1} {{\varvec{\Phi}}}_{{{\varvec{t}} - 1}} } \right){^{\prime}}{\boldsymbol{\mathcal{R}}}^{- 1} \left( {{{\varvec{\Phi}}}_{{\varvec{t}}} - \lambda_{1} {{\varvec{\Phi}}}_{{{\varvec{t}} - 1}} } \right)} \right)} \right). \\ \end{aligned}$$

Finally, the joint prior distribution for the AR1 process, denoted as $p\left({\varvec{\Phi}}|{\mathbf{Q}}_{{\varvec{\Phi}}}^{-1}\right)$, is given by multiplying Eqs. (20) and (21). It reads:

22 $$\begin{aligned} p\left( {{{\varvec{\Phi}}}|{\mathbf{Q}}_{{{\varvec{\Phi}}}}^{- 1} } \right) & \propto \exp \left( {- \frac{{\left( {1 - \lambda_{1}^{2} } \right)}}{{2\sigma_{{\Phi }}^{2} }}{{\varvec{\Phi}}}_{1}^{{^{\prime}}} {\boldsymbol{\mathcal{R}}}^{- 1} {{\varvec{\Phi}}}_{1} } \right) \times \exp \left( {- \frac{1}{{2\sigma_{{\Phi }}^{2} }}\mathop \sum \limits_{t = 2}^{T} \left( {\left( {{{\varvec{\Phi}}}_{{\varvec{t}}} - \lambda_{1} {{\varvec{\Phi}}}_{{{\varvec{t}} - 1}} } \right){^{\prime}}{\boldsymbol{\mathcal{R}}}^{- 1} \left( {{{\varvec{\Phi}}}_{{\varvec{t}}} - \lambda_{1} {{\varvec{\Phi}}}_{{{\varvec{t}} - 1}} } \right)} \right)} \right) \\ & \propto \exp \left( {- \frac{1}{2}\left( {\frac{1}{{\sigma_{{\Phi }}^{2} }}\left( {{{\varvec{\Phi}}}_{1}^{{^{\prime}}} \left( {1 - \lambda_{1}^{2} } \right){\boldsymbol{\mathcal{R}}}^{- 1} {{\varvec{\Phi}}}_{1} + \mathop \sum \limits_{t = 2}^{T} \left( {\left( {{{\varvec{\Phi}}}_{{\varvec{t}}} - \lambda_{1} {{\varvec{\Phi}}}_{{{\varvec{t}} - 1}} } \right){^{\prime}}{\boldsymbol{\mathcal{R}}}^{- 1} \left( {{{\varvec{\Phi}}}_{{\varvec{t}}} - \lambda_{1} {{\varvec{\Phi}}}_{{{\varvec{t}} - 1}} } \right)} \right)} \right)} \right)} \right) \\ & \propto \exp \left( {- \frac{1}{2}{{\varvec{\Phi}}}_{{\varvec{t}}} {^{\prime}}{\mathbf{Q}}_{{{\varvec{\Phi}}}} {{\varvec{\Phi}}}_{{\varvec{t}}} } \right) \\ & \propto {\mathcal{N}}\left( {0,{\mathbf{Q}}_{{{\varvec{\Phi}}}}^{- 1} } \right) \\ \end{aligned}$$

with ${\mathbf{Q}}_{{\varvec{\Phi}}}^{-1}={\varvec{\Sigma}}$ denoting the covariance matrix of the GF in Eq. (12).

The priors and joint priors of $\boldsymbol{\ell}=\left({\beta }_{0},{\beta }_{1},\ldots ,{\beta }_{K},{{\varvec{\zeta}}}_{1},\ldots ,{{\varvec{\zeta}}}_{K},{\varvec{\upomega}},{\varvec{\upsilon}},{\varvec{\phi}},\boldsymbol{\varsigma },{\varvec{\updelta}},{\varvec{\Phi}}\right)$ and the hyperpriors of ${\varvec{\Psi}}=\left({\sigma }_{{\beta }_{0}}^{2},{\sigma }_{{\beta }_{1}}^{2},\ldots ,{\sigma }_{{\beta }_{K}}^{2},{\sigma }_{{\zeta }_{1}}^{2},\ldots ,{\sigma }_{{\zeta }_{K}}^{2},{\sigma }_{\upomega }^{2},{\sigma }_{\upsilon }^{2},{\sigma }_{\phi }^{2},{\sigma }_{\boldsymbol{\varsigma }}^{2},{\sigma }_{\updelta }^{2},{\sigma }_{\Phi }^{2},\rho ,{\lambda }_{1},{\lambda }_{2},r\right){^{\prime}}$ are presented in Table 5 in Appendix 2.^12^ Details can be found in Jaya and Folmer (2021a) and the references therein. The prior distributions are assumed to be independent, implying that:

23 $$\begin{aligned} p\left( {\boldsymbol{\ell} |{{\varvec{\Psi}}}} \right) & = {\mathcal{N}}\left( {0,\sigma_{{\beta_{0} }}^{2} } \right) \times {\mathcal{N}}\left( {0;{\mathbf{Q}}_{{\varvec{\beta}}}^{- 1} } \right) \times \mathop \prod \limits_{k = 1}^{K} {\mathcal{N}}\left( {0;{\mathbf{Q}}_{{{\varvec{\zeta}}_{k} }}^{- 1} } \right) \times {\mathcal{N}}\left( {0,{\mathbf{Q}}_{{\upomega }}^{- 1} } \right) \times {\mathcal{N}}\left( {0,{\mathbf{Q}}_{\upsilon }^{- 1} } \right) \\ & \quad \times {\mathcal{N}}\left( {0,{\mathbf{Q}}_{\phi }^{- 1} } \right) \times {\mathcal{N}}\left( {0,{\mathbf{Q}}_{\varvec{\varsigma }}^{- 1} } \right) \times {\mathcal{N}}\left( {0,{\mathbf{Q}}_{{{\varvec{\updelta}}}}^{- 1} } \right) \times {\mathcal{N}}\left( {0,{\mathbf{Q}}_{{{\varvec{\Phi}}}}^{- 1} } \right). \\ \end{aligned}$$

The joint hyperparameter distribution is given by^13^:

$$\begin{aligned} p\left( {{\varvec{\Psi}}} \right) & = p\left( {\sigma_{{\beta_{0} }}^{2} } \right)p\left( {\sigma_{{\beta_{1} }}^{2} } \right) \ldots p\left( {\sigma_{{\beta_{K} }}^{2} } \right)p\left( {\sigma_{{\zeta_{1} }}^{2} } \right) \ldots p\left( {\sigma_{{\zeta_{K} }}^{2} } \right)p\left( {\sigma_{{\upomega }}^{2} } \right)p\left( {\sigma_{\upsilon }^{2} } \right)p\left( {\sigma_{\phi }^{2} } \right)p\left( {\sigma_{\varvec{\varsigma }}^{2} } \right)p\left( {\sigma_{{\Phi }}^{2} } \right)p\left( {\sigma_{{\updelta }}^{2} } \right)p\left( \rho \right)p\left( {\lambda_{1} } \right)p\left( {\lambda_{2} } \right)p\left( \kappa \right) \\ & = \mathop \prod \limits_{j = 1}^{{\dim \left( {{\varvec{\Psi}}} \right)}} p\left( {{\Psi }_{j} } \right) \quad {\text{for}} \quad j = 1, \ldots ,J. \\ \end{aligned}$$

Given the likelihood function, the joint prior distributions for the parameter vectors and the joint hyperparameter, the joint posterior distribution in Eq. (14) can be written as:

24 $$\begin{aligned} p\left( {{{\boldsymbol{\ell} }},{\varvec{\varPsi}}|{\varvec{y}}} \right) & \propto \exp \left( {\mathop \sum \limits_{t = 1}^{T} \left( {{\varvec{y}}_{t} \log \left( {{\varvec{E}}_{t} \exp \left( {{\varvec{\eta}}_{t} } \right)} \right) - {\varvec{E}}_{t} \exp \left( {{\varvec{\eta}}_{t} } \right)} \right)} \right) \\ & \quad \times \exp \left( {- \frac{1}{{2\sigma_{{\beta_{0} }}^{2} }}\beta_{0}^{2} } \right) \times \exp \left( {- \frac{1}{2}\varvec{\beta ^{\prime}}{\mathbf{Q}}_{{\varvec{\beta}}} {\varvec{\beta}}} \right) \times \exp \left( {- \frac{1}{2}\mathop \sum \limits_{k = 1}^{K} {\varvec{\zeta}}_{{\varvec{k}}}^{{^{\prime}}} {\mathbf{Q}}_{{\zeta_{{\varvec{k}}} }} {\varvec{\zeta}}_{k} } \right) \\ & \quad \times \exp \left( {- \frac{1}{2}\varvec{\omega^{\prime}}{\mathbf{Q}}_{{\varvec{\omega}}} {\varvec{\omega}}} \right) \times \exp \left( {- \frac{1}{2}\varvec{\upsilon^{\prime}}{\mathbf{Q}}_{\upsilon } {\varvec{\upsilon}}} \right) \times \exp \left( {- \frac{1}{2}\phi {^{\prime}}{\mathbf{Q}}_{\phi } \phi } \right) \times \exp \left( {- \frac{1}{2}\varvec{\varsigma ^{\prime}}{\mathbf{Q}}_{\varvec{\varsigma }} \varvec{\varsigma }} \right) \\ & \quad \times \exp \left( {- \frac{1}{2}{{\varvec{\Phi}}}{^{\prime}}{\mathbf{Q}}_{{{\varvec{\Phi}}}} {{\varvec{\Phi}}}} \right) \times \mathop \prod \limits_{j = 1}^{{\dim \left( {{\varvec{\Psi}}} \right)}} p\left( {{\Psi }_{j} } \right). \\ \end{aligned}$$

### The fusion area-cell spatiotemporal generalized geoadditive-Gaussian Markov random field model

A continuously indexed GF typically has a dense covariance matrix, such as ${\mathbf{Q}}_{{\varvec{\Phi}}}$ in Eq. (24), leading to complex, time-consuming numerical estimation challenges, commonly referred to as the “*big n*” problem. Lindgren et al. (2011) proposed to solve the big $n$ problem by substituting a sparse, discretely indexed Gaussian Markov Random Field (GMRF) for the continuously indexed GF.^14^ For a GMRF, the full conditional distribution for each component ${\upgamma }_{g,t}$ for $g=1,\ldots ,{n}_{p},$ only depends on a set of neighbors $N\left(g\right)$ as follows:

25 $$p\left({\upgamma }_{g,t}|{{\varvec{\upgamma}}}_{-g,t}\right)=p\left({\upgamma }_{g,t}|{{\varvec{\upgamma}}}_{N\left(g\right),t}\right) \quad {{\rm for}}\,g=1,\ldots ,{n}_{p}\,{{\rm and}}\,t=1,\ldots ,T$$

where $${{\varvec{\upgamma}}}_{-g,t}$$ denotes all the elements in $${\varvec{\upgamma}}$$ except $${\upgamma }_{g,t}$$, and $${{\varvec{\upgamma}}}_{N\left(g\right),t}$$ denotes all the elements of $${{\varvec{\upgamma}}}_{t}$$ in the neighborhood *N*($$g$$) of $${\upgamma }_{g,t}.$$ The vector of elements of $${{\varvec{\upgamma}}}_{t}$$ not in the neighborhood *N*($$g)$$ of $${\upgamma }_{g,t}$$ is denoted as $${{\varvec{\upgamma}}}_{-\left\{g,N\left(g\right)\right\},t}.\,\,{\upgamma }_{g,t}\,\,{\rm is}$$ conditionally independent of the elements of $${{\varvec{\upgamma}}}_{-\left\{g,N\left(g\right)\right\},t}$$. The conditional independence relationship is written as:

26 $${\upgamma }_{g,t}\perp {{\varvec{\upgamma}}}_{-\left\{g,N\left(g\right)\right\},t}|{{\varvec{\upgamma}}}_{N\left(g\right),t} \quad {{\rm for }}\,t=1,\ldots ,T\,{{\rm and }}\,g=1,\ldots ,{n}_{p}.$$

If Eq. (26) holds, the precision matrix $\mathbf{Q}={{\varvec{\Sigma}}}^{-1}$ of ${{\varvec{\upgamma}}}_{t}$ is sparse for each $t$. In other words, for a pair $g$ and $h$ with $g\ne h$ and $h\notin \left\{N(g)\right\}$, we have:

27 $${\upgamma }_{g,t}\perp {\upgamma }_{h,t}|{{\varvec{\upgamma}}}_{-\left(g,h\right),t}\iff \mathbf{Q}(g,h)=0 \quad {{\rm for}}\, g=1,\ldots ,{n}_{p} \,{{\rm and }}\,t=1,\ldots ,T$$

implying that the nonzero pattern in the precision matrix $\mathbf{Q}$ is given by the neighborhood structure. Conversely,

28 $$\mathbf{Q}\left(g,h\right)\ne 0,\,{{\rm if }}\,h\in \left\{N\left(g\right)\right\}.$$

Lindgren et al. (2011) proposed the Linear Stochastic Partial Differential Equation (LSPDE)^15^ approach based on a mesh of the study area, to transform a dense Matérn covariance matrix of a GF, such as in Eq. (11), into a sparse Matérn precision matrix of a GMRF (see Appendix 1). Specifically, for $t=1, \ldots , T,$ the GF ${\upgamma }_{g,t}$ in Eq. (12) with Matérn covariance function ${\varvec{\Sigma}}={\sigma }_{\Phi }^{2}\mathcal{R}$, is transformed into a GMRF, ${\stackrel{\sim }{{\varvec{\upgamma}}}}_{t}\left({\varvec{s}}\right)\sim \mathcal{N}\left(0,{\stackrel{\sim }{\mathbf{Q}}}_{s}^{-1}\right),$ with sparse spatial precision matrix ${\stackrel{\sim }{\mathbf{Q}}}_{{\varvec{s}}}$ defined in Eq. (47). Consequently, for $t=1,\ldots ,T$, the joint latent spatiotemporal GF ${{\varvec{\Phi}}}_{t}={\left({\Phi }_{1t},\ldots ,{\Phi }_{{n}_{p}t}\right)}^{{{\prime}}}$ at cell level in Eq. (12) is transformed into a GMRF ${\stackrel{\sim }{{\varvec{\Phi}}}}_{t}$ as:

29 $${\stackrel{\sim }{{\varvec{\Phi}}}}_{t}={\lambda }_{1}{\stackrel{\sim }{{\varvec{\Phi}}}}_{t-1}+{\stackrel{\sim }{{\varvec{\upgamma}}}}_{t}\,\,{\rm and }\,\,{\stackrel{\sim }{{\varvec{\upgamma}}}}_{t}({\varvec{s}})\sim \mathcal{N}\left(0,{\stackrel{\sim }{\mathbf{Q}}}_{s}^{-1}\right)$$

with the initial value distributed as: ${\stackrel{\sim }{{\varvec{\Phi}}}}_{1}\sim \mathcal{N}\left(0,{\stackrel{\sim }{\mathbf{Q}}}_{s}^{-1}/\left(1-{\lambda }_{1}^{2}\right)\right)$. The joint distribution of the $T\times L$-dimensional cell level GMRF $\stackrel{\sim }{{\varvec{\Phi}}}=\left({\stackrel{\sim }{{\varvec{\Phi}}}}_{1}^{{{\prime}}},\ldots ,{\stackrel{\sim }{{\varvec{\Phi}}}}_{T}^{{{\prime}}}\right)\boldsymbol{^{\prime}}$ is:

30 $$\stackrel{\sim }{{\varvec{\Phi}}}\sim \mathcal{N}\left(0,{\stackrel{\sim }{\mathbf{Q}}}_{\stackrel{\sim }{{\varvec{\Phi}}}}^{-1}\right)$$

with precision matrix ${\mathbf{Q}}_{\stackrel{\sim }{{\varvec{\Phi}}}}={\mathbf{Q}}_{{\varvec{T}}}\otimes {\stackrel{\sim }{\mathbf{Q}}}_{s}$, i.e., the Kronecker product of the autoregressive temporal covariance matrix (${\mathbf{Q}}_{{\varvec{T}}})$ (see Table 5 in Appendix 2) and the Matérn spatial covariance matrix (${\stackrel{\sim }{\mathbf{Q}}}_{s})$, respectively.

To facilitate the estimation of ${\ddot{{\varvec{\Phi}}}}_{t}={\left({\overline{{\varvec{\Phi}}} }_{t},{{\varvec{\Phi}}}_{t}\right)}^{{\prime}}$, with ${\overline{{\varvec{\Phi}}} }_{t}={\left({\overline{\Phi } }_{1t},\ldots ,{\overline{\Phi } }_{it},\ldots ,{\overline{\Phi } }_{{n}_{\mathcal{A}}t}\right)}^{{{\prime}}}$ and ${{\varvec{\Phi}}}_{t}=({\Phi }_{1t},\ldots ,{\Phi }_{gt},\ldots ,{\Phi }_{{n}_{p}t})\boldsymbol{^{\prime}}$ ${{\varvec{\Phi}}}_{t}=({\Phi }_{1t},\ldots ,{\Phi }_{gt},\ldots ,{\Phi }_{{n}_{p}t})\boldsymbol{^{\prime}}$ for $t=1,\ldots ,T$, (Eq. (13)) as a GMRF $\stackrel{\sim }{{\varvec{\Phi}}}$, we introduce the $\left(\left({n}_{\mathcal{A}}+{n}_{p}\right)\times L\right)$—dimensional partitioned or block matrix $$\mathbf{H}={\left[\begin{array}{cc}{\mathbf{H}}_{1}& {\mathbf{H}}_{2}\end{array}\right]}^{-1}$$ that maps the GMFRs associated with the $L$ triangulation nodes^16^ to the ${n}_{\mathcal{A}}$ areas and ${n}_{p}$ cells, respectively. The elements of ${\mathbf{H}}_{1}$ correspond to the block average ${\left|{\mathcal{A}}_{i}\right|}^{-1}\underset{{\mathcal{A}}_{i}}{\overset{}{\int }}\Phi \left({\varvec{s}},t\right){\rm d}{\varvec{s}}$ for $i=1,\ldots ,{n}_{\mathcal{A}}, t=1,\ldots ,T\,{{\rm and}}\,{\varvec{s}}={({\varvec{s}}}_{1({\mathcal{A}}_{1})},\ldots ,{{\varvec{s}}}_{g\left({\mathcal{A}}_{i}\right)},\ldots , {{\varvec{s}}}_{{n}_{p}({\mathcal{A}}_{{n}_{\mathcal{A}}})}){^{\prime}}$. That is, ${\mathbf{H}}_{1}$ is the $({n}_{\mathcal{A}}\times L)$ sparse matrix with ${{\varvec{H}}}_{1}(i,l)=1/{V}_{i}$ if vertex $l$ is in area ${\mathcal{A}}_{i}$ and zero otherwise and ${V}_{i}$ is the number of vertices in the area ${\mathcal{A}}_{i}$. Hence, matrix ${{\varvec{H}}}_{1}$ reads:31

Consequently, ${\overline{\Phi } }_{it}\approx \sum_{l=1}^{L}{{\varvec{H}}}_{1}\left(i,l\right){\stackrel{\sim }{\Phi }}_{t,l}{\rm for} t=1,\ldots ,T$ with ${\stackrel{\sim }{\Phi }}_{t,l}{\rm the} (t,l)$ th element of $\stackrel{\sim }{{\varvec{\Phi}}}$.

${{\varvec{H}}}_{2}$ transforms the ($T\times L$) elements of $\stackrel{\sim }{{\varvec{\Phi}}}$ into $({n}_{p}\times T)$ elements of ${\varvec{\Phi}}$ with the value of the $(g,t)$ th element ${\varvec{\Phi}}$ corresponding to the value of $(t,l)$ th of $\stackrel{\sim }{{\varvec{\Phi}}}$. That is, ${{\varvec{H}}}_{2}$ is an $({n}_{p}\times L)$ sparse matrix with ${{\varvec{H}}}_{2}(g,l)=1$ if the vertex *l* is at location ${{\varvec{s}}}_{g}$ and zero elsewhere such that for the *g*th cell ${\Phi }_{g,t}\approx \sum_{l=1}^{L}{{\varvec{H}}}_{2}(g,l){\stackrel{\sim }{\Phi }}_{t,l}$ for $i=1,\ldots ,{n}_{p}\,{{\rm and}}\,t=1,\ldots ,T$. Hence, matrix ${{\varvec{H}}}_{2}$ reads:32

Given the partitioned matrix ${\varvec{H}}$, the FGG-GF in Eq. (13) can be written as:

33 $${{\varvec{\eta}}}_{t}={\beta }_{0}{1}_{({n}_{\mathcal{A}}+{n}_{p})}+\sum\limits_{{\varvec{k}}=1}^{{\varvec{K}}}\left({\beta }_{k}+{\zeta }_{k,t}\right){{\varvec{z}}}_{k,t}+\ddot{{\varvec{\upomega}}}+\ddot{{\varvec{\upsilon}}}+{\phi }_{t}{1}_{({n}_{\mathcal{A}}+{n}_{p})}+{\varsigma }_{t}{1}_{({n}_{\mathcal{A}}+{n}_{p})}+{\ddot{{\varvec{\updelta}}}}_{t}+\mathbf{H}{\stackrel{\sim }{{\varvec{\Phi}}}}_{t} \quad {{\rm for}}\,t=1,\ldots ,T.$$

Because the FGG-GMRF model in Eq. (33) belongs to the class of the latent Gaussian models, it can be estimated using INLA-LSPDE (Cameletti et al. 2013; Gómez-Rubio et al. 2021). Predictions of the relative risk ${\varvec{\theta}}$ for the cells of the triangulated domain can be obtained via the posterior conditional distribution of $\stackrel{\sim }{{\varvec{\Phi}}}$, given ${\mathbf{Q}}_{\stackrel{\sim }{{\varvec{\Phi}}}}$ for all the $L$ vertices and the posterior distributions of the parameter and hyperparameters in Eq. (24). The FGG-GMRF model setup in Eq. (33) implies that INLA generates predictions for the target cells during model-fitting.

## Application: relative dengue risk at subdistrict level in Bandung, 2012–2018

Bandung city is divided into 30 districts and 151 subdistricts. The districts are third level administrative units within a province, and the subdistricts are fourth level administrative units. Every district in Bandung city consists of a minimum of four subdistricts. While the number of dengue incidences in Bandung city is reported at district level, for efficient and effective prevention and control, figures at the subdistrict scale are needed. In Sect. 4.1, we discuss and explore the data; in Sect. 4.2, we estimate the FGG-GMRF model; and in Sect. 4.3, we use the model to predict the relative dengue risk at subdistrict level.

### Data and exploratory data analysis

**Fig. 1:**
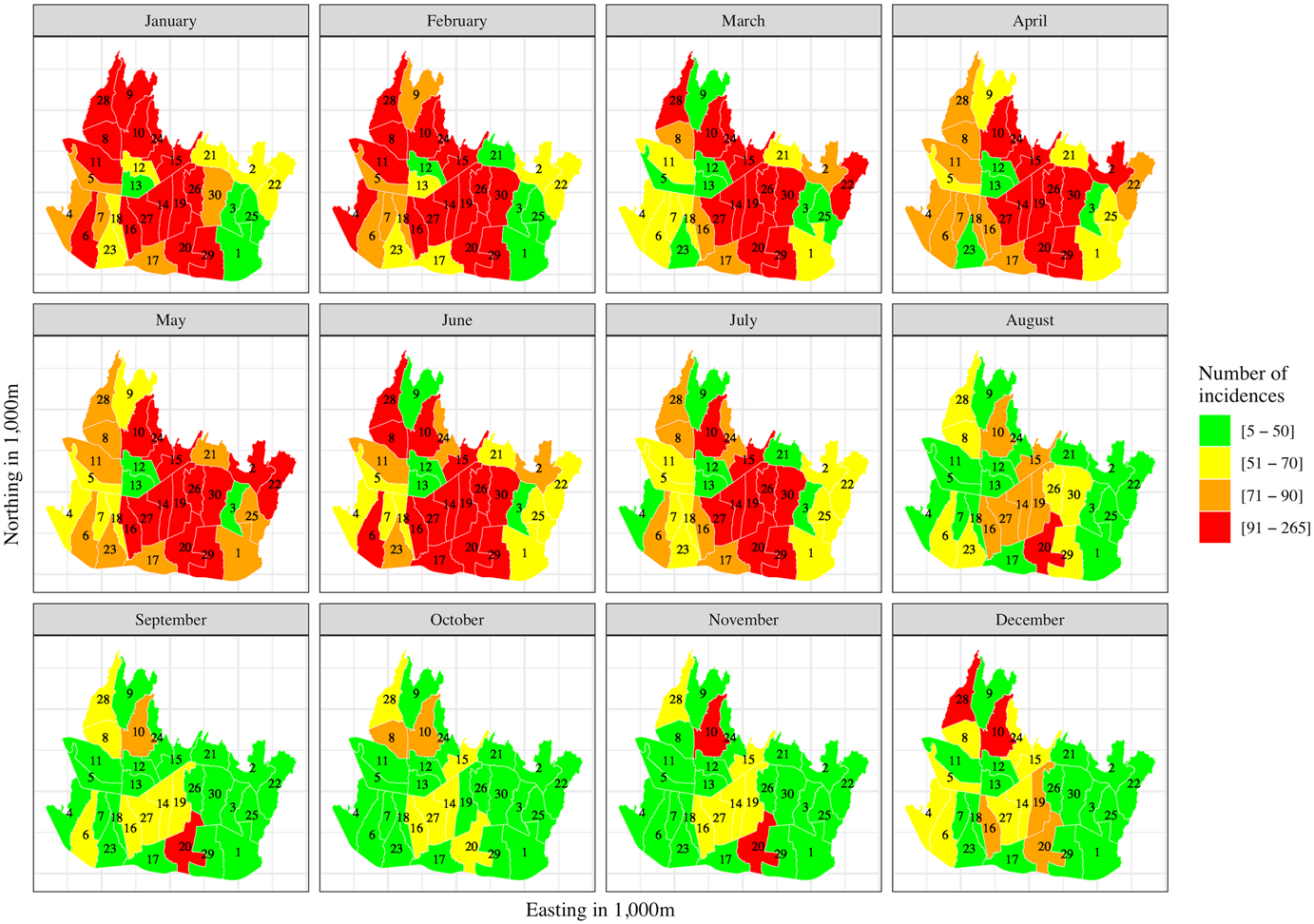
Monthly dengue incidences at district level (in 1,000), Bandung City, Indonesia, 2012–2018 (1–30: district codes, see Online Resource 1)

The data were obtained from existing databases. Annual observations at district level on the population at risk (see Online Resource 1) and monthly dengue incidence at district level (see Fig. 1) were obtained from the Bandung Central Statistical Bureau﻿ (2012, 2013, 2014, 2015, 2016, 2017, 2018) and the Bandung Health Department (2013, 2014, 2015, 2016, 2017, 2018, 2019), respectively. From January 1, 2012, until December 31, 2018, a total of 26,095 dengue incidences (1,030 per 100,000 inhabitants) were reported. The monthly incidence pattern is highly similar from year to year and is taken as constant. Particularly, the mean Pearson correlation coefficient of monthly dengue incidence for the years 2012–2018 is approximately 0.70. In addition, there were no major shifts across the years in the annual cycle. Hence, as in Jaya and Folmer (2021a), we only consider the monthly cycle for the 30 districts.^17^ Figure 1 shows a high number of incidences from January to July, followed by a sharp drop in July and a low level of incidences for the remainder of the year. The monthly incidences range from 5 to 265 (0.197–10.461 per 100,000 inhabitants, respectively).

**Fig. 2:**
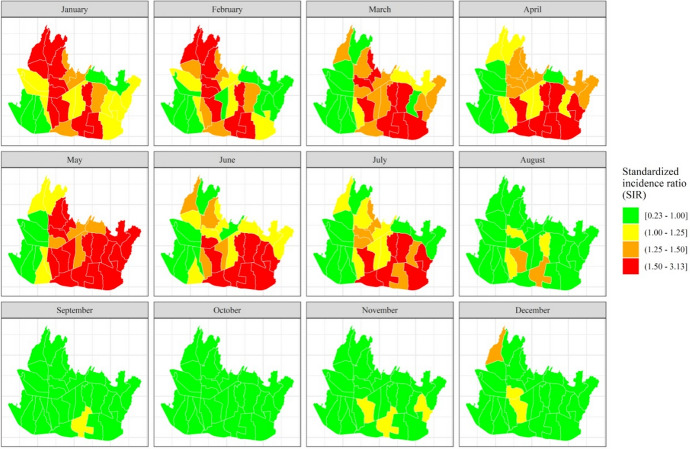
Monthly dengue standardized incidence ratio (SIR) at district level, Bandung City, Indonesia, 2012–2018

We derived the crude risk rate (i.e., the standardized incidence ratio, SIR) as the ratio of the observed to the expected number of incidences (see Eq. (2)). Figure 2 presents the monthly dengue SIR per district for the period 2012–2018. It ranges from 0.229 to 3.132. Most districts, primarily those in northern and southern Bandung, have a SIR greater than one from January to July. The districts with the highest SIR are Buah Batu (${\rm id}=20$), Lengkong (${\rm id}=27$) and Rancasari (${\rm id}=29$).

As observed by Ebi and Nealon (2016), Jaya and Folmer (2021a), and Zellweger et al. (2017), among others, socioeconomic and environmental conditions are the main factors influencing dengue disease risk over space and time. However, for Bandung, socioeconomic risk variables such as income, education, occupation and living conditions are unavailable for districts and cells. These factors are accounted for by the random effects, thus controlling for omitted variable bias (Jaya and Folmer 2020, 2021a, b). By contrast, the monthly averages of the weather risk variables of precipitation (mm), temperature (°C), sunshine duration (kJ/m^2^day) and water vapor pressure (kPa) are available at cell level from the WorldClim2.0 database (Fick and Hijmans 2017), obtained from 19 weather stations surrounding Bandung in West Java for 1970–2000. We selected cells of resolution 1 km^2^. Accordingly, Bandung city was divided into 179 cells. Table 1 presents the monthly average weather variables for the period 1970–2000.

**Table 1:** Descriptive statistics for the monthly averages of the weather variables^a^

Description	Mean	SD	Min	Max
Precipitation (mm)	189.024	89.030	52.000	328.000
Average temperature (°C)	22.632	0.896	19.300	24.000
Solar radiation (kJ/m^2^day)	16,938.484	1,317.538	15,041.000	19,924.000
Water vapor pressure (kPa)	2.095	0.140	1.670	2.300

^a^WorldClim (2020) Global climate and weather data, version 2.1. WorldClim: https://www.worldclim.org/. Accessed May 2020

**Fig. 3:**
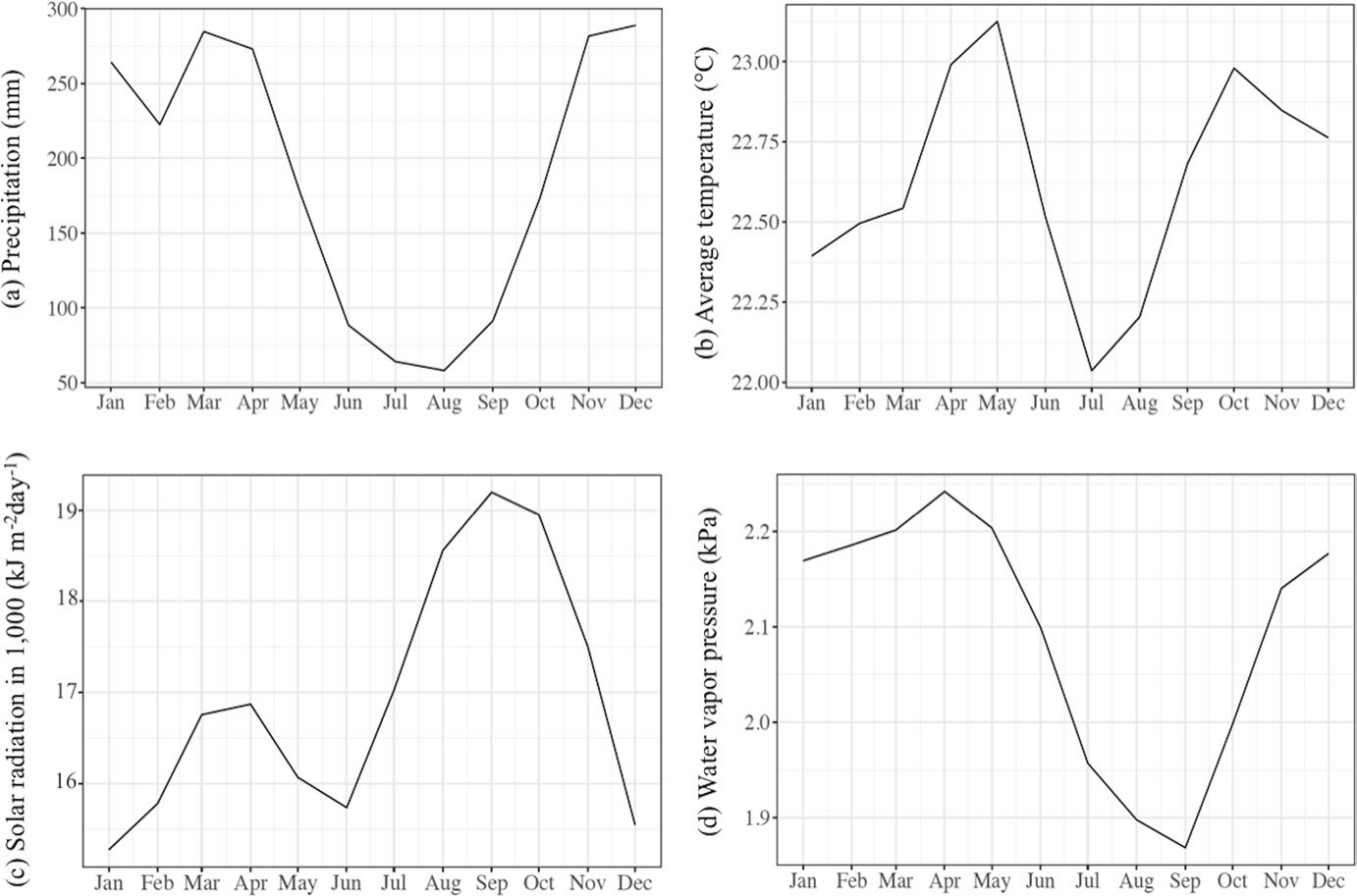
Monthly variation of the mean annual weather variables **a** precipitation (mm), **b** temperature (°C), **c** solar radiation in 1000 (kJ/m^2^day), and **d** water vapor pressure (kPa)

**Fig. 4:**
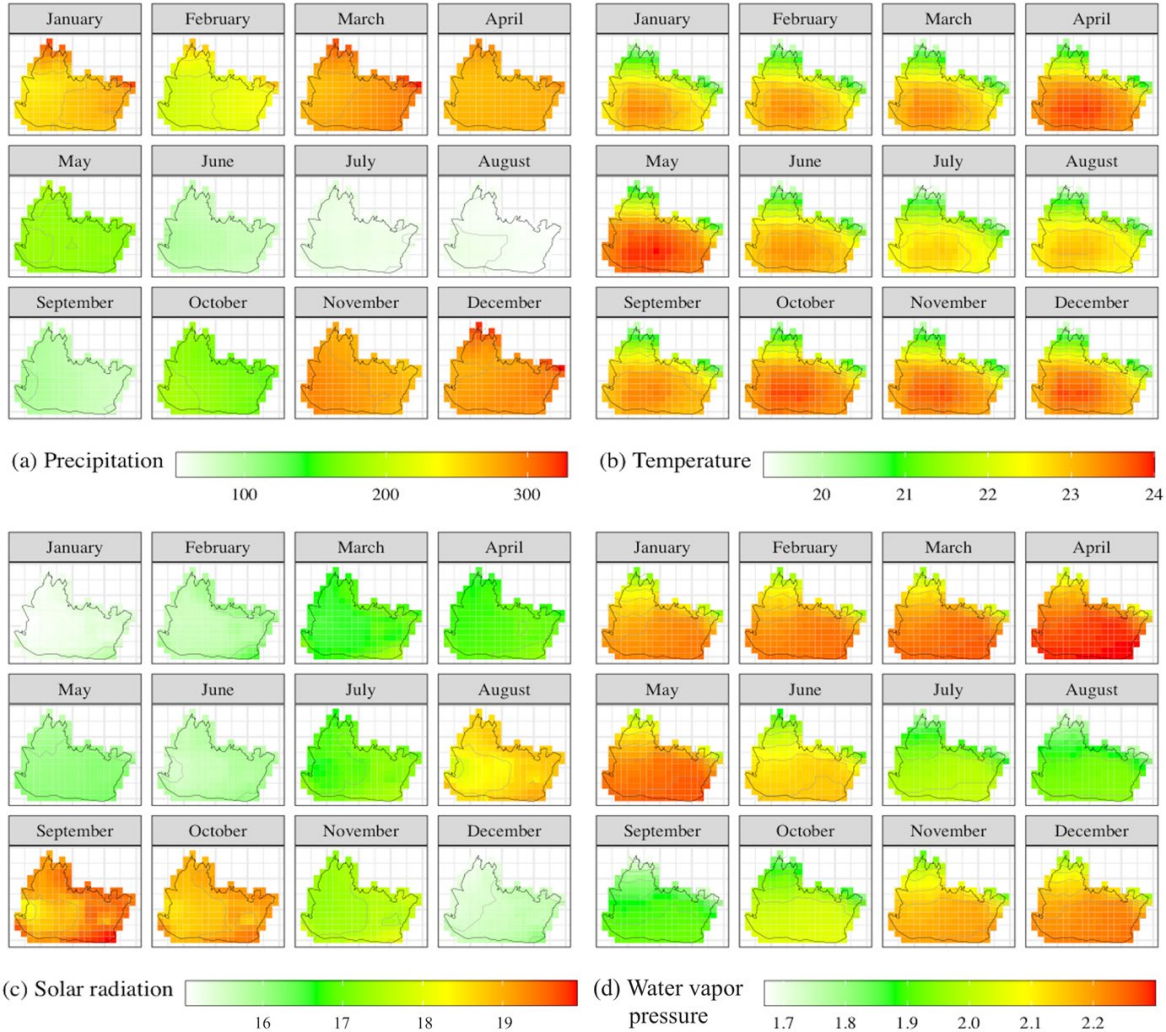
The spatiotemporal variation of the weather variables: **a** precipitation (mm), **b** temperature (°C), **c** solar radiation in 1000 (kJ/m^2^day), and **d** water vapor pressure (kPa)

Figure 3 shows that precipitation and water vapor pressure are relatively high in the period November–April, temperature is relatively high in the period April–June and in October, and solar radiation is relatively high in the period August–November. Figure 4 indicates that the average temperature varies strongly over space. The minimum average temperature occurs in the northern districts, which are mountainous areas at approximately 800 m above sea level. In addition, they are densely covered with forests and have relatively high precipitation. The central districts, where the governmental facilities and businesses are located, also have high precipitation in the period November to April. Moreover, they have higher temperatures than northern Bandung because of differences in forest density and elevation. They also have high population density, high mobility and high air pollution (Jaya and Folmer 2020).

In preparation for estimating the FGG-GMRF model in Eq. (33), we calculated the variance inflation factor (VIF) of the weather variables to check multicollinearity. Table 2 shows that the maximum VIF is 9.312 (for water vapor pressure) which is below the critical (rule of thumb) value of 10, indicating that the correlation among variables is unlikely to affect estimation (Montgomery et al. 2012).

**Table 2:** Variance inflation factors (VIF) for precipitation, average temperature, solar radiation and water vapor pressure

No	Variable	VIF
1	Precipitation (mm)	3.074
2	Average temperature (°C)	3.234
3	Solar radiation (kJ m^−2^ day^−1^)	3.347
4	Water vapor pressure (kPa)	9.312

VIF < 10 indicates that there is no serious multicollinearity

### The estimated generalized Geoadditive-Gaussian Markov Random Field model and prediction

The first step in estimating the FGG-GMRF model given by Eq. (33) is the construction of a triangle mesh of the study area for the application of the Finite Element Method (FEM) and LSPDE approach.

As described in Appendix 1, the accuracy of the FEM calculations and the precision of the forecasts, is a function of the number of vertices in the mesh (edge length). Blangiardo and Cameletti (2015) and Utazi et al. (2019) recommended varying the edge length between the minimum distance and approximately 5–8% of the maximum distance of any two cells (18,681 m). Hence, we considered $G=\left\{966, 831, 717, 616, 548, 501\right\}$ vertices, corresponding to edge lengths varying from 1000 to 1500 m, with a difference^18^ of 100 m (see Online Resource 1). The data and R code are available in Online Resource 2.

Before turning to the estimations of the spatiotemporal FGG-GMRF model, we make the following observations. First, as explained in Sect. 3, the covariates and/or the state process ${\varvec{\Phi}}$ at cell level are required for high-resolution spatiotemporal prediction using the FGG-GMRF model. Hence, either the covariates or ${\varvec{\Phi}}$, or both, are included in the selected model. Second, for every model, we considered Poisson and Negative Binomial model specifications for the number of incidences, a random walk of order one (RW1) and two (RW2) for the time-varying coefficients, structured and unstructured spatial and temporal random effects and their interaction, and six different edge lengths. Third, the best model was selected using the deviance information criterion (DIC), the Watanabe–Akaike information criterion (WAIC) and the marginal predictive likelihood (MPL). As a rule of thumb, the best model is the one with the smallest DIC and WAIC, and the largest MPL. Fourth, we started the estimation with the simplest models with covariates only (M1), and then, we proceeded to the model specifications with covariates and four types of interaction (see Table 5 in Appendix 2) at area and cell levels (M2) and, finally, we estimated the full models with covariates, four types of interaction at area and cell levels, and spatially and temporally structured and unstructured main effects (M3).^19^ Finally, due to the large number of outcomes, we only present the estimates for interaction Type IV (spatially structured $\otimes$ temporally structured) which, as in Jaya and Folmer (2020), performed best among the models with spatiotemporal interaction.^20^

The estimations are presented in Table 6 (in Appendix 3). The table shows that the models^21^ with covariates only (M1) have the worst fit and predictive performance among the three classes of models. They have the highest DICs, WAICs and smallest MPLs. Given their relatively poor fit and predictive performance, the M1 models were not considered further in the selection procedure. Introduction of the interaction effect type IV (M2) yielded substantially better predictive performance. The full models with covariates, interaction at area and cell levels and spatially and temporally structured and unstructured main effects (M3) had fit and predictive performance similar to the M2 models. Hence, the main spatially and temporally structured and unstructured effects did not improve the model fit and prediction performance, which is consistent with Jaya and Folmer (2020). Based on these observations, and because the M2 models have a simpler structure, we selected the class of M2 models.

Next, we turned to the selection of the best model from the 24 models M2.1.1.1–M2.2.2.6. First, we considered the edge length, finding that the models M2.1.1.1–M2.2.2.6. have similar DIC, WAIC and MPL values. Based on this observation, we selected the edge length of 1500 m for reasons of computational time. Online Resource 1 presents the mesh. Second, among the M2 models with edge length 1500 m, the Poisson model had slightly lower DIC and WAIC and slightly higher MPL than the Negative Binomial model. In addition, the models with temporal trends RW1 and RW2 had similar DIC, WAIC and MPL values. Based on these considerations, we selected model M2 with Poisson distribution, RW1 time-varying effect, and edge length of 1500 m (denoted as model M2.1.1.6 below).

**Fig. 5:**
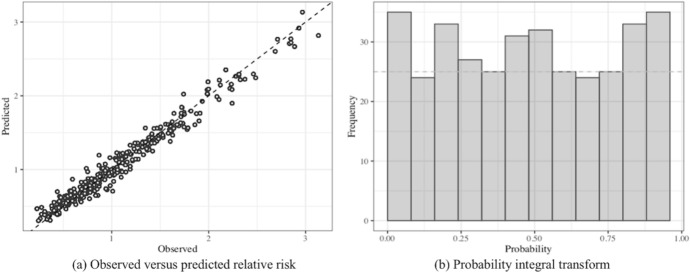
**a** Scatterplot for model M2.1.1.6 of the predicted versus the observed dengue relative risk and **b** histogram of the probability integral transform (PIT)

Figure 5a shows that for model M2.1.1.6, the observed and predicted dengue relative risks are strongly correlated $({\rm Pearson correlation coefficient} = 0.986)$, indicating that the model fits the data well. Figure 5b shows that the PIT histogram is close to the Uniform distribution, also indicating that model M2.1.1.6 fits the data well.

**Fig. 6:**
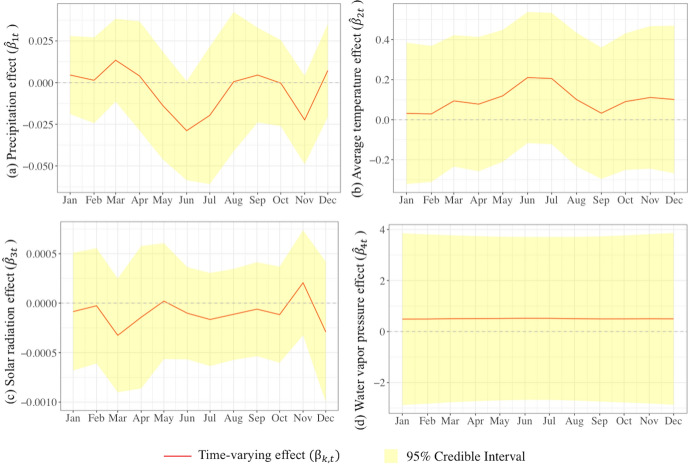
Time-varying effect (${\widehat{\beta }}_{k,t})$ of **a** precipitation (mm), **b** average temperature (°C), **c** solar radiation (kJ m^−2^ day^−1^) and **d** water vapor pressure (kPa)

**Fig. 7:**
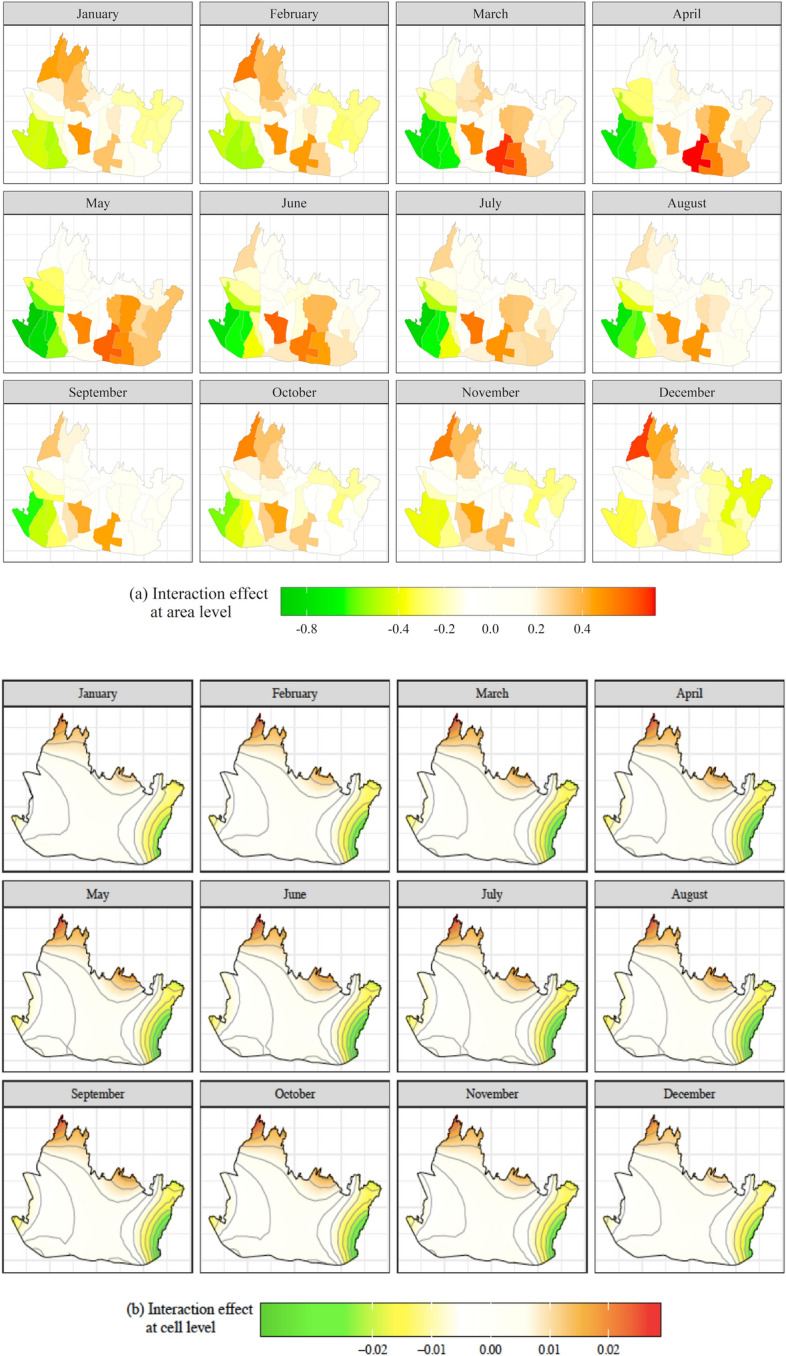
Monthly posterior means of **a** the area level interaction effect and **b** the cell level interaction effect, January–December

Table 3 summarizes various components of model M2.1.1.6 which are subsequently used to calculate the posterior means of the monthly relative risk at district and subdistrict levels. Before doing so, we discuss the components separately. To this end, we also make use of Figs. 6 and 7. Before going into detail, we make the following remarks. First, as observed in Sect. 2, p.8, the posterior means of the time-varying coefficients ${\beta }_{k,t}={\beta }_{k}+{\zeta }_{k,t}$ for $k=1,\ldots ,K\,{{\rm and}}\,t=1,\ldots ,T$, which are presented in Fig. 6, consist of the fixed effect plus the temporal random effect. The minimum and maximum posterior means of the temporal random effects present the largest negative and largest positive differences of the temporal random effects relative to the global effects. Secondly, the contributions of the weather variables in explaining the spatiotemporal variation of the dengue risk are conveniently summarized by the posterior means of their hyperparameter variances and their percentage contributions as fractions of the total variance (the last column of Table 3). In a similar vein, the posterior means and fractions of the hyperparameter variance of the random components present their variability and strength in explaining the relative risk across space and time.

**Table 3:** The posterior means and 95% credible intervals of the fixed effects, the minimum and maximum posterior means and 95% credible intervals of the temporal random effects and the posterior means and 95% credible intervals of the hyperparameters of the FGG-GMRF model M2.1.1.6

Parameter	Fixed effects /Global effects (${\beta }_{k})$		Random effects			
			Temporal random effects (${\zeta }_{k,t})$		Posterior means of the hyperparameters (95% credible interval)	Fraction of the total variance (%)
	Posterior means of the regression coefficients (95% credible interval)^a^	Percentage change (%)^b^	Minimum posterior means (95% credible interval)	Maximum posterior means (95% credible interval)		
Risk factors						
Precipitation (${x}_{1}$)	− 0.0041 (− 0.0146; 0.0064)	− 0.41	− 0.0248 (− 0.0440; − 0.0056)	0.0176 ( 0.0032; 0.0319)	0.019 (0.0191; 0.0191)	8.17
Temperature (${x}_{2}$)	0.1002 (− 0.1222; 0.3225)	10.54	− 0.0713 (− 0.1890; 0.0462)	0.1104 ( 0.0054; 0.2153)	0.015 (0.0145; 0.0145)	6.20
Solar radiation (${x}_{3}$)	− 0.0001 (− 0.0004; 0.0002)	− 0.01	− 0.0002 (− 0.0005; 0.0000)	0.0003 ( 0.0001; 0.0005)	0.023 (0.0231; 0.0231)	9.88
Water vapor pressure (${x}_{4}$)	0.5033 (− 2.4868; 3.4908)	65.42	− 0.0137 (− 0.3904; 0.3627)	0.0161 (− 0.1877; 0.2197)	0.018 (0.0178; 0.0178)	7.62
Interaction effect at area level (${\varvec{\updelta}}$)						
Variance of interaction effect (${\sigma }_{\updelta }^{2}$)					0.102 (0.1018; 0.1018)	43.47
Leroux CAR spatial autoregressive coefficient ($\rho$)					0.815 (0.8147; 0.8147)	
Temporal autoregressive coefficient (${\lambda }_{2}$)					0.962 (0.9619; 0.9619)	
Interaction effect at cell level (${\varvec{\Phi}}$)						
Variance of Interaction effect at cell level (${\sigma }_{\Phi }^{2}$)					0.058 (0.0578; 0.0578)	24.68
Spatial range ($r$) in km					13.597 (13.597; 13.598)	
Temporal autoregressive coefficient at cell level (${\lambda }_{1})$					0.749 (0.7485; 0.7485)	

^a^The 95% credible intervals are obtained from the posterior quantiles, i.e., $q(0.025)$ and $q(0.975)$. Using INLA, the posterior marginal distributions of the parameters approximately follow a normal distribution due to the Laplace approximation (Blangiardo and Cameletti 2015)

^b^The exact percentage change to ${\theta }_{it}$ for a one-unit change in a risk factor with all the other variables in the model held constant, is $100({e}^{{\beta }_{k}}-1)$

We will begin the discussion of Table 3 with the global mean effects of the weather variables. We only consider the posterior means and disregard credible intervals. Before going into the detail, it is worth noting that an indirect relationship exists between the relative risk of dengue and weather variables via the development and survival of the dengue virus and its vector (Jaya and Folmer 2021a).

Precipitation in general has a negative impact, with a posterior global (overall) mean of $-$ 0.0041, which is consistent with Jaya and Folmer (2021a). For a one mm increase in the global mean, the dengue risk decreases by $\left({\rm exp}\left(-0.0041\right)-1\right)100\%=-0. 41\%$. The explanation is that heavy rainfall disrupts the *Aedes-spp* mosquito’s reproductive cycle by washing away breeding sites (Abiodun et al. 2016; Benedum et al. 2018).

Temperature in general has a positive impact (0.1002), which is consistent with Hurtado-Díaz et al. (2007) and Jaya and Folmer (2021a). The relative risk increases by $\left({\rm exp}\left(0.1002\right)-1\right)100\%=10.54\%$ for an increase of global mean temperature by 1^0^C. The explanation is that higher temperatures offer good conditions for mosquito development, particularly feeding (Hales et al. 2002; Lambrechts et al. 2012).

Solar radiation has a negative impact (− 0.0001), which is consistent with Ekasari et al. (2018), Jaya and Folmer (2021a) and Martínez-Bello et al. (2017b). An increase of 1 kJ m^−2^ day^−1^ of solar radiation decreases the relative dengue risk by $\left({\rm exp}\left(-0.0001\right)-1\right)100\%=-0.01\%$. As shown by Rasjid et al. (2019), strong solar radiation negatively influences the breeding and spread of *Aedes-spp* mosquitoes. A longer spell of solar radiation implies a shortened spell of dawn and dusk, during which the *Aedes-spp* mosquito preys on animals and humans, particularly 20 to 30 min after sunset (Ekasari et al. 2018; Jaya and Folmer 2021a).

Water vapor pressure in general has a negative impact, with a posterior global mean of 0.5033. An increase of the global mean by 1% increases the relative dengue risk by $\left({\rm exp}\left(0.5033\right)-1\right)100\%=65.42\%$ due to an increase in breeding ability (Bambrick et al. 2009).

For the temporal random effects of the weather variables, the greatest negative temporal random effect of precipitation occurred in June (-0.0248), while the greatest positive occurred in March (0.0176). For temperature, the largest negative temporal random effect was in February (-0.0713), while the largest positive was in June (0.1104). Solar radiation has a temporal random effect close to zero, as indicated by its minimum and maximum posterior means of -0.0002 and 0.0003, respectively. The greatest negative of temporal random effect of water vapor pressure was in January (− 0.0137), while the greatest positive effect was in June (0.0161).

The variances of the weather variables together account for 31.87% of the total variance of the hyperpriors with solar radiation being the most important (${\sigma }_{{\beta }_{3}}^{2}= 0.023)$, explaining 9.88%, while temperature is the smallest, accounting for 6.2%. The variance of the area level interaction effect (${\sigma }_{\updelta }^{2})$) accounts for the highest fraction of the total variance (43.47%), followed by the cell level interaction effect (${\sigma }_{\Phi }^{2}$), with a fraction of 24.68%. These fractions indicate that the trend of dengue relative risk for each district and subdistrict is strongly affected by neighboring districts and subdistricts, respectively. The relatively low fractions of the total variance for the other effects imply that they are less important. Specifically, the low fraction of the total variance for the average temperature indicates that only a small part of the variability of the relative risk of dengue in districts and subdistricts is explained by the average temperature.

The posterior mean of hypermeter of the Leroux CAR spatial autoregressive coefficient ($\rho )$, and the posterior means of the hyperparameters of the temporal autoregressive coefficients at area level (${\lambda }_{2}$) and cell level (${\lambda }_{1}$) are substantial (larger than 0.700), indicating strong spatial and temporal dependency. The estimated range is $r$ equals $13.597\,{\rm km}$. This implies that beyond the distance $r=13.597$ the spatial correlation among any two cells is smaller than 0.1. Hence, the observations are spatially strongly correlated. Beyond $13.597{\rm km}$ it is negligible.

Based on Table 3, we now discuss the estimated parameters for the time-varying effects of the risk factors and, next, the spatiotemporal interaction effects at the area and cell levels.

The posterior means of time-varying effects of the weather variables are presented in Fig. 6. The figure shows that the time-varying effects of precipitation, average temperature and solar radiation vary considerably over the year. The time-varying effect of water vapor pressure, in contrast, is highly constant over time.

The time-varying effect of precipitation is positive for the periods January–April, August–September and December and negative for May–July and October–November. The strongest negative effect was in June (− 0.0289), and the strongest positive effect was in March (0.0135). The negative impact for the period May–July follows after the peak of the rainy season from November–April. The negative impact for October–November is caused by the increase in precipitation after the peak of the dry season in June–July. The positive impacts for the periods January–April and August–September, and the peak in March, correspond to the relatively low rainfall one to two months before these periods.

The time-varying effect of temperature is positive for all months and ranges from 0.0289 (end of August) to 0.2106 (June–July). It increases from January–June, is at its peak in June, decreases from July until mid-September, and then starts increasing up to the global mean, where it remains for the rest of the year. Note that the monthly temperature has a delayed risk effect in that it increases from January–May, while its impact is largest in June. The delay is due to the mosquito life cycle and incubation period (Jaya and Folmer 2021a).

The time-varying coefficient of solar radiation is below zero for almost all months, except for November, and varies from − 0.0003 to 0.0002. This is due to the fact that tropical countries such as Indonesia receive a lot of solar radiation throughout the year (Handayani and Ariyanti 2012). The time-varying effect of water vapor pressure is positive all year round and hardly varies. The effect varies from 0.4896 to 0.5194. The strongest effect is in June (0.5194).

Figure 7 presents the estimated parameters of the spatiotemporal interaction effects at area and cell level which depend on their hyperparameters in Table 3.

Figure 7a presents the district level spatiotemporal interaction effects (i.e., the residual effect after accounting for the weather effects). The figure shows that the interaction effect varies across districts and time. In the northern districts, it is positive and quite high during January and February, followed by a decrease to around zero during the period March–May. From June–September it is moderately positive followed by a period of high positive interaction for the rest of the year, especially in the most north-western districts. The high interaction effect in the period September–February is related to multiple factors, in particular environmental conditions. The northern areas are ideal breeding habitats because of dense vegetation and high humidity, especially during the rainy season, with low sunshine duration and high humidity.

The central districts have high spatiotemporal interaction effects because of favorable socioeconomic conditions for the spread of the dengue virus, including high population density and high density of hotels, hostels and student apartments. For the northern central districts, there is the additional effect of spillover of mosquitoes from the northern districts. As a consequence, the interaction effect of the northern central districts follows a time pattern similar to time patterns of the northern districts, though less intense. The most central districts have high positive interaction effects all year round because they have the highest population density and density of hotels and hostels. The two most central districts have low interaction effects all year round, indicating that the main effects (risk factors) virtually fully explain the dengue risk. These districts have no special socioeconomic or environmental conditions affecting the dengue incidence rate.

In the southern districts, the spatiotemporal interaction effect is similar to that in the most central districts, although for partly different reasons. They have high population density with many residential areas that have unhygienic conditions. See Hsu et al. (2017) for details on the relationship between hygiene and dengue infection.

The situation in the eastern districts differs from that in the northern, southern and central districts. The interaction effect is negative in January and February, highly positive in March–May, slightly positive in June–August and negative for the rest of the year. The negative interaction effect in January–February is probably caused by the interaction of the weather variables and the environmental conditions. The absence of forests, heavy rainfall, and the short spells of sunshine in the period January–February keep the humidity low, which is unfavorable for the presence of dengue mosquitoes. The districts are residential areas with inadequate drainage and sanitation. The heavy rainfall until March combined with inadequate drainage and sanitation leads to large quantities of standing water, which provides favorable breeding habitats, contributing to the positive interaction effect in March.

The western districts have medium to strong negative interaction effects all year round, reducing the effects of the weather variables. The majority of the western districts have good drainage and sanitation, and the lifestyle and health behavior of the population is substantially better than in the other parts of Bandung, reducing dengue infection (Bandung Health Profile 2019). For example, the district with the highest healthy behavior index, Cicendo, is located in the western region. The negative effects also indicate that there is limited spillover of mosquitoes from the other districts.

Figure 7b presents contour maps of the cell level interaction effects. In contrast with Fig. 7a, the cell level interaction effects are almost the same across the months. Hence, after accounting for the weather variables, the cell level residual varies over space but is relatively constant over time, implying that it is related more to a topographical dimension, such as elevation, than to time. Positive cell level interaction effects are found in the northern part of Bandung, which is at 800 m above sea level and has high precipitation and dense vegetation, providing an ideal breeding ground and habitat for the *Aedes-spp* mosquito (Arboleda et al. 2009).

### Posterior mean of the relative risk

**Fig. 8:**
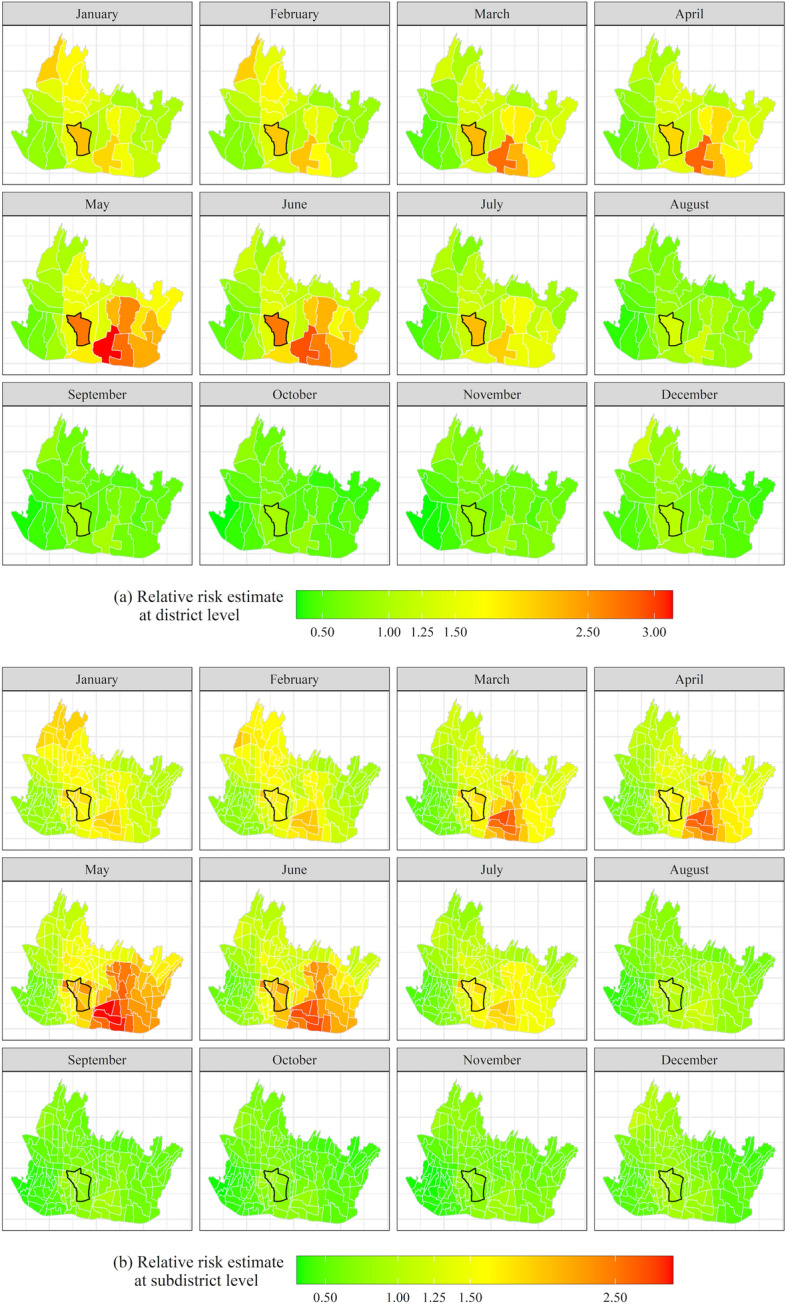
Monthly posterior means of the relative risk at **a** area (district) level and **b** subdistrict level (surrounded area: Lengkong district)

Figure 8a shows the posterior means of the relative risk estimates (based on the posterior means of the time-varying coefficients of the risk factors and the posterior means of the spatiotemporal interaction effects) at district level. The posterior means of the relative risk at subdistrict level (see Fig. 8b) are obtained as the block average of the cell values within each subdistrict boundary and the cell values that are partly outside its boundary.^22^ Accordingly, Bandung city is divided into 30 districts, 151 subdistricts and 179 cells.

**Fig. 9:**
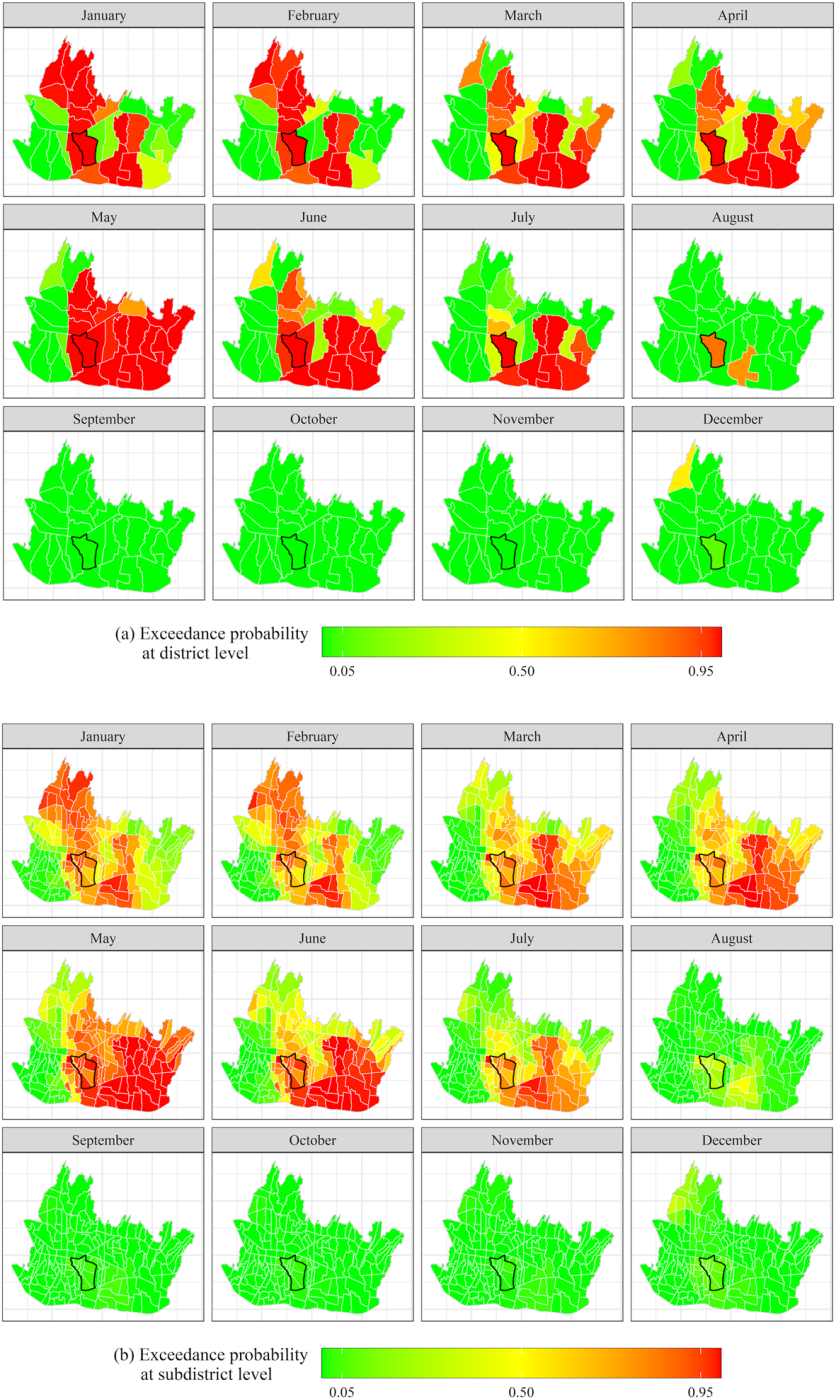
Monthly posterior exceedance probability of the relative risk at **a** district and **b** subdistrict levels (surrounded area: Lengkong district)

Comparing Figs. 8a and 8b shows similar temporal trends, in particular, an increase in January–July and a decrease in August–November. This applies especially to the spatial units with the highest risk, notably the districts in southern Bandung. For the spatial dimension, however, we notice substantial differences. For example, according to Fig. 8a, the entire central district of Lengkong (surrounded area in Fig. 8) is categorized as a high-risk area in the period January–July, whereas Fig. 8b shows that this only applies to parts of the district. The explanation is that categorization based on the model given by Eq. (3a) ignores within-district heterogeneity, while this is taken into account when categorization is based on the model given by Eq. (3b). To explore this issue further, we calculated the high-risk and low-risk districts and subdistricts based on the posterior exceedance probability for the two approaches, denoted as the top-down and the bottom-up approaches, respectively. Following Sparks (2015) and Osei and Stein (2017), we fixed the posterior exceedance probability threshold for ${\theta }_{it}\, {\rm at} 1.25$. The exceedance probability $\widehat{Pr}\left({\theta }_{it}>1.25|\mathbf{y}\right)$ over space and time is presented in Fig. 9. Table 4 presents the classification of the subdistricts into high and low risk based on the bottom-up and top-down approaches, respectively.

**Table 4:** Misclassification of the subdistricts based on the bottom-up and top-down approaches

Month	Bottom-up approach	Top-down approach		Misclassification (%)
		Low	High	
January	Low	92	53	35.1
	High	0	6	
February	Low	97	48	31.8
	High	0	6	
March	Low	105	36	24.5
	High	1	9	
April	Low	111	29	19.9
	High	1	10	
May	Low	59	54	35.8
	High	0	38	
June	Low	93	30	19.9
	High	0	28	
July	Low	119	29	19.9
	High	1	2	
August	Low	151	0	0
	High	0	0	
September	Low	151	0	0
	High	0	0	
October	Low	151	0	0
	High	0	0	
November	Low	151	0	0
	High	0	0	
December	Low	151	0	0
	High	0	0	
Average (%)	15.6			

Table 4 shows substantial misclassification for the top-down approach for the period January–July. The overall misclassification rate is 15.6% for all periods (January–December) and 26.7% for the high-risk period (January–July). The table furthermore shows that all the misclassifications occurred in the period January–July, which partly overlaps with the rainy season in November–May, whereas there is no misclassification from August–December. The explanation for the misclassification as such is that the bottom-up approach averages out the differences in risk factors, and consequently the number of incidences, over a set of relatively small number of relatively homogenous cells within a subdistrict. The top-down approach, on the other hand, averages out the differences over a substantially larger number of relatively heterogeneous cells in a district (a district contains at least four subdistricts; see Sect. 4.1).

The misclassification is obviously concentrated in the rainy period January–July with local variation in the risk factors due variation in local characteristics such as elevation or vegetation density. Although the rainy season is from November to May, there are positive rates of misclassification in June–July and unexpected zero rates in November–December. These misclassifications and unexpected rates are due to the delayed responses of mating, breeding and hunting by *Aedes-spp* mosquitos. Mating and breeding occur mainly during the rainy season. Following this, it takes approximately two weeks to one month for the eggs to develop into adult mosquitoes and for the virus to multiply and reach the salivary glands before it is transmitted to humans. If an individual is infected, the symptoms can be observed approximately four to seven days after being bitten (Ehelepola et al. 2015). Accordingly, there is a delayed infection response with respect to the weather conditions (Jaya and Folmer 2021a).

## Summary and conclusions

Effective and efficient control of a variety of spatial problems, including dengue disease abatement, requires data at a fine spatiotemporal scale. However, data availability at the same (especially fine) spatial scale is quite rare (Moraga et al. 2017; Utazi et al. 2019). A major challenge in spatial sciences, including modeling of infectious diseases such as dengue and COVID-19, is how to align data bases of different resolutions consistently. In this study, we presented the Fusion Area-Cell Spatiotemporal Generalized Geoadditive-Gaussian Markov Random Field model as a solution to this problem. This model combines observations on the dependent variable and population at risk at the area level and covariates at the cell level to generate predictions of relative risk at the subdistrict level. Special attention was paid to the model setup to generate predictions for the target cells during model-fitting, using Bayesian Integrated Nested Laplace Approximation (INLA). The methodology was applied to monthly dengue disease data for 30 districts in the city of Bandung, Indonesia, for the period January 2012 to December 2018. The risk factors consisted of the monthly averages of precipitation, temperature, solar radiation and water vapor pressure. The analysis showed that the effects of precipitation, temperature and solar radiation varied considerably across space and time, while the effect of water vapor pressure was highly constant over time. Solar radiation was found to be the most important risk factor. The spatiotemporal interaction effect, capturing the effects of omitted variables at area level, also varied across districts and time. In contrast, the cell level interaction effect was almost constant over the months but varied substantially over space, indicating a strong spatial spillover effect.

Based on the posterior means of the relative risk at cell level, we obtained the relative risk estimates at subdistrict level. We found a similar temporal pattern for district and subdistricts. Relative dengue risk was relatively high in the period January–July and relatively low during the period August–December. We further compared the risk estimates per subdistrict based on: (i) the bottom-up approach using the cell level estimates and (ii) the top-down approach assigning the district value to its subdistricts. Using the posterior exceedance probability of the relative risk, we identified high-risk and low-risk districts to find that during the high-risk period of January–July, the top-down approach misclassified 26.4% of the subdistricts as high risk, which according to the bottom-up approach was low risk. The overall misclassification rate was 15.6%.

The main conclusions of the paper are the following. First, effective and efficient policy intervention, such as the control of infectious diseases, requires data at the right level of resolution. In particular, low-resolution maps may misclassify regions. If regions are incorrectly misclassified as high-risk, unnecessary policy intervention with undue financial, social and environmental costs may result. In contrast, if regions are incorrectly misclassified as low-risk, opportunities for policy intervention may be missed which may also have costs of various kinds. Secondly, the proposed FGG-GMRF model adjusts data and maps of different resolutions consistently, and allows more data to be utilized, thus improving the statistical efficiency. Third, application of the FGG-GMRF model to the dengue disease data for Bandung from 2012 to 2018 shows that the relative infection risk is high in various cells, subdistricts and districts from January–July. The strong spatiotemporal interaction indicates that the occurrence of the dengue disease vector is highly contagious and must be detected early in order to prevent its spread. Rapid response measures such as fogging are critical in areas with high dengue incidence. Finally, based on the experiences in the present paper, it is worthwhile to investigate the suitability of the FGG-GMRF model for a variety of other spatiotemporal problems, including other infectious diseases such as COVID-19 (see Jaya and Folmer 2021b), vaccination coverage (Utazi et al. 2019), particulate matter concentration (Cameletti et al. 2013; Lee et al. 2016), and social issues such as unemployment and crime.

### Electronic supplementary material

Below is the link to the electronic supplementary material.

Supplementary file 1 (PDF 210 KB)

Supplementary file 2 (ZIP 5354 KB)

## Appendix 1: The linear stochastic partial differential equation approach

A GF with dense covariance matrix can be transformed to a GMRF with sparse covariance matrix by means of a Linear Stochastic Partial Differential Equation (LSPDE) (Lindgren et al. 2011) which reads:

34 $${\left({\kappa}^{2}-{\nabla}^{2}\right)}^{\alpha/2}{{\varvec{\gamma}}}_{t}\left({\varvec{s}}\right)={\mathcal{W}}_{t}\left({\varvec{s}}\right)\quad{\rm for}\,t=1,\ldots,T\,\,{\rm and}\,\,{\varvec{s}}={({\varvec{s}}}_{1},\ldots{{\varvec{s}}}_{g},\ldots,{{\varvec{s}}}_{{n}_{p}}){^{\prime}}\,\,{\rm with}\,\,{{\varvec{s}}}_{g}=({s}_{g,1},{s}_{g,2})$$

where ${{\varvec{\upgamma}}}_{t}\left({\varvec{s}}\right)$ is a temporally independent GF, ${\mathcal{W}}_{t}\left({\varvec{s}}\right)$ a Gaussian white noise process, $\alpha$ a positive integer related to the smoothness parameter of the Matérn covariance function in Eq. (11) by $\alpha =v+1,$ and ${\nabla }^{2}$ the Laplace operator, i.e., ${\nabla }^{2}=\left(\frac{{\partial }^{2}}{\partial {{\varvec{s}}}_{1}^{2}}+\frac{{\partial }^{2}}{\partial {{\varvec{s}}}_{2}^{2}}\right)$. Using spectral decomposition, Whittle (1954, 1963) showed that for $\alpha$ > 1, the only exact stationary solution to Eq. (34) is the isotropic Matérn field, i.e., the stationary GF ${{\varvec{\upgamma}}}_{t}\left({\varvec{s}}\right)$ with the Matérn covariance function in Eq. (11).

A closed-form solution for the LSPDE in Eq. (34) is restricted to regular lattices (Lindgren et al. 2011). For an irregular lattice, it can be approximated through a basis function representation using the Finite Element Method (FEM) defined on the domain $\mathcal{A}\in {\mathbb{R}}^{2}$. The basis function representation is defined on a mesh, that is, a collection of (i) vertices, (ii) the edges between the vertices, and (iii) the polygons described by the edges. A mesh consists of a minimum of three connected edges conforming to the shape of the domain. It subdivides a continuous geometric space into a finite set of discrete geometric or topological elements such as triangles or rectangles (for a two-dimensional geometric space) or tetrahedral or rectangular prisms (for three-dimensional spaces). It reduces the degrees of freedom from infinite to finite. Because the FEM calculations are based on a finite number of cells and the results are generalized through interpolation for the entire domain, the accuracy of the global solution is a function of the number of elements of the mesh (Bohn and Feischl 2021).

Triangulation is a common FEM meshing scheme (Sloan 1993) because of its flexibility for irregular domains (Lindquist and Gilest 1989) and accuracy (due to the minimization of the discretization error)^23^ (Ahmadian et al. 1998). Triangulation divides the domain into a set of non-intersecting triangles, where any two triangles meet in, at most, a common edge or vertex. The popular Delaunay triangulation scheme (Cheng et al. 2013) ensures that the triangulation maximizes the minimum angle of the triangles, thus avoiding sliver (i.e., long and thin) triangles and rendering the transitions between small and large triangles smooth (Lindgren et al. 2011). The restricted Bowyer–Watson algorithm, which is designed to conform to the domain’s boundary, is a popular Delaunay triangulation algorithm (Cheng et al. 2013).

A drawback of applying an algorithm with boundary conditions is that the variance near the boundary is inflated by a factor of two (Lindgren et al. 2011). To avoid the boundary effect, Lindgren and Rue (2015) proposed to extend the domain of interest by an outer area at a distance of at least $r$, corresponding to a correlation of approximately 0.1 between two points in the inner and outer areas. The edge length of the triangles of the outer area should be at least equal to the edge length of the triangles of the inner area (Blangiardo and Cameletti 2015). To find the appropriate mesh for the data at hand, meshes of different sizes are usually considered, which can be evaluated using the DIC and WAIC (Righetto et al. 2018).

Given the triangular mesh, the FEM of the solution of the LSPDE in Eq. (34) is:

35 $${{\varvec{\upgamma}}}_{t}\left({\varvec{s}}\right)\approx \sum_{l=1}^{L}{{\varvec{\psi}}}_{l}\left({\varvec{s}}\right){\stackrel{\sim }{\upgamma }}_{tl}\,\,{\rm for }\quad t=1,\ldots ,T , {\varvec{s}}={({\varvec{s}}}_{1},\ldots {{\varvec{s}}}_{g},\ldots , {{\varvec{s}}}_{{n}_{p}}){^{\prime}}\,\,{\rm and}\,\,{{\varvec{s}}}_{g}=({s}_{g,1},{s}_{g,2})$$

with ${\left\{{{\varvec{\psi}}}_{l}({\varvec{s}})\right\}}_{l=1}^{L}$ the set of piecewise linear basis functions, $L$ the number of vertices in the triangulation and *l* the $l$ th vertex. For location ${{\varvec{s}}}_{g}$, the piecewise linear basis function ${{\varvec{\psi}}}_{l}({{\varvec{s}}}_{g})$ is:

36 $${{\varvec{\psi}}}_{l}\left({{\varvec{s}}}_{g}\right)=\left\{\begin{array}{ll}1 &\quad{\rm if\, the \,vertex }\,l \,{\rm is\, at\,location }\,{{\varvec{s}}}_{g} \\ 0&\quad {\rm elsewhere}.\end{array}\right.$$

The ${\left\{{\stackrel{\sim }{\upgamma }}_{tl}\right\}}_{l=1}^{L}$ are zero mean Gaussian-distributed weights determining the value of ${{\varvec{\upgamma}}}_{t}\left({\varvec{s}}\right)$ for vertex $l$ at location ${{\varvec{s}}}_{g}$. Hence, ${{\varvec{\upgamma}}}_{t}\left({\varvec{s}}\right)$ is uniquely defined by its values at the vertices $l,\,\,l=1, 2,\ldots ,L$ of the mesh. The values in the interior of the triangles are estimated by linear interpolation. Hence, the Gaussian-distributed weights determine the values of the GF at the vertices such that the distribution of ${{\varvec{\upgamma}}}_{t}\left({\varvec{s}}\right)$ is determined by the joint distribution of the weights ${\stackrel{\sim }{{\varvec{\upgamma}}}}_{t}=\left({\stackrel{\sim }{\upgamma }}_{t1},\ldots ,{\stackrel{\sim }{\upgamma }}_{t}\right)\boldsymbol{^{\prime}}$ with sparse precision matrix ${\stackrel{\sim }{\mathbf{Q}}}_{s}$ (Lindgren et al. 2011).

To show that Eq. (34) approximates the GF ${{\varvec{\upgamma}}}_{t}\left({\varvec{s}}\right)$, we take^24^$\alpha =2$ corresponding to $v$ = 1, define the LSPDE in Eq. (34) as a variational problem, i.e., multiply it by an arbitrary test function $\boldsymbol{\varphi }\left({\varvec{s}}\right) \quad {{\rm for}}\,{\varvec{s}}={({\varvec{s}}}_{1},\ldots {{\varvec{s}}}_{g},\ldots , {{\varvec{s}}}_{{n}_{p}}){^{\prime}}$ with ${{\varvec{s}}}_{g}=({s}_{g,1},{s}_{g,2})$, and integrate it by parts over the domain $\mathcal{A}$ using Green’s first identity theorem^25^ (Langtangen and Logg 2016). Multiplying the LSPDE in Eq. (34) by a test function $\boldsymbol{\varphi }\left({\varvec{s}}\right)$ gives:

$${\int }_{\mathcal{A}}\boldsymbol{\varphi }({\varvec{s}})\left({\kappa }^{2}-{\nabla }^{2}\right){{\varvec{\upgamma}}}_{t}({\varvec{s}}){\rm d}{\varvec{s}}={\int }_{\mathcal{A}}\boldsymbol{\varphi }({\varvec{s}}){\mathcal{W}}_{t}({\varvec{s}}){\rm d}{\varvec{s}}$$

37 $${\int }_{\mathcal{A}}{\kappa }^{2}\boldsymbol{\varphi }({\varvec{s}}){{\varvec{\upgamma}}}_{t}({\varvec{s}}){\rm d}{\varvec{s}}-{\int }_{\mathcal{A}}\boldsymbol{\varphi }({\varvec{s}}){\nabla }^{2}{{\varvec{\upgamma}}}_{t}({\varvec{s}}){\rm d}{\varvec{s}}={\int }_{\mathcal{A}}\boldsymbol{\varphi }({\varvec{s}}){\mathcal{W}}_{t}({\varvec{s}}){\rm d}{\varvec{s}}$$

where $\mathcal{A}$ is the entire spatial domain over which Eq. (37) is to be solved and $d{\varvec{s}}$ is shorthand for $d{{\varvec{s}}}_{1}d{{\varvec{s}}}_{2}$ of the two-dimensional integral. Partial integration of $-{\int }_{\mathcal{A}}\boldsymbol{\varphi }({\varvec{s}}){\nabla }^{2}{{\varvec{\upgamma}}}_{t}({\varvec{s}}){\rm d}{\varvec{s}}$ gives:

38 $$-{\int }_{\mathcal{A}}\boldsymbol{\varphi }\left({\varvec{s}}\right){\nabla }^{2}{{\varvec{\upgamma}}}_{{\varvec{t}}}({\varvec{s}}){\rm d}{\varvec{s}}={\int }_{\mathcal{A}}\nabla \boldsymbol{\varphi }\left({\varvec{s}}\right)\nabla {{\varvec{\upgamma}}}_{{\varvec{t}}}({\varvec{s}}){\rm d}{\varvec{s}}-{\int }_{\partial \mathcal{A}}\frac{\partial {{\varvec{\upgamma}}}_{{\varvec{t}}}({\varvec{s}})}{\partial \mathbf{n}}\boldsymbol{\varphi }\left({\varvec{s}}\right){\rm d}\stackrel{\sim }{{\varvec{s}}}$$

where $\frac{\partial {{\varvec{\upgamma}}}_{{\varvec{t}}}({\varvec{s}})}{\partial \mathbf{n}}=\mathbf{n}\bullet \nabla {{\varvec{\upgamma}}}_{t}\left({\varvec{s}}\right)$ is the directional derivative of ${{\varvec{\upgamma}}}_{t}({\varvec{s}})$ in outward normal direction $\mathbf{n}$ on the boundary $\partial \mathcal{A}$ (i.e., the subset of points which can be approached both from $\mathcal{A}$ and from the outside of $\mathcal{A}$) (Langtangen and Logg 2016) and $d\stackrel{\sim }{{\varvec{s}}}$ is shorthand for $d{\stackrel{\sim }{{\varvec{s}}}}_{1}d{\stackrel{\sim }{{\varvec{s}}}}_{2}$ of the two-dimensional integral along the boundary $\partial \mathcal{A}$. Substituting Eq. (38) in Eq. (37), we have:

39 $${\int }_{\mathcal{A}}{\kappa }^{2}\boldsymbol{\varphi }({\varvec{s}}){{\varvec{\upgamma}}}_{t}({\varvec{s}}){\rm d}{\varvec{s}}+{\int }_{\mathcal{A}}\nabla \boldsymbol{\varphi }\left({\varvec{s}}\right)\nabla {{\varvec{\upgamma}}}_{t}({\varvec{s}}){\rm d}{\varvec{s}}-{\int }_{\partial \mathcal{A}}\frac{\partial {{\varvec{\upgamma}}}_{t}({\varvec{s}})}{\partial \mathbf{n}}\boldsymbol{\varphi }\left({\varvec{s}}\right){\rm d}{\varvec{s}}={\int }_{\mathcal{A}}\boldsymbol{\varphi }({\varvec{s}}){\mathcal{W}}_{t}({\varvec{s}}){\rm d}{\varvec{s}}$$

with the Neumann boundary condition:

$${\left.\frac{\partial {{\varvec{\upgamma}}}_{t}({\varvec{s}})}{\partial \mathbf{n}}\right|}_{\partial \mathcal{A}}=0,$$

where | denotes the boundary (Bakka 2019). Hence, Eq. (39) can be written as:

40 $${\kappa }^{2}{\int }_{\mathcal{A}}\boldsymbol{\varphi }({\varvec{s}}){{\varvec{\upgamma}}}_{t}({\varvec{s}}){\rm d}{\varvec{s}}+{\int }_{\mathcal{A}}\nabla \boldsymbol{\varphi }\left({\varvec{s}}\right)\nabla {{\varvec{\upgamma}}}_{t}({\varvec{s}}){\rm d}{\varvec{s}}={\int }_{\mathcal{A}}\boldsymbol{\varphi }\left({\varvec{s}}\right){\mathcal{W}}_{t}\left({\varvec{s}}\right){\rm d}{\varvec{s}}.$$

Because the set of the test functions is infinite **(**Langtangen and Logg 2016), it is not possible to test Eq. (40) for every test function $\boldsymbol{\varphi }({\varvec{s}})$. As a solution, the FEM can be applied to construct a finite set of test functions ${\left\{{\boldsymbol{\varphi }}_{h}\left({\varvec{s}}\right)\right\}}_{h=1}^{L} \quad {{\rm for}}\,h=1,\ldots ,L$ and tested against Eq. (40). Using Eq. (35) and substituting ${\left\{{{\varvec{\psi}}}_{l}({\varvec{s}})\right\}}_{l=1}^{L}{\rm for }l=1,\ldots ,L$ in Eq. (40), we obtain the system of linear equations:

$${\int }_{\mathcal{A}}{\kappa }^{2}{\boldsymbol{\varphi }}_{h}\left({\varvec{s}}\right)\sum_{l=1}^{L}{{\varvec{\psi}}}_{l}\left({\varvec{s}}\right){\stackrel{\sim }{\upgamma }}_{tl}{\rm d}{\varvec{s}}+{\int }_{\mathcal{A}}\nabla {\boldsymbol{\varphi }}_{h}\left({\varvec{s}}\right)\nabla \sum_{l=1}^{L}{{\varvec{\psi}}}_{l}\left({\varvec{s}}\right){\stackrel{\sim }{\upgamma }}_{tl}{\rm d}{\varvec{s}}={\int }_{\mathcal{A}}{\boldsymbol{\varphi }}_{h}\left({\varvec{s}}\right){\mathcal{W}}_{t}({\varvec{s}}){\rm d}{\varvec{s}},$$

41 $$\sum_{l=1}^{L}\left({\kappa }^{2}{\int }_{\mathcal{A}}{\boldsymbol{\varphi }}_{h}\left({\varvec{s}}\right){{\varvec{\psi}}}_{l}\left({\varvec{s}}\right){\rm d}{\varvec{s}}+{\int }_{\mathcal{A}}\nabla {\boldsymbol{\varphi }}_{h}\left({\varvec{s}}\right)\nabla {{\varvec{\psi}}}_{l}({\varvec{s}}){\rm d}{\varvec{s}}\right){\stackrel{\sim }{\upgamma }}_{tl}={\int }_{\mathcal{A}}{\boldsymbol{\varphi }}_{h}\left({\varvec{s}}\right){\mathcal{W}}_{t}({\varvec{s}}){\rm d}{\varvec{s}}.$$

The test functions are commonly taken to be equal to the basis functions, i.e., ${\boldsymbol{\varphi }}_{h}({\varvec{s}})={{\varvec{\psi}}}_{l}({\varvec{s}})$^26^ for $h=1,\ldots ,L$. Hence:

42 $$\sum_{l=1}^{L}\left({\kappa }^{2}{\int }_{\mathcal{A}}{{\varvec{\psi}}}_{l}\left({\varvec{s}}\right){{\varvec{\psi}}}_{h}\left({\varvec{s}}\right){\rm d}{\varvec{s}}+{\int }_{\mathcal{A}}\nabla {{\varvec{\psi}}}_{h}\left({\varvec{s}}\right)\nabla {{\varvec{\psi}}}_{l}({\varvec{s}}){\rm d}{\varvec{s}}\right){\stackrel{\sim }{\upgamma }}_{tl}={\int }_{\mathcal{A}}{{\varvec{\psi}}}_{l}({\varvec{s}}){\mathcal{W}}_{t}({\varvec{s}}){\rm d}{\varvec{s}}$$

The integral on the right-hand side of Eq. (42) is the Gaussian white noise distribution $\mathcal{N}\left(0,{\int }_{\mathcal{A}}{{\varvec{\psi}}}_{{\varvec{l}}}^{2}({\varvec{s}}){\rm d}{\varvec{s}}\right)$, with mean zero and covariance matrix^27^:

43 $$Cov\left({\int }_{\mathcal{A}}{{\varvec{\psi}}}_{l}\left({\varvec{s}}\right){\mathcal{W}}_{t}({\varvec{s}}){\rm d}{\varvec{s}},{\int }_{\mathcal{A}}{{\varvec{\psi}}}_{h}\left({\varvec{s}}\right){\mathcal{W}}_{t}({\varvec{s}}){\rm d}{\varvec{s}}\right)={\int }_{\mathcal{A}}{{\varvec{\psi}}}_{l}\left({\varvec{s}}\right){{\varvec{\psi}}}_{h}\left({\varvec{s}}\right){\rm d}{\varvec{s}}.$$

We can write Eq. (42) in matrix form as (Simpson et al. 2012):

44 $${{\varvec{K}}}_{{\varvec{s}}}{\stackrel{\sim }{{\varvec{\upgamma}}}}_{t}\sim \mathcal{N}\left(0,{\mathbf{C}}_{{\varvec{s}}}\right)$$

where $${{\varvec{K}}}_{{\varvec{s}}}={\upkappa }^{2}{\mathbf{C}}_{{\varvec{s}}}+{{\varvec{M}}}_{{\varvec{s}}}$$, $${\stackrel{\sim }{{\varvec{\upgamma}}}}_{t}={\left({\stackrel{\sim }{\upgamma }}_{t1},\ldots ,{\stackrel{\sim }{\upgamma }}_{tL}\right)}^{{\prime}}$$, $${{\rm C}}_{s,lh}={\int }_{\mathcal{A}}{{\varvec{\psi}}}_{l}\left({\varvec{s}}\right){{\varvec{\psi}}}_{h}\left({\varvec{s}}\right){\rm d}{\varvec{s}},$$ and $${M}_{s,lh}={\int }_{\mathcal{A}}\nabla {{\varvec{\psi}}}_{l}\left({\varvec{s}}\right).\nabla {{\varvec{\psi}}}_{h}({\varvec{s}}){\rm d}{\varvec{s}}$$.

Because of the highly local nature of the piecewise linear basis functions, ${\mathbf{C}}_{{\varvec{s}}}$ and ${\mathbf{M}}_{{\varvec{s}}}$ are sparse matrices. However, ${\mathbf{C}}_{{\varvec{s}}}^{-1}$ is a dense matrix and will generally not be a GMRF. Lindgren et al. (2011) proposed to solve this issue by reducing the integration order of the inner product of the piecewise linear basis functions ${{\varvec{\psi}}}_{l}\left({\varvec{s}}\right)$ and ${{\varvec{\psi}}}_{h}\left({\varvec{s}}\right)$ for vertices $l=1,\ldots ,L$ and $h=1,\ldots .L$ on the interval $\mathcal{A}$ (i.e., ${\langle {{\varvec{\psi}}}_{l}\left({\varvec{s}}\right),{{\varvec{\psi}}}_{h}\left({\varvec{s}}\right)\rangle }_{\mathcal{A}}={\int }_{\mathcal{A}}{{\varvec{\psi}}}_{l}\left({\varvec{s}}\right){{\varvec{\psi}}}_{h}\left({\varvec{s}}\right){\rm d}{\varvec{s}}$)^28^ by taking ${{\varvec{\psi}}}_{h}\left({\varvec{s}}\right)$ as the constant function 1 yielding ${\langle {{\varvec{\psi}}}_{l}\left({\varvec{s}}\right),1\rangle }_{\mathcal{A}}={\int }_{\mathcal{A}}{{\varvec{\psi}}}_{l}\left({\varvec{s}}\right){\rm d}{\varvec{s}}= \sum_{h=1}^{L}{{\rm C}}_{lh}$. The result is an approximate diagonal ${\mathbf{C}}_{{\varvec{s}}}$ matrix, ${\stackrel{\sim }{\mathbf{C}}}_{{\varvec{s}}},$ with diagonal elements ${\stackrel{\sim }{{\rm C}}}_{{\varvec{s}},ll}=\sum_{h=1}^{L}{{\rm C}}_{lh}$. Replacing ${\mathbf{C}}_{{\varvec{s}}}$ with ${\stackrel{\sim }{\mathbf{C}}}_{{\varvec{s}}}$ yields

45 $${\mathbf{K}}_{{\varvec{s}}}{\stackrel{\sim }{{\varvec{\upgamma}}}}_{t}\sim \mathcal{N}\left(0,{\stackrel{\sim }{\mathbf{C}}}_{{\varvec{s}}}\right)$$

and

46 $${\stackrel{\sim }{{\varvec{\upgamma}}}}_{t}\sim \mathcal{N}\left(0,{\stackrel{\sim }{\mathbf{Q}}}_{\mathbf{s}}^{-1}\right)$$

with ${\stackrel{\sim }{\mathbf{Q}}}_{\mathbf{s}}$ the precision matrix

$${\stackrel{\sim }{\mathbf{Q}}}_{\mathbf{s}}={{\mathbf{K}}_{\mathbf{s}}}^{\mathbf{^{\prime}}}{\stackrel{\sim }{\mathbf{C}}}_{\mathbf{s}}^{-1}{\mathbf{K}}_{\mathbf{s}}={\left({\upkappa }^{2}{\stackrel{\sim }{\mathbf{C}}}_{\mathbf{s}}+{\mathbf{M}}_{\mathbf{s}}\right)}^{\mathbf{^{\prime}}}{\stackrel{\sim }{\mathbf{C}}}_{\mathbf{s}}^{-1}\left({\upkappa }^{2}{\stackrel{\sim }{\mathbf{C}}}_{\mathbf{s}}+{\mathbf{M}}_{\mathbf{s}}\right)$$

47 $${\stackrel{\sim }{\mathbf{Q}}}_{\mathbf{s}}=\left({\upkappa }^{4}{\stackrel{\sim }{\mathbf{C}}}_{\mathbf{s}}+2{\upkappa }^{2}{\mathbf{M}}_{\mathbf{s}}+{\mathbf{M}}_{\mathbf{s}}{\stackrel{\sim }{\mathbf{C}}}_{\mathbf{s}}^{-1}{\mathbf{M}}_{\mathbf{s}}\right)\quad {\rm for \,every }\,t,$$

where $\upkappa =\sqrt{8v}/r.$ Because of the serial independence assumption in Eq. (9), ${\stackrel{\sim }{\mathbf{Q}}}_{\mathbf{s}}$ is constant over time.

Bolin and Lindgren (2009) compared the exact FEM approach (using ${\mathbf{C}}_{{\varvec{s}}}$ with all the elements of ${\mathbf{C}}_{{\varvec{s}}}$ calculated as a Hilbert space wavalet model such as a B-spline or a Daubechies wavalet model), and the Markov approximation (replacing ${\mathbf{C}}_{{\varvec{s}}}$ with ${\stackrel{\sim }{\mathbf{C}}}_{\mathbf{s}}$) for spatial prediction and found that the differences between both approaches in terms prediction errors are negligible.

## Appendix 2

This appendix consists of three parts. The first part is Table 5. which presents the priors and hyperpriors for the FGG-GMRF model given by Eq. (33). The second part consists of comments on the priors and hyperpriors and the final part deals with identification.

## Priors, joint priors and hyperpriors for the FGG-GMRF model

See Table 5.

**Table 5:** Priors, joint priors and hyperpriors^a^

Component		Prior	Joint prior	Hyperprior
Intercept	Exchangeable $(iid)$ Gaussian process^b^	${\beta }_{0}|{\sigma }_{{\beta }_{0}}^{2}\sim \mathcal{N}\left(0,{\sigma }_{{\beta }_{0}}^{2}\right)$ for every $i,g$ and $t$. A vague Gaussian prior with zero mean and a large variance ${\sigma }_{{\beta }_{0}}^{2}={10}^{6}$	$p\left( {\beta_{0} |\sigma_{{\beta_{0} }}^{2} } \right) \propto \exp \left( { - \frac{1}{{2\sigma_{{\beta_{0} }}^{2} }}\beta_{0}^{2} } \right)$ $\quad \quad \,\,\quad \,\quad \propto {\mathcal{N}}\left( {0,\sigma_{{\beta_{0} }}^{2} } \right)$ for every $i,g$ and $t$ with ${\sigma }_{{\beta }_{0}}^{2}={10}^{6}$	–
Global effects of the risk factors	Exchangeable $(iid)$ Gaussian process	${\beta }_{k}|{\sigma }_{{\beta }_{k}}^{2}\sim \mathcal{N}\left(0,{\sigma }_{{\beta }_{k}}^{2}\right)$ for every $i,g$ and $t$ and $k=1,\ldots ,K.$ A vague Gaussian prior with zero mean and a large variance ${\sigma }_{{\beta }_{k}}^{2}={10}^{6}$	$p\left({\varvec{\beta}}|{\mathbf{Q}}_{{\varvec{\beta}}}^{-1}\right)\propto {\rm exp}\left(-\frac{1}{2}{{\varvec{\beta}}}^{^{\prime}}{\mathbf{Q}}_{{\varvec{\beta}}}{\varvec{\beta}}\right)$ for every $i,g$ and $t$ where ${\varvec{\beta}}=\left({\beta }_{1},\ldots ,{\beta }_{K}\right)$ and ${\mathbf{Q}}_{{\varvec{\beta}}}=diag\left(\frac{1}{{\sigma }_{{\beta }_{1}}^{2}},\ldots ,\frac{1}{{\sigma }_{{\beta }_{K}}^{2}}\right)$ with ${\sigma }_{{\beta }_{k}}^{2}={10}^{6}$ for $k=1,\ldots,K$	–
Temporal random effects of the risk factors^c^	Random walks of order one (RW1) or order two (RW2)	RW1: ${\zeta }_{k,t}={\zeta }_{k,t-1}+{u}_{k,t}$ for $t=2,\ldots ,T$ with ${u}_{k,t}\sim \mathcal{N}\left(0,{\sigma }_{{\zeta }_{k}}^{2}\right)$ for every $i$ and $g$	$p\left( {{\varvec{\zeta}}_{k} {|}{\mathbf{Q}}_{{\zeta_{k} }}^{ - 1} } \right) \propto \exp \left( { - \frac{1}{2}{\varvec{\zeta}}_{k}^{{^{\prime}}} {\mathbf{Q}}_{{\zeta_{k} }} {\varvec{\zeta}}_{k} } \right)$ $\quad \quad \,\quad \,\quad \propto {\mathcal{N}}\left( {0,{\mathbf{Q}}_{{\zeta_{k} }}^{ - 1} } \right)$ for every $i$ and$g$, for $k=1,\ldots ,K$ with ${{\varvec{\zeta}}}_{k}=\left({\zeta }_{k,1},\ldots ,{\zeta }_{k,T}\right)\boldsymbol{^{\prime}}$ and ${\mathbf{Q}}_{{\zeta }_{k}}$ the **(**$T\times T)$ precision matrix of the parameters ${\zeta }_{k}$ given by ${\mathbf{Q}}_{{\zeta }_{k}}=\frac{1}{{\sigma }_{{\zeta }_{k}}^{2}}{\mathbf{R}}_{{\zeta }_{k}}$ with ${\mathbf{R}}_{{\zeta }_{k}}$ the ($T\times T)$ temporal structure matrix. For the RW1 model, ${\mathbf{R}}_{{\zeta }_{k}(T\times {\varvec{T}})}^{(RW1)}$ is: $${\mathbf{R}}_{{\zeta_{k} \left( {T \times {\varvec{T}}} \right)}}^{{\left( {RW1} \right)}} = \left[ {\begin{array}{*{20}l} 1 \hfill & { - 1} \hfill & {} \hfill & {} \hfill & {} \hfill & {} \hfill & {} \hfill \\ { - 1} \hfill & 2 \hfill & { - 1} \hfill & {} \hfill & {} \hfill & {} \hfill & {} \hfill \\ {} \hfill & { - 1} \hfill & 2 \hfill & 1 \hfill & {} \hfill & {} \hfill & {} \hfill \\ {} \hfill & {} \hfill & \ddots \hfill & \ddots \hfill & \ddots \hfill & {} \hfill & {} \hfill \\ {} \hfill & {} \hfill & {} \hfill & { - 1} \hfill & 2 \hfill & { - 1} \hfill & {} \hfill \\ {} \hfill & {} \hfill & {} \hfill & {} \hfill & { - 1} \hfill & 2 \hfill & { - 1} \hfill \\ {} \hfill & {} \hfill & {} \hfill & {} \hfill & {} \hfill & { - 1} \hfill & 1 \hfill \\ \end{array} } \right]$$	Inverse Gamma (IG) with shape and scale parameters 1 and 0.01, respectively, i.e.,${\sigma }_{{\zeta }_{k}}^{2}\sim {\rm IG}\left(1, 0.01\right)$
		RW2: ${\zeta }_{k,t}=2{\zeta }_{k,t-1}-{\zeta }_{k,t-2}+{u}_{k,t}$ for $t=3,\ldots ,T$ with ${u}_{k,t}\sim \mathcal{N}\left(0,{\sigma }_{{\zeta }_{k}}^{2}\right)$ for every $i$ and $g$	For the RW2 model, ${\mathbf{R}}_{{\zeta }_{k}(T\times {\varvec{T}})}^{(RW2)}$ is: $${\mathbf{R}}_{{\zeta_{k} \left( {T \times {\varvec{T}}} \right)}}^{{\left( {RW1} \right)}} = \left[ {\begin{array}{*{20}l} 1 \hfill & { - 2} \hfill & 1 \hfill & {} \hfill & {} \hfill & {} \hfill & {} \hfill & {} \hfill \\ { - 2} \hfill & 5 \hfill & { - 4} \hfill & 1 \hfill & {} \hfill & {} \hfill & {} \hfill & {} \hfill \\ 1 \hfill & { - 4} \hfill & 6 \hfill & { - 4} \hfill & 1 \hfill & {} \hfill & {} \hfill & {} \hfill \\ {} \hfill & \ddots \hfill & \ddots \hfill & \ddots \hfill & \ddots \hfill & \ddots \hfill & {} \hfill & {} \hfill \\ {} \hfill & {} \hfill & 1 \hfill & { - 4} \hfill & 6 \hfill & { - 4} \hfill & 1 \hfill & {} \hfill \\ {} \hfill & {} \hfill & {} \hfill & 1 \hfill & { - 4} \hfill & 6 \hfill & { - 4} \hfill & 1 \hfill \\ {} \hfill & {} \hfill & {} \hfill & {} \hfill & 1 \hfill & { - 4} \hfill & 5 \hfill & { - 2} \hfill \\ {} \hfill & {} \hfill & {} \hfill & {} \hfill & {} \hfill & 1 \hfill & { - 2} \hfill & 1 \hfill \\ \end{array} } \right]$$ The joint prior of ${\varvec{\zeta}}=\left({{\varvec{\zeta}}}_{1},\ldots ,{{\varvec{\zeta}}}_{{\varvec{K}}}\right)\boldsymbol{^{\prime}}$, $p\left({\varvec{\zeta}}|{\mathbf{Q}}_{{\zeta }_{1}}^{-1},\ldots ,{\mathbf{Q}}_{{\zeta }_{K}}^{-1}\right)$ is: $p\left({\varvec{\zeta}}|{\mathbf{Q}}_{{{\varvec{\zeta}}}_{1}}^{-1},\ldots ,{\mathbf{Q}}_{{{\varvec{\zeta}}}_{K}}^{-1}\right)=\prod_{k=1}^{K}p\left({{\varvec{\zeta}}}_{k}|{\mathbf{Q}}_{{{\varvec{\zeta}}}_{k}}^{-1}\right) \propto \prod_{k=1}^{K}\mathcal{N}\left(0,{\mathbf{Q}}_{{{\varvec{\zeta}}}_{k}}^{-1}\right)$	
Spatially structured random effect	Leroux Conditional Autoregressive (${\rm CAR})$	${\omega }_{i}|({{\varvec{\omega}}}_{-i},{\varvec{W}})\sim \mathcal{N}\left(\mu ,{\sigma }^{2}\right)$ for every $t$ and $i=1,\ldots ,{n}_{\mathcal{A}},$ with $\mu =\frac{\rho \sum_{j=1}^{n}{w}_{ij}{\omega }_{j}}{\rho \sum_{j=1}^{n}{w}_{ij}+1-\rho }$ ${\sigma }^{2}=\frac{{\sigma }_{\omega }^{2}}{\left(\rho \sum_{j=1}^{n}{w}_{ij}+1-\rho \right)}$ and ${\varvec{W}}$ the (${n}_{\mathcal{A}} \times {n}_{\mathcal{A}}$) binary spatial weights matrix characterizing the neighborhood structure of the areas, e.g., an inverse distance matrix or a contiguity matrix and $\rho$ the spatial autoregressive coefficient	$p\left({\varvec{\omega}}|{\sigma }_{\omega }^{2}\right)\propto {\rm exp}\left(-\frac{1}{2}{{\varvec{\omega}}}^{^{\prime}}{{\varvec{Q}}}_{\omega }{\varvec{\omega}}\right)$ $\propto \mathcal{N}\left(0,{\mathbf{Q}}_{\upomega }^{-1}\right)$ for every $t$, with ${\varvec{\omega}}=\left({\omega }_{1},\ldots,{\omega }_{{n}_{\mathcal{A}}}\right)\boldsymbol{^{\prime}}$ and ${\mathbf{Q}}_{\upomega }={\uptau }_{\upomega }{\mathbf{R}}_{\upomega }$ the precision matrix with ${\uptau }_{\upomega }=1/{\upsigma }_{\upomega }^{2}$, and ${\mathbf{R}}_{\upomega }$ the (${n}_{\mathcal{A}} \times {n}_{\mathcal{A}})$ spatial structure matrix, with the $(i,j)$ th element defined as: $$R_{\omega } [i,j] = \left\{ {\begin{array}{*{20}l} {\rho nA_{i} + (1 - \rho )} \hfill & {{\text{if}}\,i = j} \hfill \\ { - 1} \hfill & {{\text{if}}\,i\sim \,j} \hfill \\ 0 \hfill & {{\text{otherwise}}} \hfill \\ \end{array} } \right.$$ where ${{\rm n}}_{{A}_{i}}$ is the number of neighbors in region ${\mathcal{A}}_{i}$, and $i\sim j$ denoting that areas $i$ and $j$ are neighbors	Half Cauchy (HC) with scale parameter 25 for ${\sigma }_{\omega }$ i.e., ${\sigma }_{\omega }\sim {C}^{+}({\rm 0,25})$ and ${\rm log}\left(\frac{\rho }{1-\rho }\right)\sim \mathcal{N}({\rm 0,0.45})$
Spatially unstructured random effect	Exchangeable (iid) Gaussian process	${\upsilon }_{i}|{\sigma }_{\upsilon }^{2}\sim \mathcal{N}\left(0,{\sigma }_{\upsilon }^{2}\right)$ for every $t$ and $i=1,\ldots ,{n}_{\mathcal{A}}.$	$p\left({\varvec{\upsilon}}|{\sigma }_{\upsilon }^{2}\right)\propto {\rm exp}\left(-\frac{1}{2}{{\varvec{\upsilon}}}^{^{\prime}}{{\varvec{Q}}}_{\upsilon }{\varvec{\upsilon}}\right)$ $\propto \mathcal{N}\left(0,{\mathbf{Q}}_{\upsilon }^{-1}\right)$ for every $t$, with ${\varvec{\upsilon}}=\left({\upsilon }_{1},\ldots,{\upsilon }_{{n}_{\mathcal{A}}}\right)\boldsymbol{^{\prime}}$ and ${{\varvec{Q}}}_{\upsilon }={\tau }_{\upsilon }{{\varvec{I}}}_{{n}_{\mathcal{A}}}$ the precision matrix, ${{\varvec{I}}}_{{n}_{\mathcal{A}}}$ the $({n}_{\mathcal{A}}\times {n}_{\mathcal{A}})$ identity matrix and ${\tau }_{\upsilon }=1/{\sigma }_{\upsilon }^{2}$	${\sigma }_{\upsilon }^{2} \sim {\rm IG}\left(1, 0.01\right)$
Temporally structured random effect	Autoregressive of order one (AR1)	${\phi }_{t}={\lambda }_{2}{\phi }_{t-1}+{\epsilon }_{t}$, ${\epsilon }_{t}\sim \mathcal{N}\left(0,{\sigma }_{\phi }^{2}\right)$ for every $i$ and $g$ and $t=2,\ldots,T$, with $\left|{\lambda }_{2}\right|<1$ the autoregressive parameter and ${\phi }_{1}\sim \mathcal{N}\left(0,\frac{{\sigma }_{\phi }^{2}}{1-{\lambda }_{2}^{2}}\right)$	$p\left({\varvec{\phi}}|{\mathbf{Q}}_{\phi }^{-1}\right)\propto {\rm exp}\left(-\frac{1}{2}{{\varvec{\phi}}}^{^{\prime}}{\mathbf{Q}}_{\phi }{\varvec{\phi}}\right)$ $\propto$ $\mathcal{N}\left(0,{\mathbf{Q}}_{\phi }^{-1}\right)$ for every $i$ and $g$, with ${\varvec{\phi}}=\left({\phi }_{1},\ldots,{\phi }_{T}\right)\boldsymbol{^{\prime}}$ and ${\mathbf{Q}}_{\phi }={\tau }_{\phi }{{\varvec{R}}}_{\phi \left(T\times T\right)}$ the precision matrix with ${\tau }_{\phi }=1/{\sigma }_{\phi }^{2}$, and ${{\varvec{R}}}_{\phi \left(T\times T\right)}$ the ($T\times T)$ temporal structure matrix for the $AR1$ prior: $${\varvec{R}}_{{\phi \left( {T \times T} \right)}} = \left[ {\begin{array}{*{20}l} 1 \hfill & {} \hfill & { - \lambda_{2} } \hfill & {} \hfill & {} \hfill & {} \hfill & {} \hfill \\ { - \lambda_{2} } \hfill & {} \hfill & {(1 + \lambda_{2}^{2} )} \hfill & {} \hfill & { - \lambda_{2} } \hfill & {} \hfill & {} \hfill \\ {} \hfill & \ddots \hfill & {} \hfill & \ddots \hfill & {} \hfill & \ddots \hfill & {} \hfill \\ {} \hfill & {} \hfill & { - \lambda_{2} } \hfill & {} \hfill & {(1 + \lambda_{2}^{2} )} \hfill & {} \hfill & { - \lambda_{2} } \hfill \\ {} \hfill & {} \hfill & {} \hfill & {} \hfill & { - \lambda_{2} } \hfill & {} \hfill & 1 \hfill \\ \end{array} } \right]$$	${\sigma }_{\phi }^{2} \sim {\rm IG}\left(1, 0.01\right)$
Temporally unstructured random effect	Exchangeable (iid) Gaussian	${\varsigma }_{t}|{\sigma }_{\varsigma }^{2}\sim \mathcal{N}\left(0,{\sigma }_{\varsigma }^{2}\right)$ for every $i$ and $g$ and $t=1,\ldots ,T$	$p\left(\varvec{\varsigma }|{\mathbf{Q}}_{\boldsymbol{\varsigma }}^{-1}\right)\propto {\rm exp}\left(-\frac{1}{2}{\boldsymbol{\varsigma }}^{^{\prime}}{\mathbf{Q}}_{\varsigma }\boldsymbol{\varsigma }\right)$ $\propto$ $\mathcal{N}\left(0,{\mathbf{Q}}_{\varvec{\varsigma }}^{-1}\right)$ for every $i$ and $g$, with $\varvec{\varsigma }=\left({{\varsigma }}_{1},\ldots,{{\varsigma }}_{T}\right)^{\prime}$ and ${\mathbf{Q}}_{\varsigma }={\tau }_{\varsigma }{\mathbf{I}}_{T}$ the precision matrix with ${\tau }_{\varsigma }=1/{\sigma }_{\varsigma }^{2}$ and ${\mathbf{I}}_{T}$ the ($T\times T)$ identity matrix	${\sigma }_{\boldsymbol{\varsigma }}^{2} \sim {\rm IG}\left(1, 0.01\right)$
Area level Interaction effect	Type I: combines the spatially unstructured (${\upsilon }_{i}$) and temporally unstructured $\left({\varsigma }_{t}\right)$ random effects. Implies $\rho =0$ and ${\lambda }_{2}=0.$		$p\left({\varvec{\updelta}}|{\mathbf{Q}}_{\delta (I)}^{-1}\right)\propto {\rm exp}\left(-\frac{1}{2}{{\varvec{\updelta}}}^{\boldsymbol{^{\prime}}}{\mathbf{Q}}_{\delta \left(I\right)}{\varvec{\updelta}}\right)$ $\propto \mathcal{N}\left(0,{\mathbf{Q}}_{{\varvec{\updelta}}(\mathbf{I})}^{-1}\right)$ where ${\varvec{\updelta}}={\left({\delta }_{11},\ldots,{\delta }_{{n}_{\mathcal{A}}T}\right)}^{{^{\prime}}}$ with ${\delta }_{it}$ independent over space and time for $i=1,\ldots,{n}_{\mathcal{A}},$ and $t=1,\dots ,T$. ${\mathbf{Q}}_{\delta (I)}={\tau }_{\delta }{\mathbf{R}}_{\delta (I)}$ the precision matrix with ${\tau }_{\delta }=1/{\sigma }_{\delta }^{2}$ and structure matrix ${{\mathbf{R}}_{\delta (I)}=\mathbf{R}}_{\upsilon }\otimes {\mathbf{R}}_{\varsigma }={\mathbf{I}}_{{n}_{\mathcal{A}}}\otimes {\mathbf{I}}_{T}={\mathbf{I}}_{{n}_{\mathcal{A}}T}$ with ${\mathbf{I}}_{{n}_{\mathcal{A}}T}$ the (${n}_{\mathcal{A}}T\times {n}_{\mathcal{A}}T)$ identity matrix and $\otimes$ the Kronecker product	${\sigma }_{\upsilon }^{2}\sim {\rm IG}\left(1, 0.01\right)$ and ${\sigma }_{\varsigma }^{2}\sim {\rm IG}\left(1, 0.01\right)$
	Type II: combines the spatially unstructured $\left({\upsilon }_{i}\right)$ and the temporally structured $\left({\phi }_{t}\right)$ random effects. Implies $\rho =0$ and ${\lambda }_{2}\ne 0.$		$p\left({\varvec{\updelta}}|{\mathbf{Q}}_{\delta \left(II\right)}^{-1}\right)\propto {\rm exp}\left(-\frac{1}{2}{{\varvec{\updelta}}}^{\boldsymbol{^{\prime}}}{\mathbf{Q}}_{\delta \left(II\right)}{\varvec{\updelta}}\right)$ $\propto \mathcal{N}\left(0,{\mathbf{Q}}_{{\varvec{\updelta}}(\mathbf{I}\mathbf{I})}^{-1}\right)$ where ${\varvec{\updelta}}={\left({{\varvec{\updelta}}}_{1},\dots ,{{\varvec{\updelta}}}_{{n}_{\mathcal{A}}}\right)}^{\boldsymbol{^{\prime}}}$ with ${{\varvec{\updelta}}}_{i}={\left({\delta }_{it},\dots ,{\delta }_{iT}\right)}^{\boldsymbol{^{\prime}}}$ ${\rm for} i=1,\dots ,{n}_{\mathcal{A}}$ following an AR1, independently distributed for all areas. ${\mathbf{Q}}_{\delta \left(II\right)}={\tau }_{\delta }{\mathbf{R}}_{\delta \left(II\right)}$ the precision matrix with ${\tau }_{\delta }=\frac{1}{{\sigma }_{\delta }^{2}}$ and structure matrix ${\mathbf{R}}_{\delta \left(II\right)}= {\mathbf{R}}_{\upsilon }\otimes {\mathbf{R}}_{\phi }={\mathbf{I}}_{{n}_{\mathcal{A}}}\otimes {\mathbf{R}}_{\phi }$	${\sigma }_{\upsilon }^{2}\sim {\rm IG}\left(1, 0.01\right)$ and ${\sigma }_{\varphi }^{2}\sim {\rm IG}\left(1, 0.01\right)$
	Type III: combines the temporally unstructured $\left({\varsigma }_{t}\right)$ and the spatially structured $\left({\omega }_{i}\right)$ random effects. Implies $\rho \ne 0$ and ${\lambda }_{2}=0$		$p\left({\varvec{\updelta}}|{\mathbf{Q}}_{\delta (III)}^{-1}\right)\propto {\rm exp}\left(-\frac{1}{2}{{\varvec{\updelta}}}^{\boldsymbol{^{\prime}}}{\mathbf{Q}}_{\delta (III)}{\varvec{\updelta}}\right)$ $\propto \mathcal{N}\left(0,{\mathbf{Q}}_{{\varvec{\updelta}}(\mathbf{I}\mathbf{I}\mathbf{I})}^{-1}\right)$ where ${\varvec{\updelta}}={\left({{\varvec{\delta}}}_{1},\dots ,{{\varvec{\delta}}}_{T}\right)}^{\boldsymbol{^{\prime}}}$ with ${{\varvec{\updelta}}}_{t}={\left({\delta }_{it},\dots ,{\delta }_{{n}_{\mathcal{A}}t}\right)}^{\boldsymbol{^{\prime}}}$ for $t=1,\dots ,T$ following a Leroux CAR, independently distributed for all periods. ${\mathbf{Q}}_{\delta \left(III\right)}={\tau }_{\delta }{\mathbf{R}}_{\delta \left(III\right)}$ the precision matrix with ${\tau }_{\delta }=\frac{1}{{\sigma }_{\delta }^{2}}$ and structure matrix ${\mathbf{R}}_{\delta \left(III\right)}= {\mathbf{R}}_{\varsigma }\otimes {\mathbf{R}}_{\upomega }={\mathbf{I}}_{T}\otimes {\mathbf{R}}_{\upomega }$	HC prior with scale parameter 25 for ${\sigma }_{\omega }\sim {C}^{+}({\rm 0,25})$, ${\rm log}\left(\frac{\rho }{1-\rho }\right)\sim \mathcal{N}({\rm 0,0.45})$ and ${\sigma }_{\upsilon }^{2} \sim {\rm IG}\left(1, 0.01\right)$
	Type IV: combines the spatially (${\omega }_{i} )$ and temporally structured (${\phi }_{t}$) random effects. Implies $\rho \ne 0$ and${\lambda }_{2}\ne 0$		$p\left({\varvec{\updelta}}|{\mathbf{Q}}_{\delta (IV)}^{-1}\right)\propto {\rm exp}\left(-\frac{1}{2}{{\varvec{\updelta}}}^{\boldsymbol{^{\prime}}}{\mathbf{Q}}_{\delta (IV)}{\varvec{\updelta}}\right),$ $\propto \mathcal{N}\left(0,{\mathbf{Q}}_{{\varvec{\updelta}}(\mathbf{I}\mathbf{V})}^{-1}\right)$ where ${\varvec{\updelta}}={\left({\delta }_{11},\dots ,{\delta }_{{n}_{\mathcal{A}}T}\right)}^{\boldsymbol{^{\prime}}}$ with ${\delta }_{it}$ for $i=1,\ldots,{n}_{\mathcal{A}},$ and $t=1,\dots ,T$ dependent over space and time. ${\mathbf{Q}}_{\delta \left(IV\right)}={\tau }_{\delta }{\mathbf{R}}_{\delta \left(IV\right)}$ the precision matrix with ${\tau }_{\delta }=\frac{1}{{\sigma }_{\delta }^{2}}$ and structure matrix ${\mathbf{R}}_{\delta \left(IV\right)}= {\mathbf{R}}_{\upomega }\otimes {\mathbf{R}}_{\phi }$	HC prior with scale parameter 25 for ${\sigma }_{\omega }\sim {C}^{+}({\rm 0,25})$, ${\rm log}\left(\frac{\rho }{1-\rho }\right)\sim \mathcal{N}({\rm 0,0.45})$ and ${\sigma }_{\phi }^{2} \sim {\rm IG}\left(1, 0.01\right)$
Cell level Gaussian Field through LSPDE model	${\stackrel{\sim }{{\varvec{\Phi}}}}_{t}={\lambda }_{1}{\stackrel{\sim }{{\varvec{\Phi}}}}_{t-1}+{\stackrel{\sim }{{\varvec{\upgamma}}}}_{t}\boldsymbol{ }{\rm with}\boldsymbol{ }{\stackrel{\sim }{{\varvec{\upgamma}}}}_{t}\left({\varvec{s}}\right)\sim \mathcal{N}\left(0,{\stackrel{\sim }{\mathbf{Q}}}_{s}^{-1}\right)$ for $t=1,\dots ,T$. I Initial value ${\stackrel{\sim }{{\varvec{\Phi}}}}_{1}\sim \mathcal{N}\left(0,{\stackrel{\sim }{\mathbf{Q}}}_{s}^{-1}/\left(1-{\lambda }_{1}^{2}\right)\right)$		$p\left(\stackrel{\sim }{{\varvec{\Phi}}}|{\mathbf{Q}}_{\stackrel{\sim }{{\varvec{\Phi}}}}^{-1}\right)\propto {\rm exp}\left(-\frac{1}{2}{\stackrel{\sim }{{\varvec{\Phi}}}}^{\boldsymbol{^{\prime}}}{\mathbf{Q}}_{\stackrel{\sim }{{\varvec{\Phi}}}}\stackrel{\sim }{{\varvec{\Phi}}}\right)$ $\propto \mathcal{N}\left(0,{\mathbf{Q}}_{\stackrel{\sim }{{\varvec{\Phi}}}}^{-1}\right)$ with $\stackrel{\sim }{{\varvec{\Phi}}}=({\stackrel{\sim }{{\varvec{\Phi}}}}_{1},\dots ,{\stackrel{\sim }{{\varvec{\Phi}}}}_{T})\mathbf{^{\prime}}$ and ${\mathbf{Q}}_{\stackrel{\sim }{{\varvec{\Phi}}}}={\mathbf{Q}}_{\mathbf{T}}\otimes {\stackrel{\sim }{\mathbf{Q}}}_{s}$ the $(TL\times TL)$ precision matrix with $${\mathbf{Q}}_{{{\mathbf{T}}(T \times T)}} = \left[ {\begin{array}{*{20}c} {1{/}\sigma_{\gamma }^{2} } & { - \lambda_{1} {/}\sigma_{\gamma }^{2} } & {} & {} \\ { - \lambda_{1} {/}\sigma_{\gamma }^{2} } & {(1 + \lambda_{1}^{2} {)/}\sigma_{\gamma }^{2} } & { - \lambda_{1} {/}\sigma_{\gamma }^{2} } & {} \\ \ddots & \ddots & \ddots & {} \\ {} & { - \lambda_{1} {/}\sigma_{\gamma }^{2} } & {(1 + \lambda_{1}^{2} {)/}\sigma_{\gamma }^{2} } & { - \lambda_{1} {/}\sigma_{\gamma }^{2} } \\ {} & {} & { - \lambda_{1} {/}\sigma_{\gamma }^{2} } & {1{/}\sigma_{\gamma }^{2} } \\ \end{array} } \right]$$ the $T$-dimensional precision matrix of the (AR1) process and ${\stackrel{\sim }{\mathbf{Q}}}_{\mathbf{s}}=\left({\upkappa }^{4}{\stackrel{\sim }{\mathbf{C}}}_{\mathbf{s}}+2{\upkappa }^{2}{\mathbf{M}}_{\mathbf{s}}+{\mathbf{M}}_{\mathbf{s}}{\stackrel{\sim }{\mathbf{C}}}_{\mathbf{s}}^{-1}{\mathbf{M}}_{\mathbf{s}}\right)$ the ($L\times L$) spatial precision matrix of the GMRF	Penalized complexity (PC) distribution for ${\sigma }_{\gamma }$ and $r,$ i.e., ${\rm Pr}\left({\sigma }_{\Phi }>1\right)=0.01$ and ${\rm Pr}\left(r<5\right)=0.5$, respectively

^a^Table 5 is partly based on Jaya and Folmer (2021a)

^b^An exchangeable (Gaussian) process is a sequence of random variables that are independent and identically (normal) distributed $\left({\rm iid}\right).$

^c^Scaling is applied to the temporal structure matrices of RW1 and RW2 to make their marginal variances comparable. Scaling is implemented in R-INLA by using the option scale.model = TRUE

## Comments on the priors and hyperpriors

The following observations apply for the parameters and hyperparameters of the FGG-GMRF. Due to the lack of strong prior knowledge, we used a vague Gaussian prior distribution with a zero mean and a very large variance for the parameters ${\beta }_{0}: {\beta }_{0}\sim \mathcal{N}\left(0,{10}^{6}\right)$ and ${\beta }_{k}: {\beta }_{k}\sim \mathcal{N}\left(0,{10}^{6}\right)$ for $k=1,\ldots ,K$(Blangiardo and Cameletti 2015; Martinez-Beneito and Botella-Rocamora 2019). A weakly informative prior^29^ was assigned to the log-odds of the spatial autoregressive parameter $\rho :{\rm log}\left(\rho /(1-\rho )\right)\sim \mathcal{N}\left(0, 0.45\right)$ (Utazi et al. 2019). Note that the transformation is used to ensure that $\rho$ takes values between 0 and 1 (Bivand et al. 2015; Martinez-Beneito and Botella-Rocamora 2019).

For the scale hyperparameters (variance and standard deviation) of the temporally varying coefficients, spatially and temporally structured and unstructured random effects, spatiotemporal interaction of the random effects, and the Gaussian field (GF), the Inverse Gamma (IG), half-Cauchy (HC), Uniform and Penalized Complexity (PC) are common hyperpriors. The IG distribution is a well-known, easy to use, conjugate hyperprior for the variance (Coly et al. 2021; Hamura et al. 2021).^30^ The HC is a Cauchy distribution truncated at zero. Gelman (2006) recommended the HC with scale parameter 25 as an alternative to the IG when the variance hyperparameter is close to zero. The HC is also a frequently used hyperprior for the standard deviation (Gómez-Rubio 2020). When the limit of the scale parameter goes to infinity, the HC converges to the Uniform hyperprior (Gelman 2006; Gómez-Rubio 2020). However, for the Uniform hyperprior, it is difficult to set ranges of values for the standard deviation using R-INLA. The reason is that R-INLA assumes that the standard deviation is unbounded (above) (Gelman et al. 2017; Gómez-Rubio 2020), whereas a fixed upper bound on the standard deviation is frequently needed to avoid overfitting^31^ (Simpson et al. 2017). As an alternative to the HC and Uniform hyperpriors for the standard deviation, Simpson et al. (2017) proposed the PC hyperprior. The PC allows using probability statements on values for the parameters such as the standard deviation, autocorrelation and range parameters (Franco-Villoria et al. 2019). The parameters can be restricted using either an upper bound or a lower bound but not both. The PC is frequently used for autoregressive processes (Sørbye and Rue 2017), varying coefficients models and high-resolution prediction models (Franco-Villoria et al. 2019).

In the application, we assigned the IG (1, 0.01) hyperprior to the variance of the autoregressive AR1 model of the temporally structured effects (${\sigma }_{\phi }^{2}$) and to the variances of the exchangeable (iid) priors of the spatially (${\sigma }_{\upsilon }^{2})$ and temporally (${\sigma }_{\varsigma }^{2})$ unstructured random effects, respectively. Following Lawson et al. (2010), we assigned the HC hyperprior with scale parameter to the standard deviation of the Leroux prior﻿ (Leroux et al. 2000) of the spatially structured random effect $\left({\sigma }_{\omega }^{2}\right)$.

Regarding the hyperpriors to the variances of the RW1 and RW2 priors the following applies. The marginal variances of the RW1 and RW2 priors reflect the smoothness of the $k$th vector of the temporal random effect ${{\varvec{\zeta}}}_{k}={\left({\zeta }_{k,1},\ldots ,{\zeta }_{k,T}\right)}^{{{\prime}}} {\rm for }k=1,\ldots ,K$.^32^ However, before assigning hyperpriors we need to render the marginal variances as smoothness indicators comparable as they are affected by their temporal structures and the scale of the risk factors which render them inadequate as smoothness indicators (Sørbye and Rue 2014; Blangiardo and Cameletti 2015; Gómez-Rubio 2020; Kang et al. 2015). The temporal structure matrices carry over to the hyperpriors. To overcome the incomparability problem, Sørbye and Rue (2014) proposed to scale the temporal structure matrices by the geometric means of the diagonal elements of their inverses. However, the random walk models of order one (RW1) and two (RW2) are intrinsic Gaussian Markov random fields (IGMRFs).^33^ They have zero mean and generalized covariance matrices ${{\varvec{\Sigma}}}_{{\zeta }_{k}}^{RW1}={\sigma }_{{\zeta }_{k}}^{2}{\mathbf{R}}_{{\zeta }_{k}}^{RW1-}$ and ${{\varvec{\Sigma}}}_{{\zeta }_{k}}^{RW2}={\sigma }_{{\zeta }_{k}}^{2}{\mathbf{R}}_{{\zeta }_{k}}^{RW2-}$, respectively with ${\mathbf{R}}_{{\zeta }_{k}}^{RW1-}$ and ${\mathbf{R}}_{{\zeta }_{k}}^{RW2-}$ the generalized inverses of the temporal structure matrices varying along with RW1 and RW2, respectively.

Sørbye and Rue (2014) proposed to scale the temporal structure matrices of the RW1 and RW2 priors by the geometric mean of the corresponding diagonal elements of their generalized inverses ${\mathbf{R}}_{{\zeta }_{k}}^{-}$:

$${\rm exp}\left(\frac{1}{T}\sum_{t=1}^{T}{\rm log}\left({\rm diag}\left({\mathbf{R}}_{{\zeta }_{k}}^{-}\right)\right)\right)={\sigma }_{GV}^{2}$$

with ${\sigma }_{GV}^{2}$ called the generalized variance of the diagonal elements of ${\mathbf{R}}_{{\zeta }_{k}}^{-}$ (see Sørbye and Rue (2014) for details). Scaling of ${{\varvec{R}}}_{{\zeta }_{k}}$ gives the scaled temporal structure matrix ${\stackrel{\sim }{{\varvec{R}}}}_{{\zeta }_{k}}={{\varvec{R}}}_{{\zeta }_{k}}{\sigma }_{GV}^{2}$ and scaled generalized covariance matrix as ${\stackrel{\sim }{{\varvec{\Sigma}}}}_{{\zeta }_{k}}={\sigma }_{{\zeta }_{k}}^{2}{\stackrel{\sim }{{\varvec{R}}}}_{{\zeta }_{k}}^{-}$ with scaled generalized variance ${\stackrel{\sim }{\sigma }}_{GV}^{2}=1$.

Scaling the RW1 and RW2 priors can be straightforwardly done in R-INLA by using the option scale.model = TRUE (Blangiardo and Cameletti 2015; Sørbye 2013). After scaling, the same hyperprior IG (1, 0.01) can be assigned to the variance ${\sigma }_{{\zeta }_{k}}^{2}$ of the IGMRF priors RW1 and RW2.^34^ We followed this procedure in the application.

To avoid overfitting, we followed Fuglstad et al. (2020) and applied a weakly informative PC hyperprior to the range $\left(r\right)$ and the standard deviation (${\sigma }_{\Phi }$) of the LSPDE model. Particularly, we applied ${\rm Pr}\left({\sigma }_{\Phi }>{\sigma }_{\Phi 0}\right)={\alpha }_{{\sigma }_{\Phi }}$ with ${\sigma }_{\Phi 0}$ and ${\alpha }_{{\sigma }_{\Phi }}$ denoting the lower limit and lower tail probability of the PC hyperprior for ${\sigma }_{\gamma }$, respectively. The actual value for the standard deviation was 1 and 0.001 for the tail probability, i.e., ${\rm Pr}\left({\sigma }_{\Phi }>1\right)=0.01$. For the range $r$ we selected ${\rm Pr}\left(r<{r}_{0}\right)={\alpha }_{r}$ with ${r}_{0}$ and ${\alpha }_{r}$ denoting the upper limit and upper tail probability, respectively. We took prior information ${r}_{0}= 5{\rm km}$ which is the distance beyond which dengue disease risk is no longer spatially correlated (Sedda et al. 2018). It corresponds to the median distance between the grid cells. For the prior belief we took ${\alpha }_{r}=0.5$, i.e., ${\rm Pr}\left(r<5\right)=0.5$. Note that the two preceding decisions imply that we know the true limits ahead of the analysis.

## Identification

Because the ICAR, Leroux, AR1, RW1, and RW2 priors are specified conditionally on neighboring observations and their parameters are unique up to an additive constant, their specifications implicitly include the overall intercept. Consequently, if the model would also include an additional intercept, it would not be identified due to the perfect collinearity (Goicoa et al. 2018). To solve this identification problem, the explicit intercept can be excluded or sum-to-zero constraints on the random effects can be imposed (Goicoa et al. 2018). When sum-to-zero constraints are imposed, the random effects become orthogonal to the explicit overall intercept.

To achieve identifiability, we imposed the following sum-to-zero constraints on the temporal random of effects of the risk factors, the spatial and temporal random effects and their spatiotemporal interaction effects components:

$$\sum_{t=1}^{T}{\zeta }_{k,t}=0\quad{\rm for \,every }\,i\,{\rm and }\,g\, {\rm and} \quad {{\rm for}}\,k=1,\ldots ,K,$$

$$\sum_{i=1}^{{n}_{\mathcal{A}}}{\omega }_{i}+{\upsilon }_{i}=0\quad {\rm and }\,\sum_{i=1}^{{n}_{\mathcal{A}}}{\delta }_{it}=0 \quad {{\rm for}}\,t=1,\ldots ,T,$$

$$\sum_{t=1}^{T}{\phi }_{t}+{\varsigma }_{t}=0\quad{\rm and }\,\sum_{t=1}^{T}{\delta }_{it}=0\, \,{\rm for }\,i=1,\ldots ,{n}_{\mathcal{A}}.$$

The above-mentioned sum-to-zero constraints are imposed in INLA by setting the constr = TRUE argument to the function $f(.)$ which defines the temporal random effects of the risk factors, the spatial, temporal, and spatiotemporal interaction effect components.

## Appendix 3

The best specification of the FGG-GMRF model in Eq. (33) was selected using the deviance information criterion (DIC), the Watanabe–Akaike information criterion (WAIC), and the marginal predictive likelihood (MPL). The results are presented in Table 6.^35^ As a rule of thumb, the best model is the one with the smallest DIC and WAIC, and the largest MPL.

**Table 6:** Deviance information criterion (DIC), Watanabe–Akaike information criterion (WAIC) and marginal predictive likelihood (MPL) of a subset of models of the FGG-GMRF model in Eq. (33)^a^

Model specification^b^	Likelihood	Time-varying effect	Edge length (m)	DIC	WAIC	MPL
M1.1.1.0	Poisson	RW1	–	5184.089	5623.433	− 2850.213
M1.2.1.0	NB	RW1	–	3281.386	3283.865	− 1778.259
M1.1.2.0	Poisson	RW2	–	5229.130	5616.038	− 2893.252
M1.2.2.0	NB	RW2	–	3380.390	3381.260	− 1941.200
M2.1.1.1	Poisson	RW1	1000	2657.428	2640.887	− 1582.563
M2.2.1.1	NB	RW1	1000	2676.950	2671.954	− 1583.277
M2.1.1.2	Poisson	RW1	1100	2663.998	2660.371	− 1579.446
M2.2.1.2	NB	RW1	1100	2676.937	2671.849	− 1582.548
M2.1.1.3	Poisson	RW1	1200	2663.953	2660.405	− 1580.599
M2.2.1.3	NB	RW1	1200	2680.719	2676.344	− 1582.903
M2.1.1.4	Poisson	RW1	1300	2658.124	2642.776	− 1583.341
M2.2.1.4	NB	RW1	1300	2681.375	2675.452	− 1582.484
M2.1.1.5	Poisson	RW1	1400	2663.895	2661.152	− 1580.897
M2.2.1.5	NB	RW1	1400	2680.761	2676.639	− 1582.581
M2.1.1.6	Poisson	RW1	1500	2664.065	2661.030	− 1580.177
M2.2.1.6	NB	RW1	1500	2680.761	2676.639	− 1582.581
M2.1.2.1	Poisson	RW2	1000	2658.819	2644.598	− 1597.562
M2.2.2.1	NB	RW2	1000	2669.423	2653.978	− 1599.671
M2.1.2.2	Poisson	RW2	1100	2658.947	2642.814	− 1599.163
M2.2.2.2	NB	RW2	1100	2658.947	2642.814	− 1599.163
M2.1.2.3	Poisson	RW2	1200	2665.287	2663.565	− 1594.933
M2.2.2.3	NB	RW2	1200	2681.804	2677.917	− 1597.695
M2.1.2.4	Poisson	RW2	1300	2659.083	2643.993	− 1598.382
M2.2.2.4	NB	RW2	1300	2683.923	2679.331	− 1596.700
M2.1.2.5	Poisson	RW2	1400	2665.050	2662.933	− 1595.102
M2.2.2.5	NB	RW2	1400	2683.923	2679.331	− 1596.700
M2.1.2.6	Poisson	RW2	1500	2664.968	2662.321	− 1595.516
M2.2.2.6	NB	RW2	1500	2683.821	2679.441	− 1596.753
M3.1.1.1	Poisson	RW1	1000	2660.567	2644.342	− 1586.289
M3.2.1.1	NB	RW1	1000	2667.208	2659.532	− 1588.466
M3.1.1.2	Poisson	RW1	1100	2658.277	2641.471	− 1585.928
M3.2.1.2	NB	RW1	1100	2667.213	2659.537	− 1588.420
M3.1.1.3	Poisson	RW1	1200	2658.277	2641.472	− 1585.928
M3.2.1.3	NB	RW1	1200	2667.213	2659.537	− 1588.419
M3.1.1.4	Poisson	RW1	1300	2660.667	2644.078	− 1586.228
M3.2.1.4	NB	RW1	1300	2667.213	2659.536	− 1588.410
M3.1.1.5	Poisson	RW1	1400	2660.510	2644.422	− 1586.429
M3.2.1.5	NB	RW1	1400	2667.191	2659.512	− 1588.405
M3.1.1.6	Poisson	RW1	1500	2660.598	2644.207	− 1586.461
M3.2.1.6	NB	RW1	1500	2667.191	2659.512	− 1588.400
M3.1.2.1	Poisson	RW2	1000	2661.441	2647.045	− 1600.555
M3.2.2.1	NB	RW2	1000	2668.788	2663.003	− 1602.876
M3.1.2.2	Poisson	RW2	1100	2659.836	2645.165	− 1599.883
M3.2.2.2	NB	RW2	1100	2659.836	2645.165	− 1599.883
M3.1.2.3	Poisson	RW2	1200	2659.544	2645.097	− 1599.448
M3.2.2.3	NB	RW2	1200	2668.788	2663.003	− 1602.876
M3.1.2.4	Poisson	RW2	1300	2661.572	2646.658	− 1599.695
M3.2.2.4	NB	RW2	1300	2668.788	2663.003	− 1602.871
M3.1.2.5	Poisson	RW2	1400	2661.619	2647.490	− 1600.489
M3.2.2.5	NB	RW2	1400	2668.803	2663.020	− 1602.851
M3.1.2.6	Poisson	RW2	1500	2661.708	2647.171	− 1600.532
M3.2.2.6	NB	RW2	1500	2668.803	2663.020	− 1602.847

The second number refers to the likelihood: 1: Poisson and 2: Negative Binomial, the third to the time varying effect of the coefficients, 1: random walk of order 1 (RW1) and 2: random walk of order 2 (RW2), the fourth digit defines the edge length, 0: edge length not relevant, 1: 1000 m, 2: 1,100 m, 3: 1,200 m, 4: 1,300 m, 5: 1,400 m, and 6: 1,500 m. Note that there is no edge length for the M1 models because they do not need meshing. M1 contains 4 sub-models, and M2 and M3 contain 24 sub-models each

^a^The FGG-GMRF models are specified as ${\mathbb{E}}\left[{\mathbf{y}}_{{\varvec{t}}}|{\mathbf{E}}_{{\varvec{t}}}{{\varvec{\uptheta}}}_{\mathbf{t}}\right]$

^b^The following notation applies. M denotes Model. M1 is the sub-model with covariates only, i.e., ${{\varvec{\eta}}}_{t}={\upbeta }_{0}{1}_{({n}_{\mathcal{A}}+{n}_{p})}+{\sum }_{k=1}^{4}\left({\beta }_{k}+{\zeta }_{k,t}\right){\mathbf{z}}_{k,t}$, M2 the model with covariates plus area and cell level interaction, i.e., ${{\varvec{\eta}}}_{t}={\upbeta }_{0}{1}_{({n}_{\mathcal{A}}+{n}_{p})}+{\sum }_{k=1}^{K}\left({\beta }_{k}+{\zeta }_{k,t}\right){\mathbf{z}}_{k,t}+{{\varvec{\updelta}}}_{t}+{{\varvec{\Phi}}}_{t}$(Area interaction type IV with precision matrix ${\mathbf{Q}}_{\delta }={\tau }_{\delta }{\mathbf{R}}_{\omega }\otimes {\mathbf{R}}_{\phi }),$. M3 the full model, i.e., ${{\varvec{\eta}}}_{t}={\upbeta }_{0}{1}_{({n}_{\mathcal{A}}+{n}_{p})}+{\sum }_{k=1}^{K}\left({\beta }_{k}+{\zeta }_{k,t}\right){\mathbf{z}}_{k,t}+\ddot{{\varvec{\upomega}}}+\ddot{{\varvec{\upsilon}}}+{\phi }_{t}{1}_{({n}_{\mathcal{A}}+{n}_{p})}+{\varsigma }_{t}{1}_{({n}_{\mathcal{A}}+{n}_{p})}+{{\varvec{\updelta}}}_{t}+{{\varvec{\Phi}}}_{t}$

## Footnotes

1 Below we use the notion of “cell” to denote spatial units of higher resolution.

2 Joint area-cell estimation is based on the assumption that the same factors at area and cell level drive the spatiotemporal process, although usually in various degrees (Banerjee et al. 2015; Utazi et al. 2019). In the present case, the same disease risk factors (weather) drive disease incidence at area and cell levels.

3 Note that although the precision matrix is sparse, the covariance matrix is dense in general. Specifying models in terms of the precision matrix rather than the covariance matrix is useful in many high-dimensional applications because it allows for a sparse representation, typically reducing computational cost and memory usage (Sidén et al. 2018).

4 Although Dengue disease incidence depends on socioeconomic risk factors such as income, family size, age, education, and weather variables such as humidity, precipitation, and sunshine, the case study presented below only considers the latter, while the socioeconomic risk factors are taken into account by the random effects. The reason is that the latter are only available at city level.

5 A negative binomial distribution is appropriate when there is large overdispersion of zeros (Berk and MacDonald 2008; Payne et al. 2017).

6 Spatiotemporal variation (in disease outcomes) consists of the following three components: a spatial component capturing the overall spatial distribution, a temporal component capturing the overall temporal pattern, and a space–time component capturing space–time interaction. Spatiotemporal models are classified as separable or non-separable (Knorr-Held 2020). A spatiotemporal model is said to be separable if it consists of spatial and temporal components without their interaction, while a non-separable model comprises all three components (Haining and Li 2020; Knorr-Held 2000; Martinez-Beneito and Botella-Rocamora 2019).

7 The regression functions are latent in that they do not have pre-specified functional forms. This feature makes latent regression functions suitable to accommodate complicated relationships between the risk factors and the dependent variable.

8 According to Hall et al. (2016) and Martınez-Bello et al. (2017b), temporally varying coefficients models can be seen as distributed lag parameters. They allow the visualization and exploration of changes in the dependent variable as linear or nonlinear functions of time.

9 Random walk models are the Bayesian equivalents of P(enalized) Spline regression models (Wang et al. 2018a).

10 A GF $\gamma \left({\boldsymbol{s}}_{g},t\right)={\gamma }_{t}\left({\boldsymbol{s}}_{g}\right)$ is mean square differentiable in $\mathcal{A}$, if for every ${\boldsymbol{s}}_{g}\,{\rm in}\,\mathcal{A}$ and for every *t*, ${\gamma }_{t}^{{\prime}}\left({\boldsymbol{s}}_{g}\right)=\underset{l\to 0}{{\rm lim}}\frac{{\gamma }_{t}\left({\boldsymbol{s}}_{g}+l\right)-{\gamma }_{t}\left({\boldsymbol{s}}_{g}\right)}{l}$ exists and $\underset{l\to 0}{{\rm lim}}\mathbb{E}{\left[\frac{{\gamma }_{t}\left({\boldsymbol{s}}_{g}+l\right)-\gamma \left({\boldsymbol{s}}_{g}\right)}{l}-{\gamma }_{t}^{{'}}\left({\boldsymbol{s}}_{g}\right)\right]}^{2}=0$ (Stein 1999).

11 The inla.stack function can be used to define the fusion model framework.

12 A joint prior may refer to multiple parameters, to the spatial units (area and cell), to multiple time periods, or to space–time. The specification of the parameter vector or the subscripts of the precision matrices indicate what kind of jointness is in order.

13 The total number of hyperparameters is $J=2K+11$, where $K$ is the total number of risk factors and 11 represents the total of the elements of {${\sigma }_{{\beta }_{0}}^{2},{\sigma }_{\upomega }^{2}, {\sigma }_{\upsilon }^{2},{\sigma }_{\phi }^{2},{\sigma }_{\boldsymbol{\varsigma }}^{2},{\sigma }_{\Phi }^{2},{\sigma }_{\updelta }^{2}, \rho ,{\lambda }_{1}, {\lambda }_{2},r$}.

14 A sparse precision matrix enables the application of computationally efficient numerical methods. For instance, for the factorization of a dense covariance matrix of a spatiotemporal GF, the ${\rm O}({n}^{3}$) computation time is reduced to ${\rm O}({n}^{2}$) for a sparse covariance matrix of a GMRF (Rue and Held 2005).

15 The LSPDE approach uses finite basis functions defined by a triangulation of the study region (see Online Resource 1 for application to Bandung city) to transform a GF to a GMRF. Implementation can be conveniently handled using INLA (Rue et al. 2009). The LSPDE approach is immediately applicable to a large class of spatiotemporal models. For instance, it applies to models with complex hierarchical structures or with non-separable covariance functions, as well as non-stationary models with time-varying parameters (Cameletti et al. 2013).

16 The number of nodes (vertices) $L$ is larger than the number of cells ${n}_{p}$ because we extend the domain of interest by an outer area to avoid the boundary effect (see Appendix 1 for details).

17 The main objective of this research is to find out in which areas and which months Dengue outbreaks will occur annually. For this purpose monthly cycle observations suffice.

18 R-INLA contains the routine inla.mesh.2d() for meshing of spatial domains. It contains several subroutines, notably loc: for the number and the location of the initial mesh vertices, and max.edge: for the maximum edge lengths in the study area and in the outer area.

19 In Jaya and Folmer (2021a), the order of estimation was reverse. The models with the structurally and temporally main random effects were estimated before the models with the interaction effects. The latter outperformed the former. Based on this outcome, we estimated the M2 models before the M3 models to economize on computation time.

20 The estimates of the models with other types of interaction are available from the first author upon request.

21 For a precise definition of the models see the note under Table 6, Appendix 3.

22 The reason that some cells are partly outside some subdistrict boundaries is that the cells are squares.
