# Supplementary material for: Spatiotemporal high-resolution prediction and mapping: methodology and application to dengue disease
Source: J Geogr Syst. 2022 Feb 19;24(4):527–81. doi: 10.1007/s10109-021-00368-0 (PMC8857957; doi:10.1007/s10109-021-00368-0)
Supplement: Supplementary file 1 — (PDF 210 KB) [file 10109_2021_368_MOESM1_ESM.pdf]

Online Resource 1

Table A.1 Districts labels and population at risk

id	District	Population at Risk by Year						
		2012	2013	2014	2015	2016	2017	2018
1	Gedebage	439,884	444,984	451,884	451,884	451,884	451,884	451,884
2	Ujungberung	912,252	922,824	945,600	945,600	945,600	945,600	945,600
3	Cinambo	299,304	302,772	310,272	310,272	310,272	310,272	310,272
4	Bandung Kulon	1,689,360	1,708,932	1,780,536	1,780,536	1,780,536	1,780,536	1,780,536
5	Andir	1,157,220	1,170,636	1,215,744	1,215,744	1,215,744	1,215,744	1,215,744
6	Babakan Ciparay	1,744,932	1,765,152	1,839,120	1,839,120	1,839,120	1,839,120	1,839,120
7	Bojongloa Kaler	1,428,300	1,444,860	1,505,364	1,505,364	1,505,364	1,505,364	1,505,364
8	Sukajadi	1,285,596	1,300,500	1,350,516	1,350,516	1,350,516	1,350,516	1,350,516
9	Cidadap	695,988	704,064	728,448	728,448	728,448	728,448	728,448
10	Coblong	1,560,276	1,578,360	1,641,564	1,641,564	1,641,564	1,641,564	1,641,564
11	Cicendo	1,183,308	1,197,024	1,243,176	1,243,176	1,243,176	1,243,176	1,243,176
12	Bandung Wetan	369,204	373,488	385,956	385,956	385,956	385,956	385,956
13	Sumurbandung	433,920	438,948	449,808	449,808	449,808	449,808	449,808
14	Batununggal	1,434,492	1,451,124	1,506,852	1,506,852	1,506,852	1,506,852	1,506,852
15	Cibeunying Kidul	1,278,852	1,293,672	1,345,440	1,345,440	1,345,440	1,345,440	1,345,440
16	Regol	966,408	977,604	1,018,608	1,018,608	1,018,608	1,018,608	1,018,608
17	Bandung Kidul	699,384	707,484	737,136	737,136	737,136	737,136	737,136
18	Astanaanyar	816,504	825,960	858,300	858,300	858,300	858,300	858,300
19	Kiaracondong	1,565,520	1,583,664	1,644,456	1,644,456	1,644,456	1,644,456	1,644,456
20	Buahbatu	1,128,216	1,141,296	1,186,224	1,186,224	1,186,224	1,186,224	1,186,224
21	Mandalajati	754,188	762,936	788,016	788,016	788,016	788,016	788,016
22	Cibiru	854,292	864,192	882,924	882,924	882,924	882,924	882,924
23	Bojongloa Kidul	1,016,232	1,028,016	1,072,380	1,072,380	1,072,380	1,072,380	1,072,380
24	Cibeunying Kaler	841,332	851,088	885,192	885,192	885,192	885,192	885,192
25	Panyileukan	477,444	482,976	493,536	493,536	493,536	493,536	493,536
26	Antapani	883,296	893,532	927,876	927,876	927,876	927,876	927,876
27	Lengkong	844,452	854,244	890,052	890,052	890,052	890,052	890,052
28	Sukasari	971,652	982,896	1,020,708	1,020,708	1,020,708	1,020,708	1,020,708
29	Rancasari	912,168	922,740	945,516	945,516	945,516	945,516	945,516
30	Arcamanik	822,228	831,756	854,520	854,520	854,520	854,520	854,520

Meshing

To transform the GG-GF model with a dense Matérn covariance matrix to a GG-GMRF model with a sparse Matérn covariance matrix, the Linear Stochastic Partial Differential Equation (LSPDE) was solved by the Finite Element Method (FEM) based on triangulation of the spatial domain (Bandung city). We constructed the mesh using the prediction cells and a non-convex hull bounding them. We considered six different triangle meshes with six different edge lengths starting from 1 km up to 1.5 km, with a difference of 0.1 km. To avoid boundary effects the domain was extended using a lower triangle resolution for the outer area (Blangiardo and Camalety 2015). We selected a fixed edge length of 3 km for the outer area. Based on the DIC and WAIC criterion, the mesh with a 1.5 km edge length was found to be the optimal mesh.

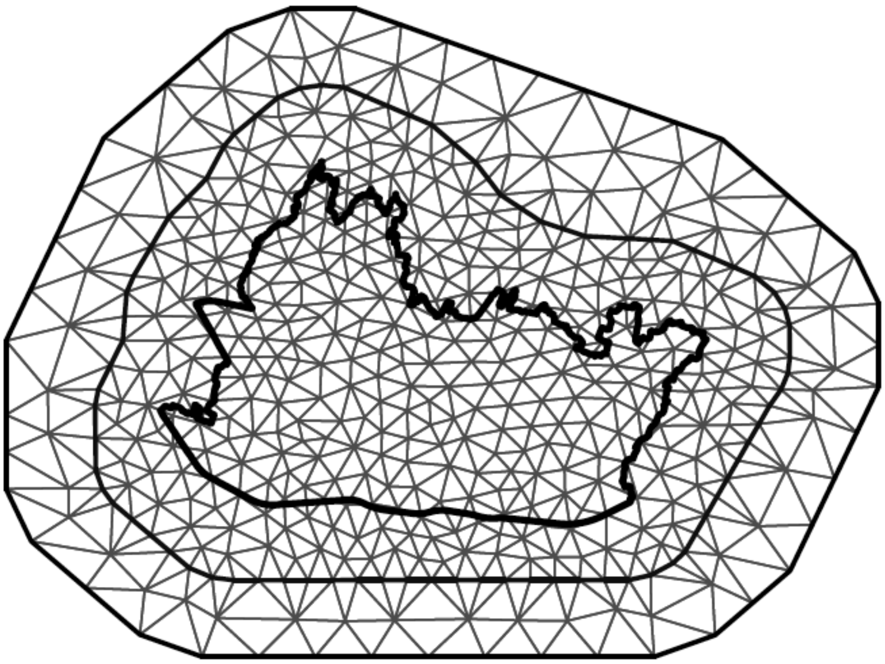

Figure A.1 Triangle mesh of Bandung city (in km) with maximum edge 1.5 km and number of vertices $L = 501$
